# Direct estimation of *de novo* mutation rates in a chimpanzee parent-offspring trio by ultra-deep whole genome sequencing

**DOI:** 10.1038/s41598-017-13919-7

**Published:** 2017-11-01

**Authors:** Shoji Tatsumoto, Yasuhiro Go, Kentaro Fukuta, Hideki Noguchi, Takashi Hayakawa, Masaki Tomonaga, Hirohisa Hirai, Tetsuro Matsuzawa, Kiyokazu Agata, Asao Fujiyama

**Affiliations:** 1 Department of Brain Sciences, Center for Novel Science Initiatives, National Institutes of Natural Sciences, Okazaki, Aichi 444-8585 Japan; 2 Department of System Neuroscience, National Institute for Physiological Sciences, Okazaki, Aichi 444-8585 Japan; 3 Department of Physiological Sciences, School of Life Science, SOKENDAI (The Graduate University for Advanced Studies), Okazaki, Aichi 484-8585 Japan; 4 Center for Genome Informatics, Joint Support-Center for Data Science Research, Research Organization of Information and Systems, Mishima, Shizuoka, 411-8540 Japan; 5 Advanced Genomics Center, National Institute of Genetics, Mishima, Shizuoka, 411-8540 Japan; 6 Department of Wildlife Science (Nagoya Railroad Co., Ltd.), Primate Research Institute, Kyoto University, Inuyama, Aichi 484-8506 Japan; 7 Japan Monkey Centre, Inuyama, Aichi 484-0081 Japan; 8 Language and Intelligence Section, Department of Cognitive Sciences, Primate Research Institute, Kyoto University, Inuyama, Aichi 484-8506 Japan; 9 Molecular Biology Section, Department of Cellular and Molecular Biology, Primate Research Institute, Kyoto University, Inuyama, Aichi 484-8506 Japan; 10 Institute of Advanced Study, Kyoto University, Kyoto, 606-8501 Japan; 11 Laboratory for Biodiversity, Global COE Program, Graduate School of Science, Kyoto University, Kyoto, 606-8502 Japan; 12 Laboratory for Molecular Developmental Biology, Graduate School of Science, Kyoto University, Kyoto, 606-8502 Japan; 13 Graduate Course in Life Science, Gakushuin University, Tokyo, 171-8585 Japan; 14 Department of Genetics, School of Life Science, SOKENDAI (The Graduate University for Advanced Studies), Mishima, Shizuoka, 411-8540 Japan

## Abstract

Mutations generate genetic variation and are a major driving force of evolution. Therefore, examining mutation rates and modes are essential for understanding the genetic basis of the physiology and evolution of organisms. Here, we aim to identify germline *de novo* mutations through the whole-genome surveyance of Mendelian inheritance error sites (MIEs), those not inherited through the Mendelian inheritance manner from either of the parents, using ultra-deep whole genome sequences (>150-fold) from a chimpanzee parent-offspring trio. We identified such 889 MIEs and classified them into four categories based on the pattern of inheritance and the sequence read depth: [i] *de novo* single nucleotide variants (SNVs), [ii] copy number neutral inherited variants, [iii] hemizygous deletion inherited variants, and [iv] *de novo* copy number variants (CNVs). From *de novo* SNV candidates, we estimated a germline *de novo* SNV mutation rate as 1.48 × 10^−8^ per site per generation or 0.62 × 10^−9^ per site per year. In summary, this study demonstrates the significance of ultra-deep whole genome sequencing not only for the direct estimation of mutation rates but also for discerning various mutation modes including *de novo* allelic conversion and *de novo* CNVs by identifying MIEs through the transmission of genomes from parents to offspring.

## Introduction

Estimation of mutation rates and identification of mutation modes are important for better understanding the molecular mechanisms of an organism’s physiological conditions and the species’ evolutionary history. Advancements of high-throughput next-generation sequencing (NGS) technologies and their application to the whole genome sequencing (WGS) of a large number of human genomes revealed the mutation spectrum, genetic diversity, and population history of human beings^1–3^. As for the mutation spectrum, recent studies utilizing the WGS data from multiple human parent-offspring trios or quartets (pedigree-based approach) estimated germline *de novo* mutation rates for single nucleotide variants [*de novo* single nucleotide variants (SNVs)] around 0.97–1.20 × 10^−8^ per site per generation or approximately 0.38–0.48 × 10^−9^ per site per year, assuming a 25-year generation time^4–8^.

One traditional method to estimate mutation rate is a phylogenetic approach that uses the sequence divergence between two species and their ancestral effective population size. Many studies have reported a typical value of 1 × 10^−9^ per site per year as so-called “phylogenetic mutation rate^9–11^” based on the sequence divergence of 1.23–1.37% between humans and chimpanzees^11–13^ and an assumed sequence divergence time approximately 6–7 million years ago (Ma); however, uncertain factors such as extent of ancestral polymorphisms, effective population size, generation time, and rate of heterogeneity within and between the genomes of species are associated with the method^14,15^.

To overcome these difficulties and to estimate the mutation rates more directly, we performed WGS on the genomes of a chimpanzee parent-offspring trio and then identified *de novo* SNVs and other structural alterations. The chimpanzee parent-offspring trio used in this study have been participating in a wide variety of comparative cognitive research since 1978^16,17^. Because the frequency of *de novo* SNVs and structural alterations found within the single generation should be very low^4–8^, we took a straightforward strategy to identify such events through ultra-deep WGS to compensate for statistical variation and sequencing errors. In total, we acquired 150-fold coverage of the sequences of all individuals. To the best of our knowledge, this is the first study to conduct such an ultra-deep WGS of a given mammalian parent-offspring trio. In addition to the identification of the *de novo* SNV sites, we were able to detect and identify *de novo* copy number variation sites (CNVs) among the trio according to the comparison of the depth of the sequence read coverage in a given region. Moreover, although little is known about the biological significance of *de novo* allelic conversion (known as interallelic gene conversion), we succeeded in the quantifying the rate of genome-wide *de novo* allelic conversion events.

## Results

### Comprehensive and highly accurate identification of structural variants through ultra-deep whole genome sequencing

**Figure 1:**
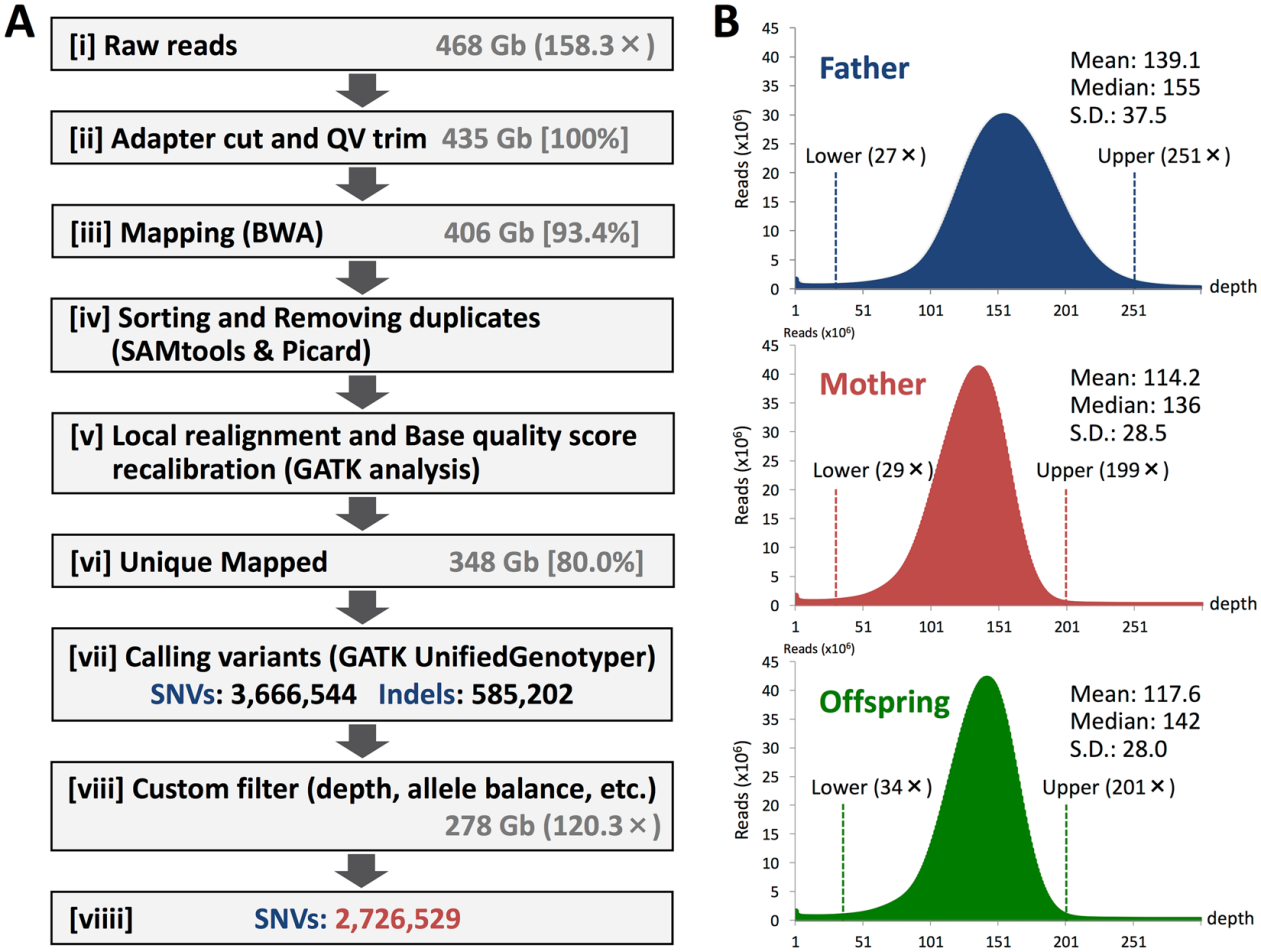
Whole-genome sequencing (WGS) and workflow of variant discovery. (**A**) Pipeline for mapping and variant detection. The offspring’s data are shown in the box. (**B**) Distribution of the read-depth within the datasets from the chimpanzee trio. Lower and upper read-depths shown in each histogram indicates ± 3σ from the mean, and the reads present in the outlier regions were excluded from the following analyses.

To understand the mechanism of the structural changes of genomes and to estimate their rates of occurrence from parents to offspring, it is essential to detect with the highest possible accuracy the structural changes in the genome of each parent-offspring member. In the present study, we sequenced the genomes of a mother-father-offspring (male) chimpanzee trio reared at the Primate Research Institute, Kyoto University (Methods). We acquired the raw DNA sequences of 575 gigabases (Gb) with 194.6-fold genome coverage against the total number of non-N bases of the chimpanzee reference sequence (CHIMP2.1.4 or panTro4), 463 Gb with 157.8-fold coverage, and 468 Gb with 158.3-fold coverage of the father, mother, and son, respectively (Fig. 1A, Supplementary Table S1). The distributions of the read depth of the chimpanzee trio are shown in Fig. 1B. The raw data were processed to extract high-quality reads and mapped to the chimpanzee reference genome to identify the positions of structural variant candidates as an initial dataset (Fig. 1A; Methods).

In total, we detected approximately 3.67 million SNVs and 585 thousand insertion/deletions (indels) over 89.16% of the reference genome for the trio [(vii) in Fig. 1A, Table 1]. These initially obtained candidate sites were further examined to minimize systematic errors and false positives (FPs) for the accuracy. For SNVs, for example, we excluded low-complexity or repetitive regions from the alignment using the following filters: (i) read depth at each nucleotide position, (ii) balance between forward and reverse reads at a particular site, (iii) indels, (iv) allelic and strand biases, and (v) positions flanking to gaps (see Methods and Supplementary Method for details). In addition, we only considered autosomes. Table 1 demonstrates a total number of SNVs after filtering. The frequency of SNVs for each trio member exhibits almost the same value (0.118%), and the autosomal heterozygosity was 0.076% in any individual (Table 1, Supplementary Table S2), which coincided with the reported values for the Western chimpanzee, 0.08%^13^ or 0.077–0.084%^18^, and even that of human, 0.0765%^19^. The ratio of transition to transversion (Ti/Tv) is 1.98 for the trio (Table 1), which is 2.0–2.1 for the human genome^20^.

**Table 1:** Summary of SNVs.

Individual	Father	Mother	Offspring
**Depth**	27 ≤ depth ≤ 251	29 ≤ depth ≤ 199	34 ≤ depth ≤ 201
**No. of homo SNVs (autosome)**	977,567	968,196	975,445
**No. of hetero SNVs (autosome)**	1,748,513	1,767,067	1,751,084
**No. of total SNVs (autosome)**	2,726,080	2,735,263	2,726,529
**%SNV (autosome)**	0.118	0.118	0.118
**%Heterozygosity (autosome)**	0.076	0.076	0.076
**No. of transition SNVs (Ti) (autosome)**	1,810,503	1,818,242	1,811,915
**No. of transversion SNVs (Tv) (autosome)**	915,577	917,021	914,614
**Ti/Tv (autosome)**	1.98	1.98	1.98
**[%] Genome covered w/o N bases (Common)**	89.16		
**[%] CDS coverage (Common)**	93.05		

### Identification of *de novo* SNVs in the genome of the offspring

**Figure 2:**
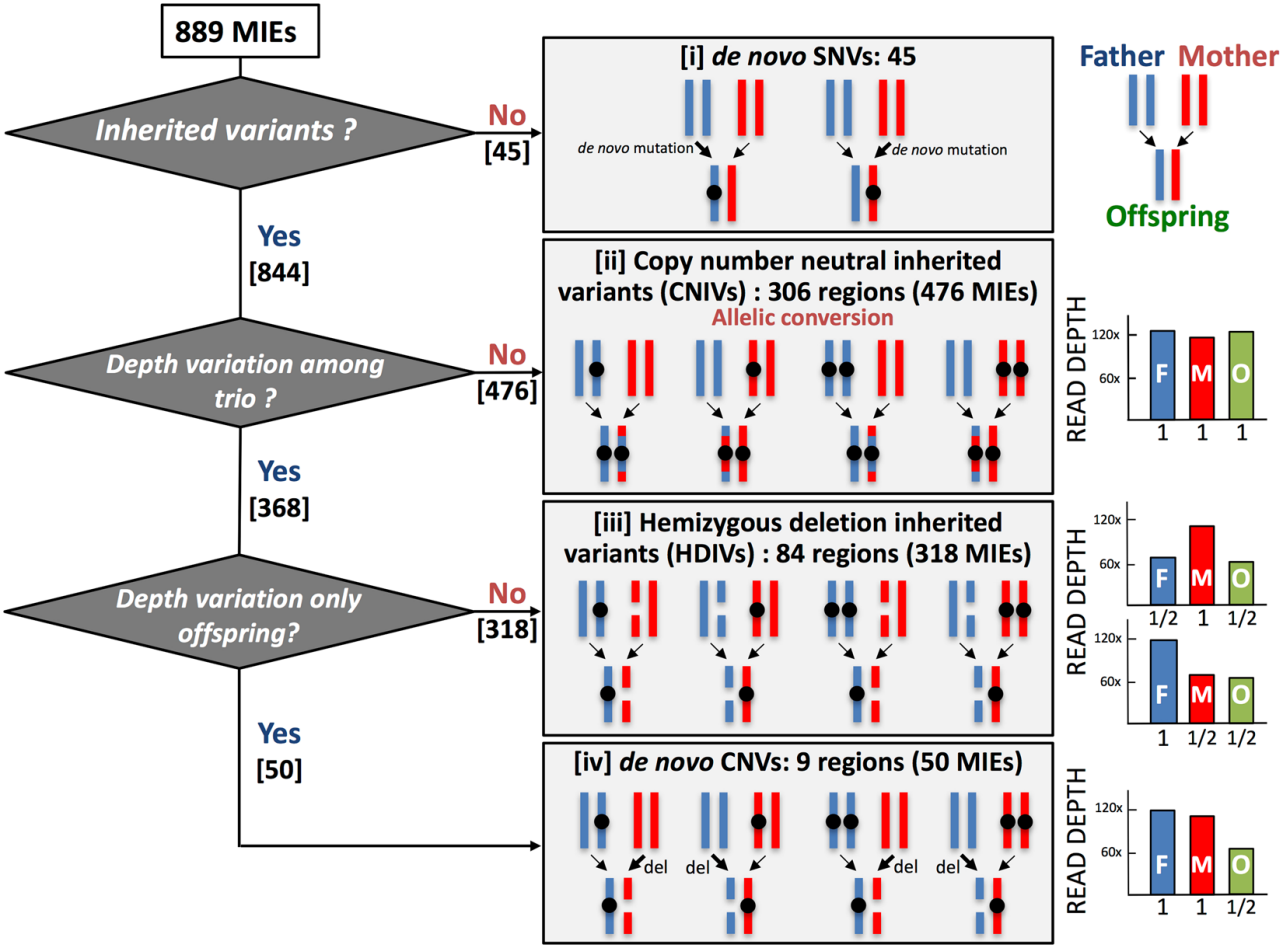
Classification of the MIEs. When the variant alleles were identified only in the offspring, they were classified as [i] *de novo* SNVs. Inherited MIEs are classified into [ii] copy-number neutral inherited variants (CNIVs), [iii] hemizygous-deletion inherited variants (HDIVs), and [iv] *de novo* CNVs, according to the relative depth of the read-coverage among the trio’s sequences. Black circles indicate the sites of SNVs. The vertical columns in the right panel represent schematics of the read-coverage and their relative ratios.

The main purpose of this study is to identify and analyze the genetic signature of mutations in the framework of WGS of a chimpanzee parent-offspring trio. To achieve this goal, we aimed to identify the sites that were not inherited from either parent through Mendelian inheritance, which was referred to as Mendelian inheritance errors (MIEs)^4^. Using the total set of SNV calls obtained in initial analyses (Table 1), we analyzed inheritance in the trio and identified 2,405 sites in the genome of the offspring as MIEs. We excluded those located in repetitive regions, such as LINE/SINEs, simple repeats, and LTRs to improve accuracy (Supplementary Table S3), and the remaining 889 MIEs were further classified into four categories based on the pattern of inheritance and the coverage depth of the mapped reads to the corresponding region: [i] *de novo* SNVs, [ii] copy number neutral inherited variants (CNIVs), [iii] hemizygous deletion inherited variants (HDIVs), and [iv] *de novo* CNVs as shown in Fig. 2.

Finally, we identified 45 *de novo* SNVs among 889 MIEs ([i] in Fig. 2, Supplementary Figure S1 and Table S4). Out of the 45 *de novo* SNVs, 20, 24, and 1 SNVs were found in intergenic, intronic, and exonic regions, respectively. This is consistent with the rate of *de novo* SNVs reported for the human exome (0.92 *de novo* SNVs on average in exonic regions)^21^.

### Characterization of copy number neutral inherited variants and hemizygous deletion inherited variants

Other than the *de novo* SNVs, we discovered that 476 and 318 MIEs were classified into CNIVs and HDIVs, respectively, based on the relative read depth among the trio (Fig. 2, Supplementary Figure S1). Since we sequenced each chimpanzee genome with more than 150-fold genome coverage, we were able to detect, distinguish, and quantify CNIVs and HDIVs through the comparison of the read depth at each candidate site (see Methods for detail). When the read depth showed similar extent among the trio throughout the corresponding genomic regions, as shown in [ii] of Fig. 2, we assumed that an allelic conversion occurred through the transmission of genomes from either parent to the offspring. In contrast, when the read depth varied considerably among the trio (either father/offspring or mother/offspring had half read depth in a given site/region as shown in [iii] of Fig. 2), we assumed the deletion of one allele (known as a hemizygous state) in father/offspring or in mother/offspring in a given site/region as described in ref.^2^. Therefore, we defined these MIEs as inherited variants and not *de novo* SNVs.

When multiple CNIVs were closely located on the genome, those CNIVs could be generated from a single allelic conversion event. If we suppose that 476 CNIVs randomly occurred on the target genomic regions (1.17 × 10^9^ bp in this study), the expected mean distance of two adjacent CNIVs was 2.46 × 10^6^ bp, and the 99% confidence interval of the distance was calculated from 1.75 × 10^4^ bp to 1.25 × 10^7^ bp based on the 10,000 bootstrap resampling simulation. Then, we assumed a single allelic conversion event if two adjacent CNIVs are significantly closely located at each other. We set the criteria to a lower bound of a 99% confidence interval (1.75 × 10^4^ bp). Indeed, more than half of the CNIVs have an adjacent CNIV within 1.75 × 10^4^ bp (238/476), and especially 128 and 192 CNIVs have an adjacent CNIV within less than 100 bp and 1,000 bp, respectively (Supplementary Figures S1B, S2 and S3), strongly suggesting that most of the CNIVs are closely located to each other and are likely to be the products of a single allelic conversion event. As a result, we identified 306 such events from 476 CNIVs (Supplementary Table S4) and estimated the rate of the genome-wide *de novo* allelic conversion rate as an order of 10^−7^ per site per generation. However, the true allelic conversion rate could be higher than the value we estimated here because we were unable to identify conversion events when two alleles have long identical DNA sequences due to no marker for distinguishing them. For the more precise estimation of the allelic conversion rate, we need to obtain much variation data and meaningful markers using multiple family trios such as the studies recently reported^22,23^.

Similarly, we found 318 HDIVs located within 84 regions (Supplementary Figure S1). A typical example of the HDIV cluster can be seen on chromosome 6 and extends 71 kb from the position of 55,271,096 bp to 55,342,281 bp in which both the mother and offspring have one copy of an allele. Across this region, genotypes of offspring are identical to those of the father because only the paternal allele is transmitted to the offspring (Supplementary Table S4).

### Characterization of *de novo* CNVs

**Figure 3:**
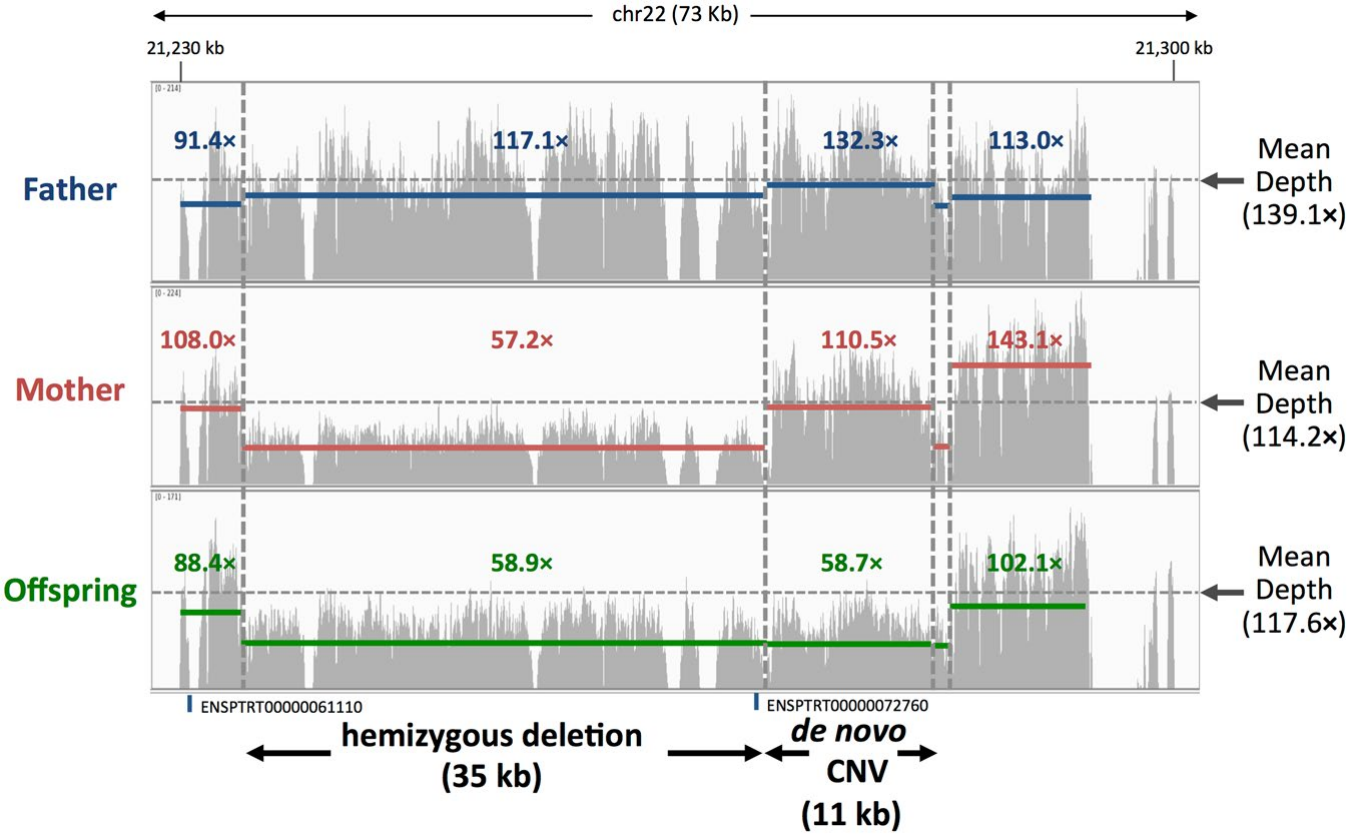
Representative region of hemizygous deletion and a *de novo* CNV on chromosome 22. Blue, red, and green lines represent the average depth of the read coverage for the corresponding regions in the father, mother, and offspring, respectively.

The final category in Fig. 2 [iv] is *de novo* CNVs, where only the offspring had half read depth. We detected nine such sites in this study (Supplementary Figure S1). In all the cases, one allele was lost from the offspring, and most of them were caused by microdeletions shorter than 6 kb. The remaining was relatively large, covering approximately 11 kb on chromosome 22, and was located adjacent to the 35 kb hemizygous deletion region (Fig. 3), where the depth of coverage for both the mother (red line) and offspring (green line) was approximately half of the mean coverage. Although the frequency is relatively low, the *de novo* CNVs may have a larger influence than that of *de novo* SNVs due to a larger extent of affected sequences.

In the present study, we searched for *de novo* CNVs with ± 3σ deviations from the mean (from 34 × to 201 × coverage) in the genome of the offspring (Fig. 1B). Because we filtered out highly repetitive regions from our analyses, we were unable to exclude the possibility of high-copy number *de novo* CNVs; however, we believe this is unlikely because all the *de novo* CNVs showed decreased copy number in the range used in this study.

### Identification of germline *de novo* SNVs

The *de novo* SNVs we initially identified in the offspring (Fig. 2) may have resulted from mutations that occurred either in germline cells in the parents or somatic cells in the offspring or both. To distinguish germline *de novo* SNVs from the somatic ones, we analyzed another DNA sample obtained from hair follicles of the offspring [mesoderm (blood) *vs*. ectoderm (hair follicle) comparison]. Because somatic mutations, if any, should occur independently in the genomes of stem cells during the development and aging processes of the offspring, they should thus produce different SNV profiles, whereas germline mutations or mutations that occurred in the early developmental stages should be retained commonly among the DNA from the tissues of different cell lineages.

**Figure 4:**
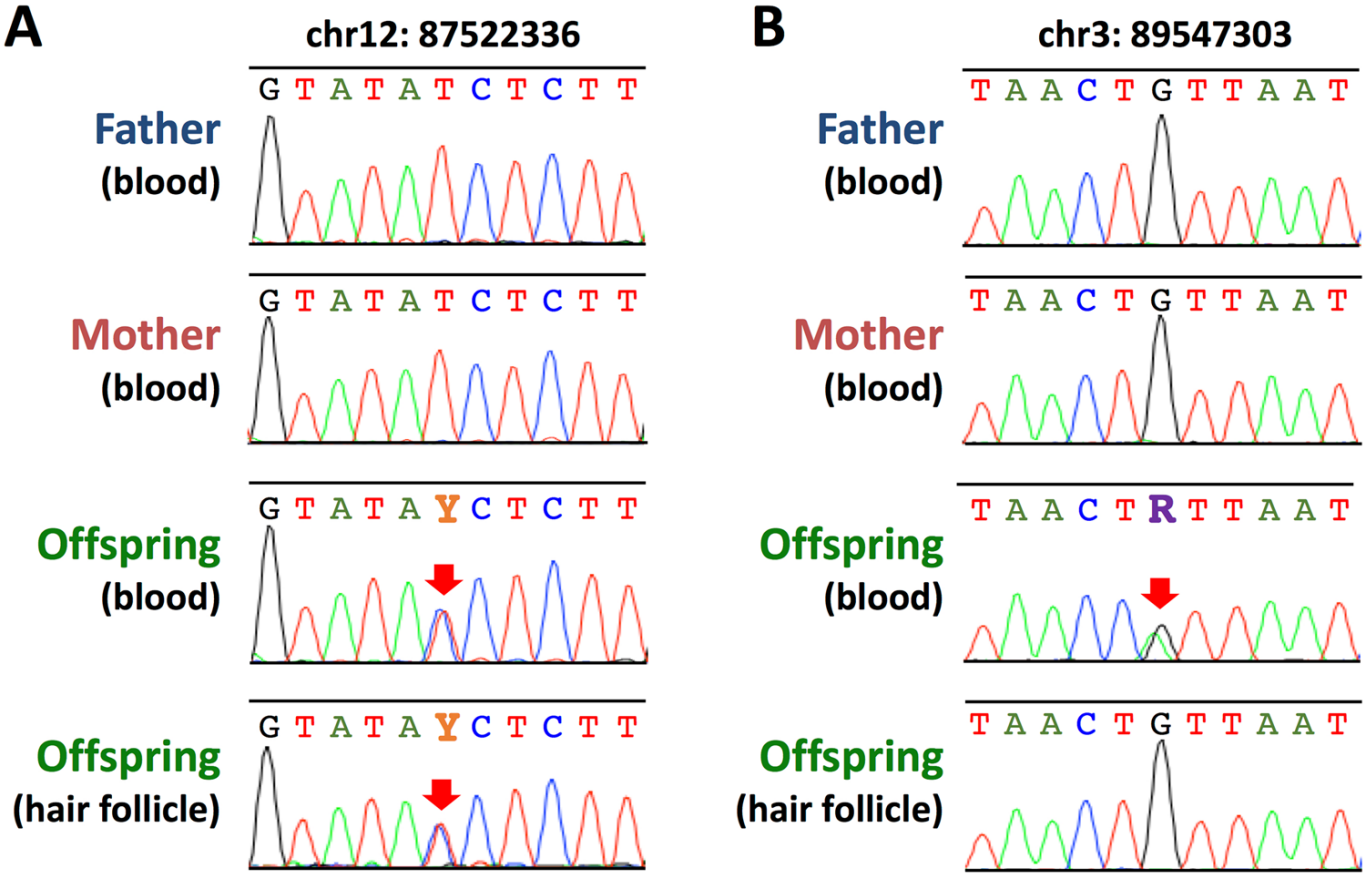
Representative Sanger sequencing electropherogram at the position of *de novo* SNVs. (**A**) An example of germline de novo SNV identified on chromosome 12, where the parents’ genotypes are homozygous and those of the blood and hair follicle DNAs of the offspring are heterozygous (red arrow). (**B**) A somatic de novo SNV identified on chromosome 3, where the only blood-derived DNA of the offspring shows heterozygous (red arrow).

Primers used for polymerase chain reaction (PCR) were designed for all 45 *de novo* SNVs, and we were able to obtain 40 PCR products across the parent-offspring trio. Subsequent genotyping of the offspring using Sanger sequencing showed differences between the genotypes of blood and hair follicle DNAs in only one case (Supplementary Table S5). As a result, almost all the *de novo* SNVs (31/32) detected in the present study are germline mutations (Fig. 4A) except for one somatic *de novo* mutation (Fig. 4B).

### Estimation of false positive and false negative rates during the process of *de novo* SNVs identification

It is also important to estimate the extent of the false positive (FP) and false negative (FN) calls and to discriminate germline *de novo* SNVs from somatic ones in our identified *de novo* SNVs. For the estimation of FP calls, we compared the Sanger sequencing data, which was collected in the previous section, with the corresponding NGS data to detect inconsistencies in the genotypes. As a result, we found eight FP calls in the 40 genotypes, yielding an FP rate to be 0.2 (8/40) (Table 2, Supplementary Table S5).

**Table 2:** False positive SNVs identified from the DNAs of blood and hair follicle cells using NGS and Sanger sequencing.

chr	position	panTro4	Father Blood NGS	Mother Blood NGS	Offspring Blood NGS	Father Blood Sanger	Mother Blood Sanger	Offspring Blood Sanger	Offspring Hair Sanger	Call*
chr1	13552700	C	CC	CC	CT	CC	CC	CC	CC	FP
chr2A	102577476^#^	C	CT	CC	CG	CGT^#^	CC	CGT^#^	CGT^#^	FP
chr6	7711997	A	AG	AA	AT	AG	AA	AG	AG	FP
chr6	12022852	G	GG	GG	GT	GG	GG	GG	GG	FP
chr6	33261071	T	TT	TT	TA	TA	TA	TA	TA	FP
chr12	14055837	T	TT	TT	TA	TA	TT	TA	TA	FP
chr12	28800658	C	CC	CC	CT	CC	CC	CC	CC	FP
chr22	22163245^#^	G	GC	GG	GA	GA	GG	GA	GA	FP

*FP: false positive, ^#^Known segmental duplication regions in chimpanzees^30^.

For the estimation of FN calls in our analysis, we used a likelihood-based program, DeNovoGear^24^, on the same data set for the comparison. DeNovoGear is a program designed to detect *de novo* mutations using NGS data as we have done in this study. When the posterior probability for the detection of *de novo* SNVs was set to 0.99 as a threshold, the DeNovoGear reported 61 sites as *de novo* SNVs, and 26 of them were not identified from our analysis (Supplementary Figure S4). To examine the sensitivity and specificity of the two methods, we performed resequencing analysis with PCR and Sanger sequencing using the parent-offspring DNA samples and confirmed the genotype of candidate *de novo* SNV sites that are inconsistent between the two methods. Out of the 26 sites that were called as *de novo* SNVs by DeNovoGear, but were not called by our analysis, seven sites were successfully genotyped by PCR and Sanger resequencing. We found that all of them were not *de novo* SNVs and then regarded all of them as true negatives (Table 3, Supplementary Figure S4, Supplementary Table S6). We could not properly genotype the rest of the candidate sites (26 − 7 = 19) because of the multiple PCR products; we speculated that these sites originated from the duplicated regions that were omitted in the present chimpanzee reference sequence. From these results, we estimated that the FN rate of our procedure is close to zero. In conclusion, we estimated that the number of FP and FN calls as 9 (45 × 0.2) and 0 (45 × 0), respectively, and identified a somatic *de novo* SNV (Fig. 4B) out of the 45 candidates *de novo* SNV sites.

**Table 3:** *De novo* SNVs identified only by DeNovoGear and genotypes determined by NGS and Sanger sequencing.

chr	position	panTro4	Father Blood NGS	Mother Blood NGS	Offspring Blood NGS	Father Blood Sanger	Mother Blood Sanger	Offspring Blood Sanger	Call*
chr1	2694332	T	CC	CC	CT	CC	CC	CC	TN
chr3	201706151	T	TT	TT	CT	TT	TT	TT	TN
chr6	73652409	A	AA	AA	AC	AA	AC	AC	TN
chr8	29532927	A	AA	AA	AG	AG	AA	AG	TN
chr15	21679458	T	TT	TT	CT	CT	TT	CT	TN
chr17	34345520	A	AA	AA	AC	AC	AA	AC	TN
chr19	56060294	T	TT	TT	CT	TT	TT	TT	TN

*TN: true negative.

### Estimation of the paternity and maternity of the *de novo* SNVs

According to the previous studies on humans, approximately 73–80% of *de novo* SNVs originate from the father^25^. In this study, we acquired plenty of paired-end sequences that enabled us to distinguish the parental origins of *de novo* SNVs using the information on the nearby heterozygous SNV sites covered by the paired read. We assigned 11 and four out of 45 *de novo* candidate SNVs, and seven and two of 31 validated *de novo* SNVs to the father and mother, respectively, showing that 73–78% *de novo* SNVs were of paternal origin. However, we should take into consideration the effect of the father’s age at conception because the number of germ cell divisions in a human male is approximately 35, 380, and 840 at ages 15, 30, and 50, respectively^26^.

### Estimation of the rate of *de novo* SNVs in germline cells

The rate of germline *de novo* SNVs per haploid genome can be calculated as follows:

$$[{\rm{number}}\,{\rm{of}}\,{\rm{germlinede}}\,{\rm{novoSNVs}}]/[{\rm{target}}\,{\rm{genomic}}\,{\rm{size}}\times 2]$$

From the numbers of the FP and FN calls (nine and zero, respectively) and the experimental confirmation that almost all of the *de novo* SNVs detected in this study are germline mutations except for a somatic *de novo* SNV, we estimated the number of germline *de novo* SNVs in this chimpanzee trio to be 35 [45–9 (number of FP calls) –1 (number of somatic *de novo* SNV)], and the range is from 31, in which all unannotated *de novo* SNVs are assigned to be false positives, to 36, in which all unannotated ones are true positives. Since the target genomic regions used in this study are 1.182 × 10^9^ bp as described before, the rate per haploid genome is calculated as follows:

$$35/[1.182\times {10}^{9}\times 2]=1.48\times {10}^{-8}/{\rm{site}}/{\rm{generation}}$$

The range is from 1.31 × 10^−8^ to 1.52 × 10^−8^ when minimum and maximum number of germline *de novo* SNVs are assumed to be 31 and 36, respectively.

According to the record, the ages of the father and mother were estimated to be 24 years when their offspring was born. Therefore, we speculated that the germline *de novo* SNVs occur with a frequency of 0.62 × 10^−9^ per site per year, which is slightly higher than the pedigree-based rate for humans and chimpanzees^27^.

## Discussion

**Figure 5:**
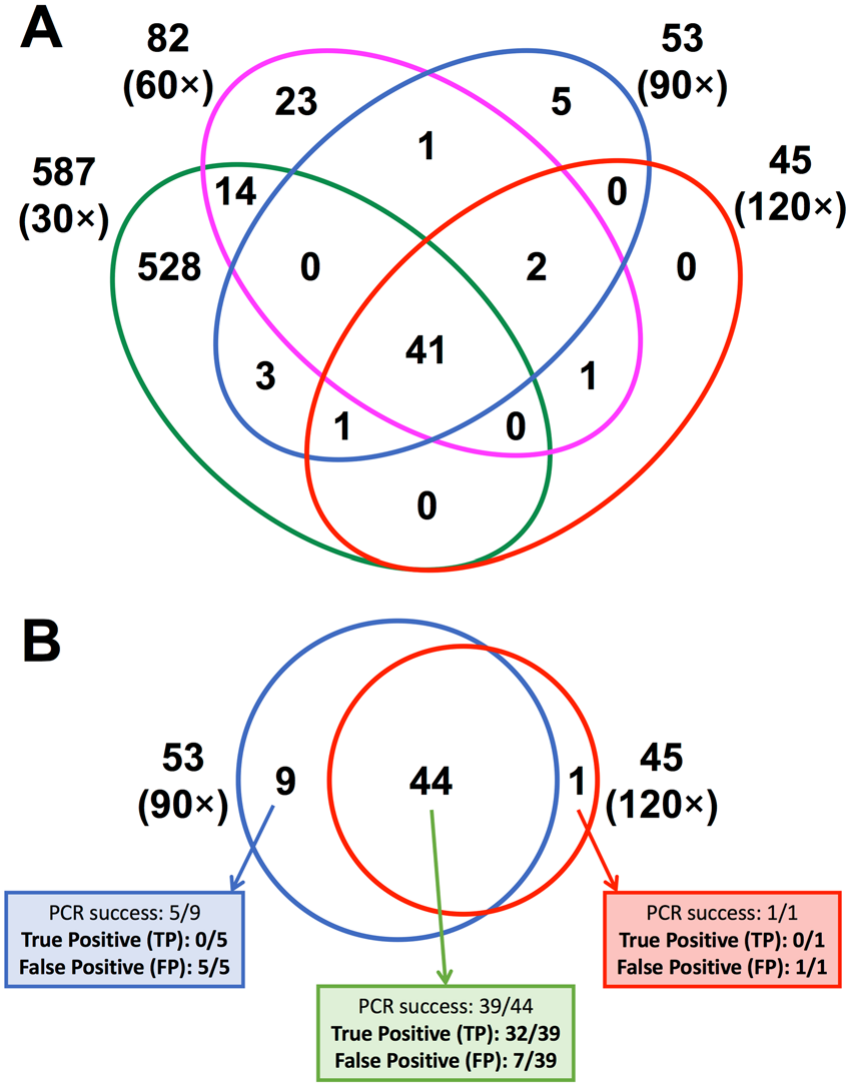
Number of candidate de novo SNV site among four different depth of sequencing coverage data (30×, 60×, 90×, 120×). (**A**) Venn diagram of shared *de novo* SNVs among four different coverage data. Especially, low- and middle-coverage data (30× and 60×) have many non-shared *de novo* SNVs. (**B**) Comparison of the shared and specific *de novo* SNVs between 90× and 120× coverage data. The result shows that 90× coverage data is not enough to accurate *de novo* SNV calls.

The results obtained from low coverage WGS studies (10–20-fold) make it difficult to properly call heterozygous SNVs due to a larger variance of allelic mapping bias^20^. We also demonstrated that even relatively high coverage data (around 90-fold) is not efficient for proper genotyping. Specifically, we made three different depth of coverage data sets (one-fourth, half, and three-fourths) and compared them with the full data set regarding the sensitivity and specificity. Since we obtained around 120× coverage data from the parent-offspring trio after quality filtering (father 142.0×, mother 116.9×, offspring 120.3×) for the variant detection (Supplementary Table S1), for simplicity, we call each data set as ‘30×’, ‘60×’, ‘90×’, and ‘120×’, respectively. Regarding the coverage depth efficiency to the sensitivity and specificity for the detection of *de novo* SNVs, it is shown that low (30×) and middle (60×) coverage data have many specific or non-shared *de novo* SNV candidates (Fig. 5A), and it is revealed that most of the inconsistency is due to miscalling of heterozygous SNVs owing to relatively shallow depth of reads that lead to losing statistical power (Supplementary Table S7). Even for the 90× data set, nine *de novo* SNV candidates are not shared with the 120× data set, and all of the unshared ones are revealed to be false positives by Sanger sequencing validation (Fig. 5B), showing again that a relatively high coverage data set (90×) is still not enough for accurate *de novo* SNV identification. It is then that deep-sequencing coverage data for all the members are important to call variants at heterozygous sites reliably and to identify *de novo* SNVs with minimum FPs and FNs.

Another advantage of deep-sequencing is to effectively detect CNVs based on the comparison of the read depth data among the genomes of the offspring and the parents. When the offspring has an inherited hemizygous allele from its parents, its genotype should be inevitably homozygous because of the loss of one allele. Conversely, with adequate consideration on the CNVs, we can effectively identify such hemizygous deletion events if the depth of the read coverage of the father or mother, and the offspring are half of the average depth (*i.e*., loss of an allele) [see details in Fig. 2, hemizygous deletion inherited variants (HDIVs)]. Moreover, when both of the parents have two alleles, and only the offspring have lost one allele, the MIE can be assigned as the *de novo* CNVs in the offspring. We found nine such *de novo* CNVs and most of them (8/9) is less than 6 kb in size. Microarray analysis known as array CGH (comparative genomic hybridization) can detect *de novo* CNVs; however, because of the density of probes, they can mostly identify a tract of *de novo* CNVs in a stretch of several kb. Because NGS data includes read depth information for each site, CNV detection using NGS is more sensitive than that using microarrays.

A recent study which characterized *de novo* structural changes in the human genome reported that the rate of *de novo* CNVs is 0.16 per generation^28^. The rate significantly differs from our result (nine *de novo* CNVs in our study). However, they used shallow sequence depth data (14.5×), and it is therefore that their result probably contains some false negative (unidentified) *de novo* CNVs due to lack of statistical power for identification of such CNVs. Moreover, it is revealed that the longer-read sequencing technologies uncover the novel and complex structural variations in the human genome^29^, and the actual rate of *de novo* CNVs might higher than the currently reported rate (0.16 per generation).

In this study, we were also able to show the presence of other modes of *de novo* variants. For example, our analyses revealed 476 sites representing an inherited variant with no depth variation among the trio (Fig. 2 [ii]). These sites tend to be highly clustered and are mainly distributed within < 10 kb (Supplementary Figures S1, S2, S3). We annotated these variants as copy number neutral inherited variants (CNIVs) and speculated that they were generated through an allelic conversion. When an allelic conversion occurs via homologous recombination between sister chromatids, in which one of the alleles is converted to the other, that results in loss of heterozygosity. Hence, SNVs are subjected to the allelic conversion generate MIEs (Fig. 2 [ii]). However, definitive and reliable detection of such allelic conversions is very difficult because the frequency of SNVs is considerably less dense (0.0012, an average of one SNV per 833 bp of the genome) than that required to reliably detect conversion events due to the shorter mean conversion tract length of 55–290 bp^30^. Moreover, CNIVs can also arise through uniparental isodisomy (UPID), in which a single chromosome or part of a chromosome from one of the parents is inherited and duplicated via malsegregation during meiosis or post-zygotic mitosis. UPID is reportedly involved in certain human disorders, including Prader-Willi and Angelman syndromes, which are caused by malsegregation of imprinting genes on chromosome 15q, although the loss of its heterozygous tract generally extends from several hundred kb to the entire chromosome. In this study, we identified maximum loss of several kb-long heterozygous tracts; therefore, we assumed that most of the events identified in this study likely to be caused by allelic conversions. In any case, we have efficiently identified the structural dynamics of copy number alterations at the whole genome level using ultra-deep sequencing data, which is difficult through conventional cytogenetic and/or microarray analyses.

Of the 45 *de novo* candidate SNVs, we show that approximately 35 are germline *de novo* SNVs and have estimated its mutation rate as 1.48 × 10^−8^ per site per generation. The rate is approximately 23–54% greater than the human mutation rate of 0.96–1.20 × 10^−8^ per site per generation. This difference may be explained, in part, by the richness of SNVs and structural variant information for humans. Most human studies exclude the SNVs registered in the dbSNP database and residing within known segmental duplication regions. We agree with the concept of excluding the SNVs within known segmental duplication regions because of a higher probability of the NGS-derived short read mapping error. Indeed, we removed known low-complexity regions, such as LINE/SINE from the analysis. However, we believe that the exclusion of the SNVs registered in the dbSNP is not appropriate because it is known that there are many hypermutable sites of CpG in the dbSNP. In the CpG sites, we can expect that independent and recurrent mutations have occurred due to their deamination property, which converts 5-methylcytosine into thymine. In fact, Besenbacher *et al*. reported that 3.5% (18/508) of the germline *de novo* SNVs in their multiple human trio genome analysis were already present in the dbSNP and that half of the sites were located on the CpG sites^31^. They concluded that these overlaps were due to recurrent mutations, in particular on the hypermutable CpG sites. Our chimpanzee study also revealed that 29% (9/31) germline *de novo* SNVs are on CpG sites. These observations do not adequately support the exclusion of the SNVs registered in the dbSNP. Regardless, if we exclude the SNVs that are located inside the known chimpanzee segmental duplication regions^32^, two *de novo* SNVs (chr2A: 102577476, chr22: 22163245) are excluded from the list, which results in a *de novo* SNV rate of 1.45 × 10^−8^ per site per generation [43 (45–2) − 9 (0.2 FP rate)]/{1.170 × 10^9^ [(original analyzed region) − (total dbSNP sites) − (segmental duplication regions)] × 2 (per haploid)}. Since there is an order of magnitude difference of accumulated information between humans and chimpanzees regarding SNVs and structural variants, more chimpanzee variation data may narrow the gap between the *de novo* mutation rates of humans and chimpanzees.

Other cause of different mutation rates between the two species might be the difference of the germline cell cycles. For example, one cycle of spermatogonial stem cell division takes 16 and 14 days in humans and chimpanzees, respectively^33,34^, suggesting a higher number of mutation events in chimpanzees compared with humans over a given time interval (per year number of cell divisions is approximately 23 and approximately 26 in humans and chimpanzees, respectively). The difference in cell cycles may account, in part, for this discrepancy.

An additional possibility for the inconsistency of the mutation rate may come from the uncertainty of parameters used for the phylogenetic approaches, or from the inaccurate analyses of NGS studies. Phylogenetic analyses commonly incorporate genetic divergence between humans and chimpanzees (*d* = 0.012), generation time (*g* = 20), divergence time (*t* = 6 Ma), and common ancestral population size (*N*_*e*_ = 10,000) to estimate the rate as 1.88 × 10^−8^ per site per generation or 0.94 × 10^−9^ per site per year. However, if the actual divergence time of humans and chimpanzees is greater, or the average number of years per generation is >20, or if the ancestral *N*_*e*_ is greater than the assumed value of 10,000, the phylogenetic mutation rate becomes similar to that of the pedigree-based mutation rate. Indeed, if we assume that the human-chimpanzee common ancestor *N*_*e*_ is ten times higher than the assumed value (10,000) according to the theoretical study^35^, the phylogenetic mutation rate becomes 1.20 × 10^−8^ per site per generation or 0.60 × 10^−9^ per site per year^36^. Conversely, NGS analyses of pedigrees are somewhat immature because of the lack of a robust framework to identify FPs and FNs, the inability to sequence through repetitive sequences, and a bias against GC-rich DNA, suggesting that the mutation rate according to pedigree analysis represents a lower bound^37^. Interestingly, genetic studies of alternative populations that examine sequence data for genes estimated an intermediate mutation rate (1.3–1.8 × 10^−8^ per site per generation)^38,39^, suggesting the appropriate value lies within this range.

Using the data obtained from six chimpanzee offspring, the germline *de novo* SNV rate was estimated to be approximately 1.2 × 10^−8^ per site per generation (mean coverage was approximately 28×)^27^, which is consistent with the mutation rate of the human genome and is lower than the rate obtained in this study. One of the possible explanations for the difference could be the difference in the father’s age. The studies cited the above-used offspring with relatively younger fathers (mean 18.9 years; range, 14.6–23.9 years) than that of the father in this study (24 years). The effect of age may partially explain the elevated mutation rate reported in this study. Nevertheless, more data covering a wider age range (particularly the father’s) are required to define the evolutionary transition of mutation rates of hominoid genomes, and to define the effect of the ages of the parents to the overall genetic effect to the offspring.

## Methods

### The chimpanzee parent-offspring trio and animal welfare and care

The chimpanzee parent-offspring trio, the father who is called Akira [ID: 0435 in the Great Ape Information Network (GAIN), http://www.shigen.nig.ac.jp/gain/]; the mother who is called Ai [ID: 0434]; and the offspring who is Ayumu [ID: 0608], used in this study are western African chimpanzees (*Pan troglodytes verus*) reared in the Primate Research Institute, Kyoto University, Japan. The parents were wild-born and offspring were born by artificial insemination. They live in a social group with nine other chimpanzees in a semi-natural enriched outdoor compound (770 m^2^) and the two cages that were interconnected. Blood DNA samples were used for constructing genomic libraries. To minimize suffering, blood was not collected for the purpose of the present study but as part of routine health examinations. The blood DNA was extracted using the DNeasy Blood & Tissue kits (QIAGEN GmbH, Hilden, Germany). For validation of *de novo* mutation analysis, DNA samples representing a different cell lineage other than blood cells (mesoderm) were obtained from hair follicle cells (ectoderm) of the offspring. QIAamp DNA Investigator kits (QIAGEN GmbH) were used to extract hair follicle DNA from approximately 0.5 mm of the whole root of the hair.

All experiments were performed according to the Guidelines for Care and Use of Nonhuman Primates Versions 2 and 3 of the Primate Research Institute, Kyoto University (2002, 2010). The Animal Welfare and Animal Care Committee (Monkey Committee) of the Primate Research Institute approved the experiments (2010-002, 2011-063, 2012-014, 2012-124, 2013-118, 2013-175, 2014-097).

### Genome library construction and sequencing

Genomic libraries were prepared using Illumina TruSeq DNA Sample Prep kits (Illumina, Inc., CA, US) without an amplification step to produce the final products. Two types of paired-end libraries were generated using different insert fragment sizes (300 bp and 500 bp) and were sequenced using 2 × 101 cycles for each trio. All libraries were sequenced using an Illumina HiSeq. 2000 following the manufacturer’s protocols.

### Mapping reads to the chimpanzee reference sequence

Adaptor sequences and low-quality bases were removed using an in-house script before mapping (Fig. 1A; step [ii]). Low-quality sequences were defined by the averaged quality value (QV) <20 for a given base ±1 adjacent nucleotide and were marked. If a marked position was located at either the 5′- or 3′-end or both, these bases were trimmed. Finally, only high-quality paired-end (PE) reads with ≥20 nucleotides were selected. Overall, we obtained 509 Gb, 417 Gb, and 435 Gb of sequences of the father, mother, and offspring, respectively. The Burrows-Wheeler aligner (BWA; version 0.6.1)^40^ was used to align the reads, using default parameters, to the chimpanzee reference genome sequence CHIMP2.1.4 assembly from Ensembl (http://www.ensembl.org/) (Fig. 1A; step [iii]).

Alignments were converted from sequence alignment/map (SAM) format to sorted, indexed binary alignment/map (BAM) files (SAMtools; version 0.1.19)^41^, and the Picard tool (version 1.93) was used to remove duplicate reads (Fig. 1A; step [iv]). Using the sorted BAM files, we used samtools to generate genotype calls. The “mpileup” command in samtools was used to identify SNVs (http://samtools.sourceforge.net/mpileup.shtml). We used a variant call format (vcf) file for the trio, which is used to determine common and unique SNVs between members. GATK software tools^42^ (version 2.1-9) were used to improve the initial mapping results, genotype calling, and refining using the recommended parameters^20,43^ (http://www.broadinstitute.org/gatk/guide/best-practices). BAM files were realigned using the GATK IndelRealigner, and base quality scores were recalibrated using the GATK base quality recalibration tool with known variant data (common variants among the trio generated using samtools mpileup) (Fig. 1A; step [v]). The proper pair mapping results were independently selected for each read by discarding an inconsistent pair (two reads on the same chromosome with incorrect orientations or incorrect insert size) or singletons (one of the reads was unaligned). We only used unique best alignments. To do this, specific tags generated by BWA after alignment, including ×0 (number of best hits) and ×1 (number of suboptimal hits), were used to extract unique alignments (using SAM tags including ×0:i:1 and ×1:i:0) (Fig. 1A; step [vi]).

Detailed analysis pipeline and command for mapping and variant calling are shown in Supplementary Method.

### Calling SNVs and indels

The BAM files produced above were used for calling SNVs and indels using UnifiedGenotyper implemented in GATK software tools^42^ (version 2.1-9) after applying the parameters for each individual as follows: -stand_call_conf 50, -stand_emit_conf 10, and -dcov max_depth. Subsequent filtering of SNVs was performed by discarding low-quality variants according to the score calculated from UnifiedGenotyper analysis; the second most likely phred-scaled likelihoods (PL)–the most likely PL <200 for heterozygous SNVs and <100 for homozygous SNVs for reducing FPs (Fig. 1A; step [vii]).

### Filtering by read depth, allele balance, and identification of uncertain read mapped regions

To detect authentic variants and to minimize FPs, target regions with high confidence of variant calling should be defined by excluding the genomic regions according to the following filter criteria:

#### (i) Read depth

To filter out read depth outliers, the mean and standard deviation of read depth of each individual should be calculated. We then calculated the mean and standard deviation of trio read depths after setting the proper range of read depth, where lower is the minimum read depth (father 15×, mother 15×, offspring 18×) and upper is 512×, because unusual lower and higher coverage of regions (e.g., some region covering >100 K reads) confound accurate calculation of the median and standard deviation. Using the calculated mean and standard deviation, read depth ranges was set to ±3σ for each individual (Fig. 1B). This filter removed 163,077,725 bp (6.28%).

#### (ii) Mapped-read balance

Considering allelic balance read mapping, at least 10 forward and reverse reads were used to map genomic regions. This filter removed 190,025,818 bp (7.32%).

The next three filters identified uncertain read mapped regions and excluded low complexity regions as uncertain for variant calling.

#### (iii) Indels

Indel calling using NGS is highly challenging, with a high probability of obtaining FPs. The indels, which were annotated using GATK software tools (UnifiedGenotyper), and adjacent 50 bp were then excluded from target genomic regions. This filter removed 86,308,113 bp (3.33%).

#### (iv) Allelic and strand bias

Allelic and strand bias effects for variant calling have been previously mentioned^20^. We subsequently retained the variant sites that were covered by at least one read on the reference forward strand (RF), reference reverse strand (RR), alternative forward allele (AF), and alternative reverse allele (AR). For example, we retained SNVs A (20 forward reads, 12 reverse reads) and G (15 forward reads, 18 reverse reads) but discarded SNVs A (18 forward reads, 0 reverse reads) and G (19 forward reads, 22 reverse reads). All biased SNVs and adjacent 10 bp sites were excluded from the genomic target regions. This filter removed 7,455,688 bp (0.29%).

#### (v) Gaps

All variant sites located at the end of the read, with average sizes from the end of read within 10 bp, were excluded from genomic target regions and adjacent 10 bp sites were also excluded. This filter was intended to exclude uncertain variants located adjacent to relatively large contig/scaffold gaps. This filter excludes low-quality variants at the terminus of each read because the quality of both sides of a read tends to be lower. This filter removed 12,527,318 bp (0.48%).

We removed 281,326,851 bp (10.84%) using these filters and ultimately defined the target genomic regions that were shared among the trio, covering 89.16% of the chimpanzee reference genome (Tables 1 and 2).

Detailed analysis pipeline and command for filtering low-quality variant are shown in Supplementary Method.

### Identification of candidate Mendelian Inheritance Error sites (MIEs), classification of MIEs into inherited variants, and *de novo* SNVs

All the variant sites annotated using the variant calling method described above were investigated as potential *de novo* SNVs of the trio. MIEs were identified when the pattern of alleles observed in the offspring was inconsistent with the assortment of the parental alleles. Among the identified MIEs, if an allele was not present in either parent and newly emerged (mutated) in the offspring, these sites were classified as *de novo* SNVs (Fig. 2). If each allele in an offspring is present in either parent or in both, we classified the site as an inherited variant. Focusing on the depth variation among the trio, inherited variants can be classified into two different classes of variants as follows: (a) copy number neutral inherited variants (CNIVs), where no depth variation among the trio exists and (b) a hemizygous deletion inherited variants (HDIVs), in which either parent and the offspring show half read depth from the average (Fig. 2). Moreover, if depth variations occur only in the offspring, we classified these as *de novo* CNVs.

### Identification and quantification of read depth variations across the trio

To detect variations from mean depth across the trio, we used the program for detecting copy number changes using short sequence reads produced by NGS sequencer (VarScan ver2.3.5)^44^ by comparing father-offspring and mother-offspring in a pairwise manner. If there were no copy number changes (i.e., Offspring = Father = Mother), they were classified as CNIVs (Fig. 2 [ii]). If copy number changes were detected in either pairwise comparison (i.e., Offspring = Father, Offspring < Mother or Offspring < Father, Offspring = Mother), they were categorized as HDIVs (Fig. 2 [iii]). In the last category, where copy number changes were found in both pairwise comparisons (i.e., Offspring < Father, Offspring < Mother), we classified the variants as *de novo* CNVs in the offspring and found a relatively large such *de novo* CNV (11,284 bp) on chromosome 22 (Supplementary Table S4), and all sites in these regions are homozygotes (namely loss of heterozygosity or LOH).

### PCR and Sanger sequencing

The *de novo* SNV candidates were used to validate the genotype and to identify germline *de novo* SNVs using Sanger sequencing. Blood DNAs from the trio were used for Sanger validation to confirm the NGS variant calls and to estimate FPs and FNs. Moreover, DNA from mesoderm-derived hair follicles of the offspring was used to determine whether each *de novo* SNV occurred in the germline or somatic cell lineages. The variants were genotyped using PCR amplification of 2.5 ng of DNA contained KAPA2G Robust DNA polymerase (Kapa Biosystems Inc., Woburn, MA, USA) followed by Sanger sequencing using an ABI 3730 automatic genetic analyzer. The sequence reads were analyzed using the Sequencer software package and were compared to the results generated using HiSeq data.

### Data Access

All sequence reads were deposited in the DDBJ Sequence Read Archive (SRA) under accession number DRA003107. SNV information used in this study is available at http://map4.nig.ac.jp/cgi-bin/gb2/gbrowse/chimpanzee/.

## Electronic supplementary material

Supplementary Information
