# Supplementary material for: Direct estimation of de novo mutation rates in a chimpanzee parent-offspring trio by ultra-deep whole genome sequencing
Source: Sci Rep. 2017 Nov 1;7:13561. doi: 10.1038/s41598-017-13919-7 (PMC5666008; doi:10.1038/s41598-017-13919-7)
Supplement: Supplementary file 1 — Supplementary Information [file 41598_2017_13919_MOESM1_ESM.pdf]

Supplementary Information

Direct estimation of *de novo* mutation rates in a chimpanzee parent-offspring trio by ultra-deep whole genome sequencing

Shoji Tatsumoto, Yasuhiro Go, Kentaro Fukuta, Hideki Noguchi, Takashi Hayakawa, Masaki Tomonaga, Hirohisa Hirai, Tetsuro Matsuzawa, Kiyokazu Agata and Asao Fujiyama

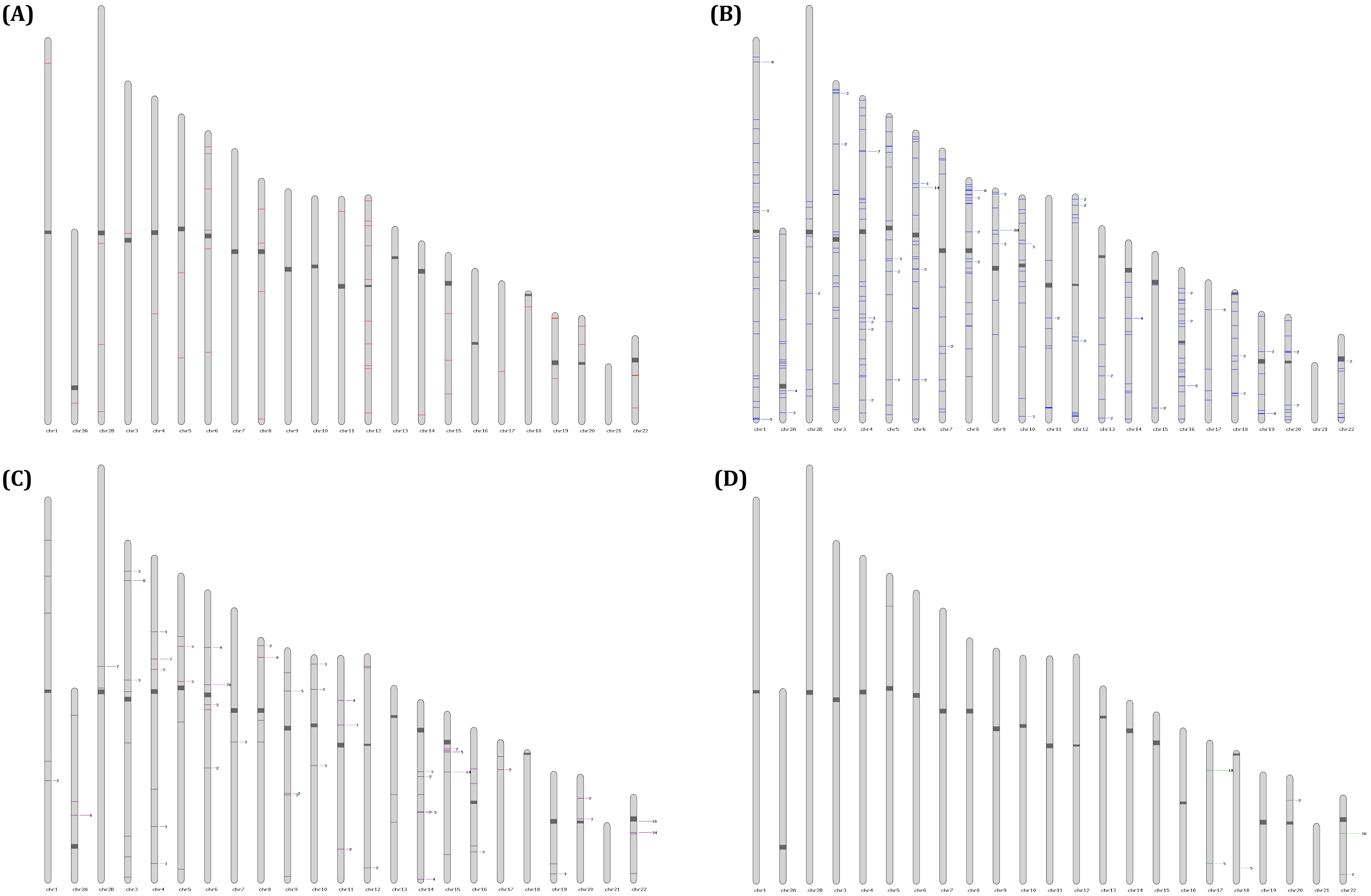

Supplementary Figure S1. A chimpanzee karyotype showing the position of the MIEs. They are classified into: (A) *de novo* SNVs, (B) copy number neutral inherited variants (CNIVs), (C) hemizygous deletion inherited variants (HDIVs), and (D) *de novo* CNVs. Multiple MIEs were closely located on the genome. Horizontal lines and the numerical numbers to the right of each chromosome indicate the positions and the number of MIEs located into the given position. The detail of the results for all MIEs appear in Supplementary Table S4.

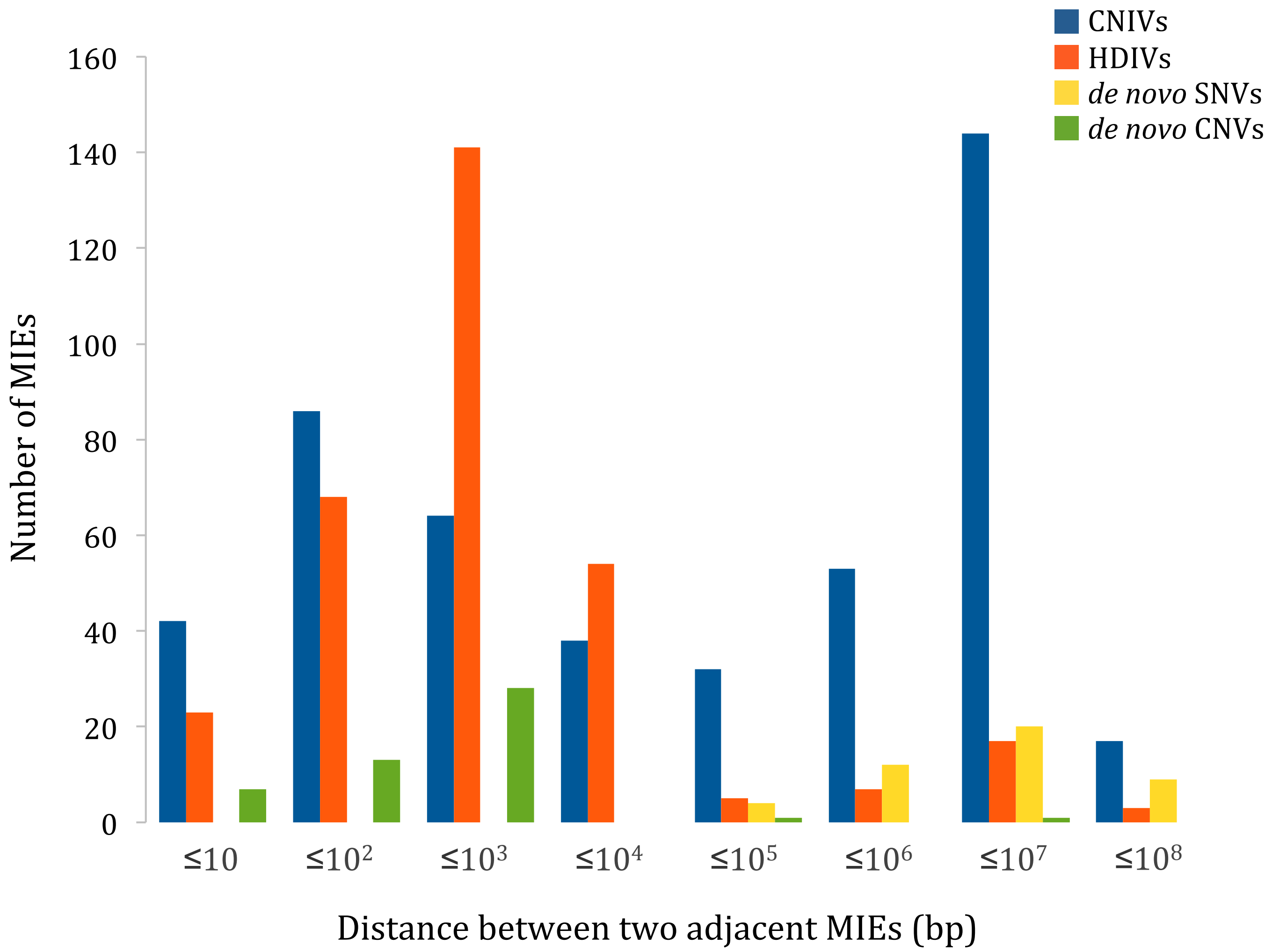

Supplementary Figure S2. Genetic distance between two adjacent CNIVs (blue), HDIVs (orange), *de novo* SNVs (yellow), and *de novo* CNVs (green). Most of the distance of two adjacent CNIVs and HDIVs significantly shorter than the one of these which are randomly distributed. However, two types of *de novo* mutations (*de novo* SNVs and *de novo* CNVs) did not form a cluster and distributed randomly.

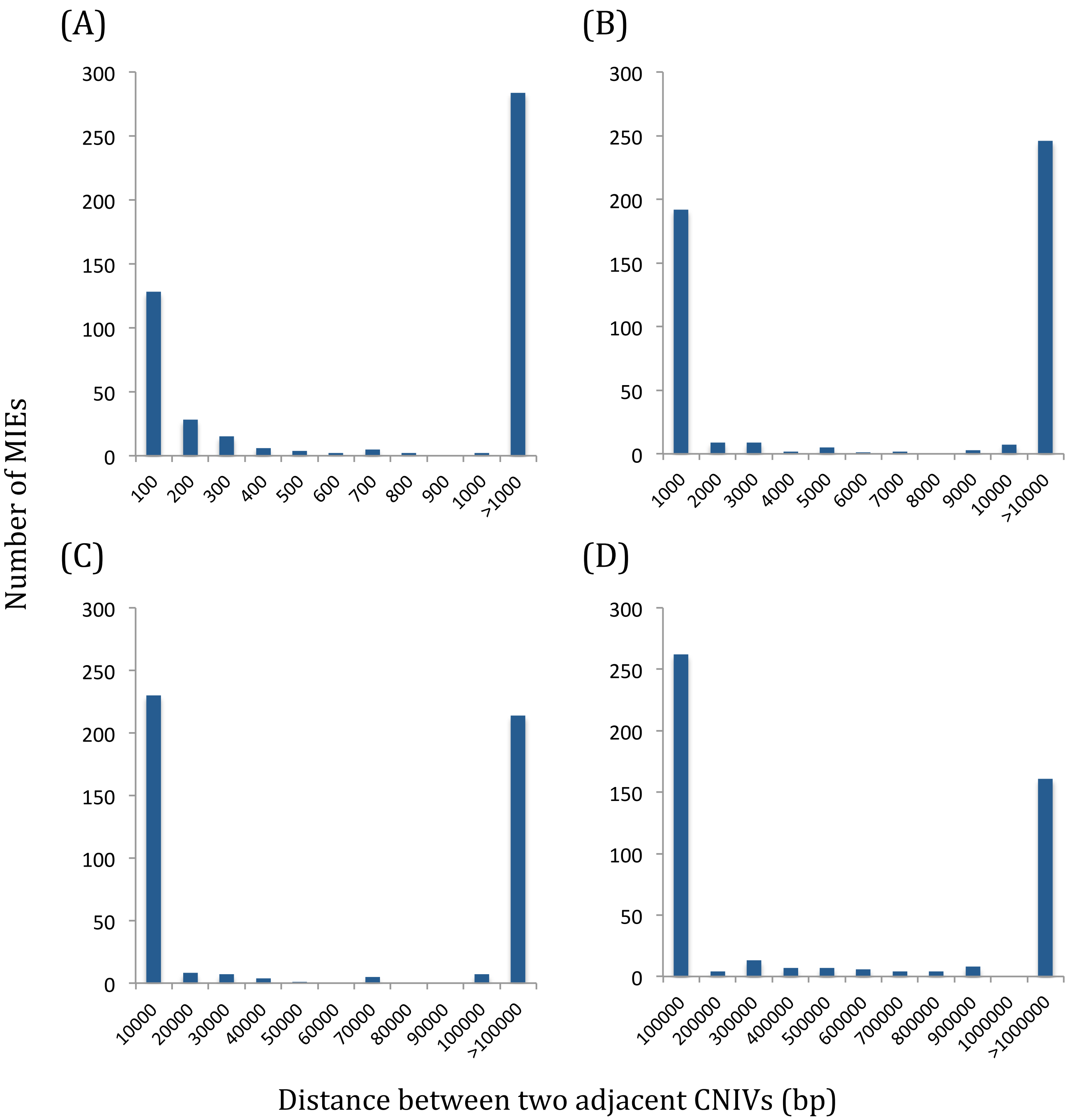

Supplementary Figure S3. Histogram of the distance between two adjacent CNIVs (bp) in units of 100s (A), 1000s (B), 10000s (C), and 100000s (D). If each CNIV is randomly distributed on the genome, the mean distance is calculated as 2.46×10^6, and 99% range of them falls between 1.75×10^4 and 1.25×10^7. However, in real datasets, most of the CNIVs (238/476) have an adjacent CNIV within 1.75×10^4. Especially, 128 of them have an adjacent CNIV within less than 100 bp.

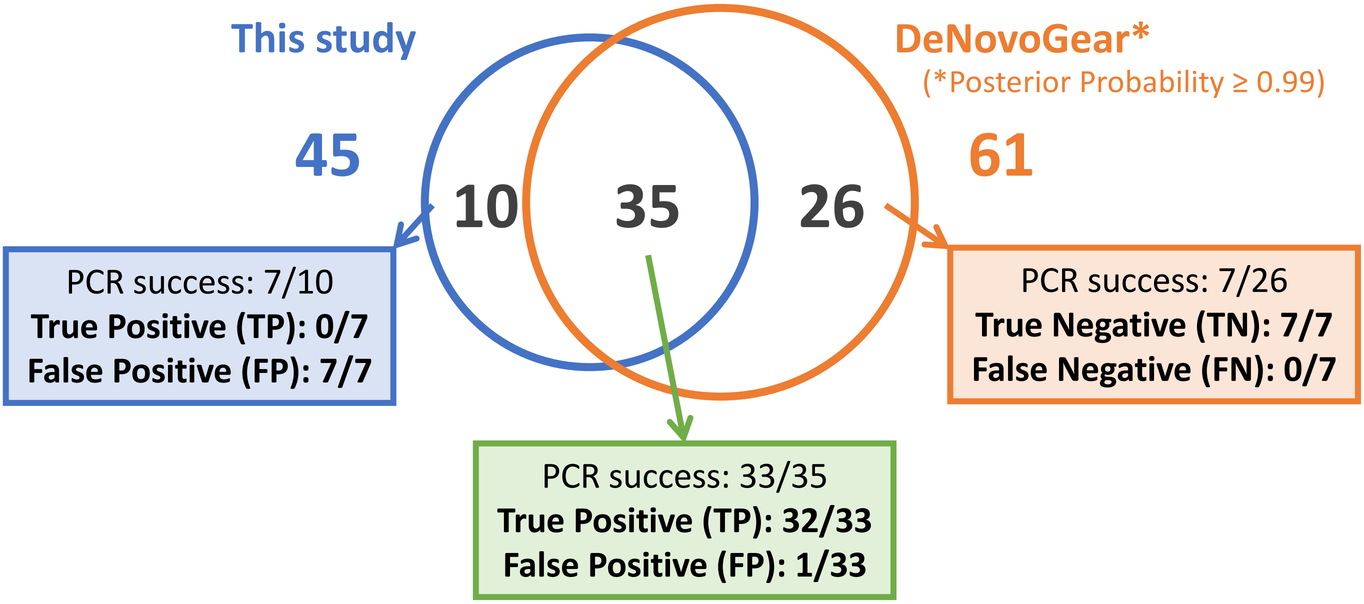

Supplementary Figure S4. Sanger sequencing validation for the two methods of NGS variant calling and identification of true positive (TP), false positive (FP), and false negative (FN).

Supplementary Table S1. Summary of whole genome deep sequencing and analyzed read coverage

Individual	Raw Data	No. Illumina Lanes used	Adapter Cut and QV Trim	Mapped	Uniq Mapped	Custom Filter	Genome covered w/o N bases (Common)
**Father "Akira"**	575 Gb (194.6x)	15	509 Gb [100%]	471 Gb [92.5%]	411 Gb [80.7%]	328 Gb (142.0x)	89.16%
**Mother "Ai"**	463 Gb (157.8X)	16	417 Gb [100%]	380 Gb [91.2%]	335 Gb [80.3%]	270 Gb (116.9x)	
**Offspring "Ayumu"**	468 Gb (158.3x)	16	435 Gb [100%]	406 Gb [93.4%]	348 Gb [80.0%]	278 Gb (120.3x)	

Supplementary Table S2. Detail of SNV variants

Individual	Father	Mother	Offspring
**depth**	27≤depth≤251	29≤depth≤199	34≤depth≤201
**Homo SNV (autosome)**	977,567	968,196	975,445
**Hetero SNV (autosome)**	1,748,513	1,767,067	1,751,084
**Total SNV (autosome)**	2,726,080	2,735,263	2,726,529
**%SNV (autosome)**	0.118	0.118	0.118
**%Heterozygosity (autosome)**	0.076	0.076	0.076
**Homo SNV (chrX)**	61,492	36,937	60,434
**Hetero SNV (chrX)**	1,419	52,367	1,299
**Total SNV (chrX)**	62,911	89,304	61,733
**%SNV (chrX)**	0.058	0.082	0.057
**%Heterozygosity (chrX)**	0.001	0.048	0.001
**Homo SNV (chrY)**	181		180
**Hetero SNV (chrY)**	324		276
**Total SNV (chrY)**	505		456
**%SNV (chrY)**	0.005		0.005
**%Heterozygosity (chrY)**	0.004		0.003
**Ti (autosome)**	1,810,503	1,818,242	1,811,915
**Tv (autosome)**	915,577	917,021	914,614
**Total Ti and Tv (autosome)**	2,726,080	2,735,263	2,726,529
**Ti/Tv (autosome)**	1.98	1.98	1.98
**Ti (chrX)**	39,589	56,751	38,847
**Tv (chrX)**	23,322	32,553	22,886
**Total Ti and Tv (chrX)**	62,911	89,304	61,733
**Ti/Tv (chrX)**	1.70	1.74	1.70
**Ti (chrY)**	328		297
**Tv (chrY)**	177		159
**Total Ti and Tv (chrY)**	505		456
**Ti/Tv (chrY)**	1.85		1.87
**[%] Genome covered w/o N bases (Common)**	89.16		
**[%] CDS covered (Common)**	93.56		

Supplementary Table S3. List of repetitive regions annotated by RepeatMasker in MIE sites

Type	counts
None	889
LINE/L1	508
SINE/Alu	196
LTR/ERV1	153
Simple_repeat	151
LTR/ERVL-MaLR	85
Satellite/telo	74
LTR/ERVL	43
Satellite/Y-chromosome	42
Low_complexity	42
SINE/MIR	41
Satellite	39
LINE/L2	34
DNA/hAT-Charlie	31
DNA/TcMar-Tigger	26
DNA/TcMar-Mariner	14
LTR/ERVK	7
DNA/hAT-Tip100	6
DNA/TcMar-Tc2	5
DNA/hAT-Blackjack	5
LINE/CR1	4
Other	3
Simple_repeat:Low_complexity	2
DNA/hAT	1
LINE/RTE-X	1
RC?/Helitron?	1
scRNA	1
snRNA	1

Supplementary Table S4. Validation and detail of MIE variants

Chr	Position	Classification	Deletion Size (bp)[a]	REF	Father	Mother	Offspring	Segmental Duplication[b]	Father	Mother	Offspring	Father (REF)	Father (ALT)	Mother (REF)	Mother (ALT)	Offspring (REF)	Offspring (ALT)
					Genotype				Hemizygous Deletion			Read Counts					
chr1	9938853	CNIVs		A	GG	AG	AA					0	38	29	20	40	0
chr1	13046491	CNIVs		C	CC	GG	GG	Yes				53	0	0	161	0	61
chr1	13046494			G	GG	AA	AA	Yes				52	0	0	157	0	58
chr1	13046538			G	GG	CC	CC	Yes				42	0	0	155	0	51
chr1	13046543			T	AA	TT	TT	Yes				0	46	161	0	56	0
chr1	13046874			G	GG	AA	AA	Yes				61	0	0	186	0	57
chr1	13046978			G	GG	TT	TT	Yes				60	0	0	190	0	57
chr1	13552700	*de novo* SNVs		C	CC	CC	CT					74	0	83	0	52	18
chr1	24236029	HDIVs	11103	T	TT	A	T			Yes	Yes	179	0	0	66	54	0
chr1	45718369	HDIVs	2298	T	T	CC	CC		Yes		Yes	86	0	0	134	0	60
chr1	47500026	CNIVs		A	CC	AA	AA	Yes				21	160	105	0	87	0
chr1	53143445	CNIVs		C	CC	TT	CC					112	0	0	51	50	0
chr1	62039711	CNIVs		G	GG	GA	AA					28	0	42	36	1	42
chr1	68040864	HDIVs	6007	G	G	GC	C		Yes		Yes	100	0	88	91	0	59
chr1	73675538	CNIVs		A	AA	TT	AA					158	0	0	77	65	1
chr1	80977580	CNIVs		G	GA	AA	GG					41	58	0	39	50	0
chr1	86009653	CNIVs		A	AG	GG	AA					37	37	2	61	62	0
chr1	97786847	CNIVs		C	CT	CC	TT					62	90	70	0	0	68
chr1	100267306	CNIVs		G	AA	GG	GG					0	80	131	0	68	0
chr1	102374760	CNIVs		C	GG	CC	CC					0	38	78	0	44	0
chr1	102421717	CNIVs		T	CC	TT	TT					0	61	149	1	82	0
chr1	102422417			C	GG	CC	CC					0	60	89	0	46	0
chr1	102422418			T	AA	TT	TT					0	61	89	0	47	0
chr1	103589755	CNIVs		T	TT	CC	CC					52	0	0	54	0	52
chr1	117864668	CNIVs		C	TT	CC	TT					0	59	68	0	0	46
chr1	119117416	CNIVs		A	GG	AA	AA					0	46	44	0	50	0
chr1	119139223	CNIVs		T	TT	GG	GG					45	0	0	43	0	45
chr1	130856616	CNIVs		A	TT	AA	TT					0	136	64	0	0	66
chr1	133487431	CNIVs		C	CC	AA	CC					111	0	0	36	40	0
chr1	142607847	CNIVs		A	AT	AA	TT					38	40	42	0	0	51
chr1	149404302	CNIVs		G	AA	GG	GG					0	59	108	0	49	0
chr1	157090230	HDIVs	1895	A	T	AA	A		Yes		Yes	0	88	121	1	54	0
chr1	168654098	HDIVs	27296	T	TG	T	G			Yes	Yes	73	65	75	0	0	61
chr1	168654957			C	CG	C	G			Yes	Yes	71	58	56	0	0	56
chr1	169235820	CNIVs		G	GG	AA	GG					132	1	0	77	68	0
chr1	176572752	CNIVs		G	GG	AA	GG					71	0	0	65	62	1
chr1	201735540	CNIVs		G	AA	GG	GG					1	68	39	0	36	0
chr1	203615668	CNIVs		C	CC	TT	CC					63	0	0	51	53	0
chr1	208542692	CNIVs		A	GG	AA	GG					0	90	67	0	0	43
chr1	217187071	CNIVs		C	CC	TT	CC					74	0	0	78	76	0
chr1	220863742	CNIVs		T	TT	AA	TT					169	0	0	65	61	0
chr1	227258970	CNIVs		T	AA	TT	TT					8	73	105	0	72	0
chr1	227625620	CNIVs		C	CC	CG	GG					27	0	14	29	0	36
chr1	227979316	CNIVs		A	AG	AA	GG					52	71	76	0	0	53
chr1	227979326			T	CC	TT	CC					0	99	74	0	0	48
chr1	227979983			A	AA	GG	AA					121	0	0	42	59	0
chr1	227979989			A	AA	GG	AA					138	0	0	44	63	0
chr1	227980021			A	AC	AA	CC					78	64	56	0	0	59
chr2A	1735565	CNIVs		C	CC	TT	TT					56	0	0	39	0	44
chr2A	14641035	HDIVs	1484	C	A	CA	C		Yes		Yes	0	48	58	46	54	0
chr2A	29718300	CNIVs		C	CC	TT	CC					100	0	0	62	62	0
chr2A	53245697	CNIVs		C	CC	AA	CC					119	0	0	61	58	0
chr2A	66415230	CNIVs		C	CC	GG	CC					138	0	0	53	69	0
chr2A	66415420	HDIVs	1130	G	GA	G	A			Yes	Yes	65	68	49	0	0	59
chr2A	67523378	CNIVs		T	TT	CC	CC					47	0	0	37	0	35
chr2A	74613815	HDIVs	55684	G	A	GG	G	Yes	Yes		Yes	0	39	90	0	38	0
chr2A	74615657			A	C	AA	A	Yes	Yes		Yes	0	53	117	0	51	0
chr2A	74617348			C	C	CT	T	Yes	Yes		Yes	60	0	40	65	0	48
chr2A	74658151			A	C	AA	A		Yes		Yes	0	58	110	0	41	0
chr2A	74658838			C	T	CC	C		Yes		Yes	0	47	96	0	44	0
chr2A	74663618			A	G	AA	A	Yes	Yes		Yes	0	46	115	0	62	1
chr2A	77334992	CNIVs		A	TT	AA	TT					0	38	54	0	0	41
chr2A	78587196	CNIVs		G	GG	AA	AA					71	0	0	109	0	87
chr2A	79309574	CNIVs		T	TT	AA	TT					64	0	0	54	52	0
chr2A	80727272	CNIVs		A	AG	GG	AA					65	53	0	45	50	0
chr2A	82401742	CNIVs		C	CT	TT	CC					40	38	0	38	44	0
chr2A	96094049	CNIVs		G	CC	GG	CC					0	38	58	0	0	48
chr2A	96094332			A	CC	AA	CC					1	57	61	0	0	64
chr2A	96094406			T	CC	TT	CC					0	40	57	0	0	71
chr2A	96094793			T	TT	CC	TT					42	0	0	39	43	0
chr2A	97593559	CNIVs		G	TT	GG	AA	Yes				39	45	53	0	0	38
chr2A	100183157	CNIVs		C	CT	CC	TT					34	37	57	0	0	34
chr2A	102577476	*de novo* SNVs		C	CT	CC	CG	Yes				116	112	190	0	73	42
chr2A	109176744	CNIVs		T	TT	TG	GG	Yes				50	0	45	30	0	40
chr2A	109211286	CNIVs		T	TT	TG	GG	Yes				56	0	53	61	0	53
chr2A	109213968			C	CT	CC	TT	Yes				24	39	37	0	0	34
chr2A	109222660			G	GG	GA	AA					65	1	53	51	66	1
chr2B	116129989	CNIVs		A	AA	TT	TT					50	0	0	66	0	36
chr2B	119150986	HDIVs	24317	G	G	GA	A		Yes		Yes	71	0	54	56	0	40
chr2B	119160940			A	G	AA	A		Yes		Yes	0	77	157	0	76	0
chr2B	119161864			C	G	CC	C		Yes		Yes	0	72	102	0	53	0
chr2B	119164280			C	C	CA	A		Yes		Yes	74	0	64	85	0	69
chr2B	119172772			G	G	GA	A		Yes		Yes	43	0	52	57	0	42
chr2B	119173645			G	G	GT	T		Yes		Yes	73	0	59	50	0	53
chr2B	119173868			G	G	GA	A		Yes		Yes	71	0	79	79	0	70
chr2B	119695215	CNIVs		T	TT	TC	CC					42	0	42	52	0	43
chr2B	126629317	CNIVs		G	AA	GG	AA					0	140	69	0	0	65
chr2B	132191841	HDIVs	5842	G	GG	A	G	Yes		Yes	Yes	146	0	0	43	48	0
chr2B	137042159	CNIVs		T	TT	GG	TT					121	0	0	74	63	0
chr2B	140770230	*de novo* SNVs		G	GG	GG	GA					138	0	111	0	73	74
chr2B	143425625	CNIVs		G	GA	AA	GG					100	98	5	85	68	0
chr2B	171392893	CNIVs		G	AA	GG	GG					0	39	75	0	44	0
chr2B	171427536	CNIVs		C	AA	CC	CC					0	80	105	0	63	0
chr2B	171444831			A	AA	AG	GG					46	0	58	58	0	52
chr2B	189849328	CNIVs		A	GG	AA	GG					0	97	83	0	0	80
chr2B	201447131	*de novo* SNVs		G	GG	GG	GC					155	0	122	0	69	65
chr2B	217456733	CNIVs		G	GG	AA	GG					75	0	0	41	37	0
chr2B	229298197	CNIVs		T	CC	TT	TT					11	99	143	0	109	0
chr2B	233245644	CNIVs		C	CC	GG	CC					51	0	0	51	53	0
chr2B	241607083	*de novo* SNVs		T	TG	TG	TC					43	78	22	43	27	70
chr3	3699758	CNIVs		G	GG	TT	TT					76	0	0	60	0	57
chr3	3928913	CNIVs		C	TT	CC	CC					0	73	131	1	54	0
chr3	5384043	CNIVs		C	CC	TT	CC					58	0	0	45	65	0
chr3	5743511	CNIVs		G	GG	AA	GG					78	0	0	75	83	0
chr3	5743577			A	AA	GG	AA					71	0	0	72	63	0
chr3	5743964			A	AA	GG	AA					68	0	0	60	80	1
chr3	16789770	HDIVs	3451	C	C	CT	T		Yes		Yes	66	0	44	55	0	61
chr3	16790087			G	G	GT	T		Yes		Yes	62	0	41	47	0	47
chr3	16790088			A	A	AG	G		Yes		Yes	62	0	42	47	0	47

chr3	22388787	HDIVs	20797	A	A	GG	G		Yes		Yes	121	0	0	150	0	63
chr3	22392162			A	A	GG	G		Yes		Yes	102	0	1	141	0	63
chr3	22396767			A	G	AA	A		Yes		Yes	0	120	166	0	77	0
chr3	22397570			G	G	AA	A		Yes		Yes	90	1	0	122	0	60
chr3	22398645			G	G	AA	A		Yes		Yes	72	0	0	135	0	51
chr3	22399517			C	C	TT	T		Yes		Yes	97	0	0	158	0	73
chr3	36475780	CNIVs		A	AA	TT	TT					41	0	0	57	0	51
chr3	36475784			T	TT	GG	GG					41	0	0	57	0	51
chr3	64225210	CNIVs		G	AA	GG	GG					0	81	125	0	75	0
chr3	66559095	CNIVs		G	GG	TT	TT					60	0	0	96	0	53
chr3	66723781	CNIVs		T	TT	CC	TT					44	0	0	41	46	0
chr3	82286207	HDIVs	7055	A	AA	G	A			Yes	Yes	170	0	0	62	50	0
chr3	82286817			A	AA	G	A			Yes	Yes	175	0	0	67	61	1
chr3	82287580			G	GG	A	G			Yes	Yes	178	0	0	66	82	0
chr3	89108735	HDIVs	3235	A	C	AC	A		Yes		Yes	0	54	47	78	62	0
chr3	89198762	CNIVs		T	GG	TT	TT					0	61	118	0	59	0
chr3	89547303	*de novo* SNVs		G	GG	GG	GA					139	0	138	0	66	57
chr3	91076996	CNIVs		G	GG	AA	GG					126	0	1	66	72	0
chr3	98969197	CNIVs		C	TT	CC	CC					6	78	121	0	69	1
chr3	101666470	CNIVs		T	TT	CC	CC					81	0	0	48	0	50
chr3	115663531	CNIVs		C	CC	GG	CC					45	0	0	47	42	0
chr3	120033520	CNIVs		A	AT	TT	AA					50	50	3	48	40	0
chr3	120043090	HDIVs	1233	T	TT	C	T			Yes	Yes	126	0	0	59	73	0
chr3	122267277	CNIVs		T	TT	AA	TT					77	1	0	48	52	0
chr3	134659588	CNIVs		A	AA	GG	AA					59	0	0	46	67	0
chr3	136400059	CNIVs		A	GG	AA	AA					0	66	61	0	63	0
chr3	141197713	CNIVs		A	AA	CC	AA					118	0	1	52	57	0
chr3	176037405	HDIVs	2713	C	CC	G	C			Yes	Yes	204	0	0	61	72	0
chr3	180637125	CNIVs		T	CC	TT	TT					0	55	65	0	55	0
chr3	188314565	HDIVs	9974	C	CC	A	C			Yes	Yes	146	0	0	46	49	0
chr3	194832443	CNIVs		A	AG	AA	GG					45	39	31	0	0	40
chr3	196175543	CNIVs		A	CC	AA	CC					0	113	49	0	0	51
chr3	200502333	HDIVs	3373	G	A	GA	G			Yes	Yes	0	95	68	70	66	1
chr4	915001	CNIVs		G	GA	AA	GG					68	60	2	39	37	0
chr4	5513515	CNIVs		G	TT	GG	TT					0	107	56	0	0	49
chr4	10415102	CNIVs		A	GG	AA	GG					0	46	45	0	0	43
chr4	18655757	CNIVs		C	TT	CC	CC					0	54	105	0	64	0
chr4	31385252	CNIVs		T	TT	TC	CC					86	0	62	63	5	68
chr4	31701339	CNIVs		G	AA	GG	GG					0	65	51	0	46	0
chr4	31701496			C	AA	CC	CC					0	74	47	0	77	0
chr4	31701813			T	TT	CC	CC					71	1	0	62	0	71
chr4	31701885			C	TT	CC	CC					0	75	59	0	88	0
chr4	31701921			T	CC	TT	TT					0	71	63	0	82	0
chr4	31701992			C	CC	TT	TT					87	0	0	53	0	81
chr4	31704272			T	TT	CC	CC					79	0	0	58	0	56
chr4	44154342	HDIVs	70716	A	AG	G	A			Yes	Yes	67	63	0	70	53	0
chr4	44168066			G	GA	G	A			Yes	Yes	106	123	64	0	0	85
chr4	44169893			G	GC	G	C			Yes	Yes	102	94	65	0	0	84
chr4	59944470	CNIVs		A	GG	AA	GG					0	107	61	0	2	62
chr4	60624101	HDIVs	12316	A	A	AG	G		Yes		Yes	67	0	58	71	0	56
chr4	60624571			G	G	GC	C		Yes		Yes	78	0	76	79	0	91
chr4	60624651			G	G	GT	T		Yes		Yes	88	0	72	67	0	83
chr4	60627000			C	C	CA	A		Yes		Yes	43	0	55	63	0	49
chr4	60627258			A	A	AG	G		Yes		Yes	93	0	65	68	0	75
chr4	60627462			A	C	AA	A		Yes		Yes	0	75	123	0	45	0
chr4	60635230			C	C	CG	G		Yes		Yes	102	0	75	79	0	74
chr4	60807232	CNIVs		G	AA	GG	AA					0	66	43	0	0	64
chr4	62691574	CNIVs		T	TT	CC	TT					84	0	0	66	69	0
chr4	66301866	CNIVs		G	GT	GG	TT					69	48	71	0	0	57
chr4	66715306	HDIVs	1143	C	CT	T	C			Yes	Yes	87	67	0	71	69	0
chr4	66715488			G	GT	T	G			Yes	Yes	87	90	0	67	58	0
chr4	72621247	CNIVs		A	AC	AA	CC					61	32	60	0	0	37
chr4	97138958	CNIVs		G	AA	GG	GG					0	49	83	0	37	0
chr4	110108763	CNIVs		T	TT	CC	CC					86	0	0	50	0	67
chr4	113024038	CNIVs		T	CC	TC	TT					0	46	32	43	41	0
chr4	119053096	CNIVs		C	CC	CG	GG					55	0	58	52	0	37
chr4	121834131	CNIVs		T	TT	TC	CC	Yes				30	0	19	23	0	36
chr4	128850953	*de novo* SNVs		C	CC	CC	CT					184	0	141	0	82	90
chr4	129578642	CNIVs		G	AA	GG	GG					0	41	106	0	43	0
chr4	132065395	CNIVs		G	GA	AA	GG					80	79	0	77	81	0
chr4	132065461			C	CA	CC	AA					78	68	49	0	0	68
chr4	132065517			C	CT	TT	CC					81	87	0	60	69	0
chr4	134337505	CNIVs		A	AG	AA	GG	Yes				62	52	69	0	0	65
chr4	134350407			C	CT	CC	TT	Yes				87	51	71	1	0	36
chr4	138756826	HDIVs	1301	A	AG	A	G			Yes	Yes	75	65	73	0	0	94
chr4	138757181	CNIVs		G	AA	GG	AA					0	214	79	0	0	57
chr4	138757386			C	AA	CC	AA					0	193	64	0	0	78
chr4	145292980	CNIVs		T	TT	CC	TT					116	0	0	57	64	1
chr4	156641492	CNIVs		A	TT	AA	AA					0	58	99	0	52	0
chr4	160147723	CNIVs		T	TT	TC	CC					93	0	68	69	0	80
chr4	161182248	HDIVs	4178	C	CG	G	C			Yes	Yes	89	107	0	75	70	0
chr4	161184483			G	AA	G	A			Yes	Yes	1	151	68	0	0	56
chr4	161184773			C	CA	A	C			Yes	Yes	78	83	0	82	74	0
chr4	166551580	CNIVs		C	CG	CC	GG					89	65	63	0	4	69
chr4	167438779	CNIVs		T	TT	TG	GG					96	0	72	55	6	73
chr4	181389000	CNIVs		T	GG	TT	GG					0	76	66	0	0	60
chr4	181389104			T	CC	TT	CC					0	83	70	0	0	90
chr4	183310289	HDIVs	1310	A	G	AA	A		Yes		Yes	0	72	117	0	59	0
chr4	183310358			C	T	CC	C		Yes		Yes	0	73	115	0	40	1
chr4	183310584			C	T	CC	C		Yes		Yes	0	78	109	0	54	0
chr4	189195877	CNIVs		A	AA	GG	AA					95	0	0	48	45	0
chr5	385532	CNIVs		G	CC	GG	GG					0	46	33	0	55	0
chr5	9107802	CNIVs		T	CC	TT	TT					2	71	129	0	59	1
chr5	17731608	*de novo* CNVs	2431	A	GG	AA	A	Yes			Yes	0	106	123	0	53	0
chr5	17794534	CNIVs		T	CC	TT	TT	Yes				0	47	100	0	47	0
chr5	18057974	CNIVs		C	CT	CC	TT					43	52	57	0	0	47
chr5	21505499	CNIVs		A	AG	AA	GG					45	51	48	0	0	37
chr5	30462168	CNIVs		C	CC	AA	CC					184	1	0	63	62	0
chr5	36278013	HDIVs	18852	C	CC	T	C			Yes	Yes	153	0	0	70	71	0
chr5	42232861	HDIVs	7320	T	TT	G	T			Yes	Yes	151	0	0	54	44	1
chr5	42234206			A	AA	G	A			Yes	Yes	131	1	0	40	49	0
chr5	42234714			T	TT	C	T			Yes	Yes	118	0	0	56	68	0
chr5	62722015	CNIVs		T	TT	TA	AA					83	0	68	67	0	59
chr5	63234311	HDIVs	2698	A	G	AA	A		Yes		Yes	0	74	123	0	59	0
chr5	63234582			G	T	GG	G		Yes		Yes	0	96	143	1	74	0
chr5	63234933			C	A	CC	C		Yes		Yes	0	91	132	0	67	0
chr5	74280729	CNIVs		A	AA	AG	GG					80	0	74	73	5	63
chr5	85555520	CNIVs		G	GG	CC	GG					121	0	0	51	54	0
chr5	85555928			C	CC	GG	CC					120	0	0	50	54	0
chr5	85559232			C	CC	AA	CC					102	0	0	39	35	0
chr5	86858477	CNIVs		C	TT	CC	TT					1	78	85	0	1	49
chr5	87567306	HDIVs	1014	C	C	CG	G	Yes	Yes		Yes	98	0	52	62	0	56
chr5	93162864	[illegible]		C	CC	TT	TT					37	0	0	80	0	42

chr5	93167182	CNIVs		T	CC	TT	TT					0	48	90	0	36	1
chr5	93480078	*de novo* SNVs		A	AA	AA	AG					211	0	162	0	77	80
chr5	107110110	CNIVs		G	GG	TT	GG					128	0	1	43	69	0
chr5	123482877	CNIVs		A	AA	AG	GG					73	0	47	59	0	53
chr5	144330507	*de novo* SNVs		G	GG	GG	GT					114	0	119	0	50	67
chr5	158301335	CNIVs		C	CT	CC	TT					55	66	69	0	0	55
chr5	158301352			C	CT	TT	CC					68	50	0	61	49	0
chr5	173549046	CNIVs		A	AA	GG	AA					141	0	1	59	78	0
chr5	175939473	HDIVs	3783	C	CA	C	A			Yes	Yes	69	51	44	0	0	66
chr5	179381161	CNIVs		T	TT	CC	TT					120	1	0	38	44	1
chr6	2107625	CNIVs		T	AA	TT	TT					0	47	75	0	38	0
chr6	3682135	CNIVs		G	AA	GG	GG					8	100	81	0	68	0
chr6	5267525	CNIVs		C	CC	TT	TT					82	0	0	51	0	79
chr6	7711997	*de novo* SNVs		A	AG	AA	AT					42	37	68	0	37	34
chr6	12022852	*de novo* SNVs		G	GG	GG	GT					79	0	72	0	48	15
chr6	14985395	CNIVs		C	CC	TT	TT					90	1	0	120	0	47
chr6	30308708	CNIVs		G	GC	GG	CC					37	38	32	0	0	36
chr6	30309342			C	TT	CC	TT					0	137	53	0	0	37
chr6	30310462			A	GG	AA	GG					0	126	58	0	0	46
chr6	32800903	CNIVs		A	AA	GG	AA					88	0	0	61	84	0
chr6	32801130			C	CC	GG	CC					83	0	0	48	60	0
chr6	32801349			A	AA	GG	AA					81	0	0	56	74	0
chr6	32801350			C	CC	AA	CC					81	0	0	57	73	0
chr6	32801472			A	AA	GG	AA					96	0	0	69	65	0
chr6	32824382	CNIVs		G	GG	AA	GG					90	0	0	66	68	0
chr6	32824700			A	AA	GG	AA					97	0	0	68	85	0
chr6	32825737			T	TT	GG	TT					85	0	0	73	56	0
chr6	32829298			T	TT	CC	TT					85	0	0	79	72	0
chr6	32829819			C	CC	TT	CC					89	0	0	63	54	0
chr6	32833990	HDIVs	7457	G	GG	A	G			Yes	Yes	99	0	0	52	57	0
chr6	32836738			G	GG	A	G			Yes	Yes	82	0	0	52	50	0
chr6	32836991			C	CC	A	C			Yes	Yes	122	0	0	75	46	0
chr6	32838493			A	AA	G	A			Yes	Yes	105	0	0	59	76	0
chr6	32844329	CNIVs		A	AA	GG	AA					84	0	0	55	63	0
chr6	32846817			T	TT	CC	TT					101	0	0	75	91	0
chr6	32849498			A	AA	GG	AA					93	0	0	58	72	0
chr6	32882251	CNIVs		G	GG	AA	GG					82	0	0	60	68	0
chr6	33261071	*de novo* SNVs		T	TT	TT	TA					166	1	61	0	49	18
chr6	49330256	CNIVs		A	AA	GG	AA					174	0	0	68	74	0
chr6	55271096	HDIVs	76491	G	GG	A	G			Yes	Yes	156	0	0	61	60	0
chr6	55271394			T	AA	T	A			Yes	Yes	0	168	68	0	0	81
chr6	55272836			A	AA	G	A			Yes	Yes	155	1	0	70	68	0
chr6	55273719			G	GG	T	G			Yes	Yes	151	0	0	71	73	0
chr6	55275069			A	AA	G	A			Yes	Yes	111	0	0	72	53	0
chr6	55275797			G	AA	G	A			Yes	Yes	0	141	69	0	0	75
chr6	55276169			A	AA	G	A			Yes	Yes	110	0	0	88	69	0
chr6	55276203			C	TT	C	T			Yes	Yes	0	114	84	0	0	64
chr6	55276232			T	CC	T	C			Yes	Yes	0	131	66	1	0	60
chr6	55277964			A	TT	A	T			Yes	Yes	0	185	73	0	0	71
chr6	55278513			G	TT	G	T			Yes	Yes	0	158	70	0	0	81
chr6	55283702			A	GG	A	G			Yes	Yes	0	161	80	0	0	76
chr6	55284070			G	GG	C	G			Yes	Yes	172	0	0	72	64	0
chr6	55284152			T	TT	C	T			Yes	Yes	188	0	0	72	89	0
chr6	55285251			C	TT	C	T			Yes	Yes	1	138	71	0	0	55
chr6	55285475			A	CC	A	C			Yes	Yes	0	156	61	0	0	73
chr6	55288614			C	GG	C	G			Yes	Yes	0	83	87	0	0	43
chr6	55288655			G	AA	G	A			Yes	Yes	1	126	76	0	0	61
chr6	55292760			A	AA	G	A			Yes	Yes	193	0	0	64	71	0
chr6	55292832			T	TT	C	T			Yes	Yes	189	0	0	77	79	0
chr6	55293288			A	GG	A	G			Yes	Yes	0	145	72	0	0	68
chr6	55293342			G	AA	G	A			Yes	Yes	0	156	67	0	0	66
chr6	55293562			A	CC	A	C			Yes	Yes	1	186	74	1	0	64
chr6	55293665			C	TT	C	T			Yes	Yes	0	170	76	0	0	87
chr6	55293943			G	GG	A	G			Yes	Yes	145	0	0	49	64	0
chr6	55293955			G	GG	C	G			Yes	Yes	160	0	0	49	68	0
chr6	55294911			C	CC	A	C			Yes	Yes	100	0	0	44	57	0
chr6	55296511			A	GG	A	G			Yes	Yes	0	132	65	0	0	63
chr6	55296715			C	TT	C	T			Yes	Yes	0	141	65	0	0	76
chr6	55297102			A	GG	A	G			Yes	Yes	0	102	74	0	0	66
chr6	55297647			T	TT	C	T			Yes	Yes	182	1	0	84	74	0
chr6	55298022			T	TT	C	T			Yes	Yes	146	0	0	67	79	0
chr6	55298227			A	AA	T	A			Yes	Yes	137	0	0	54	50	0
chr6	55299973			G	AA	G	A			Yes	Yes	0	110	54	0	0	48
chr6	55300697			T	TT	G	T			Yes	Yes	169	0	0	57	58	0
chr6	55301064			A	GG	A	G			Yes	Yes	0	128	48	0	0	70
chr6	55301452			G	TT	G	T			Yes	Yes	0	121	40	0	0	59
chr6	55301663			A	GG	A	G			Yes	Yes	0	119	60	0	0	51
chr6	55301899			G	TT	G	T			Yes	Yes	1	141	76	0	0	54
chr6	55302503			G	GG	A	G			Yes	Yes	126	0	0	61	70	0
chr6	55302584			A	AA	G	A			Yes	Yes	130	0	0	72	53	0
chr6	55302614			C	CC	T	C			Yes	Yes	132	0	0	73	56	0
chr6	55302802			T	CC	T	C			Yes	Yes	0	140	80	0	0	68
chr6	55303178			C	CC	T	C			Yes	Yes	139	0	0	83	69	0
chr6	55304016			G	GG	C	G			Yes	Yes	139	0	0	66	78	0
chr6	55308519			G	GG	A	G			Yes	Yes	134	0	0	81	86	0
chr6	55308577			T	TT	C	T			Yes	Yes	136	0	0	60	80	0
chr6	55308727			A	GG	A	G			Yes	Yes	0	151	78	0	0	56
chr6	55308984			T	CC	T	C			Yes	Yes	0	128	77	0	0	55
chr6	55309145			C	TT	C	T			Yes	Yes	0	157	88	0	0	74
chr6	55309198			G	AA	G	A			Yes	Yes	0	136	85	0	0	69
chr6	55310943			T	TT	C	T			Yes	Yes	138	0	0	59	63	0
chr6	55311115			T	CC	T	C			Yes	Yes	0	152	54	0	0	64
chr6	55311471			A	GG	A	G			Yes	Yes	0	134	52	0	0	53
chr6	55314239			A	AA	G	A			Yes	Yes	193	0	0	71	90	0
chr6	55314364			A	AA	T	A			Yes	Yes	125	0	0	43	41	0
chr6	55314818			A	TT	A	T			Yes	Yes	0	127	51	0	0	54
chr6	55315807			C	CC	T	C			Yes	Yes	191	0	0	76	70	0
chr6	55315841			A	AA	T	A			Yes	Yes	169	0	0	77	75	0
chr6	55315863			G	GG	A	G			Yes	Yes	160	0	0	71	80	0
chr6	55324245			G	AA	G	A			Yes	Yes	0	163	46	0	0	72
chr6	55328576			C	CC	G	C			Yes	Yes	202	0	0	75	89	0
chr6	55328653			A	AA	G	A			Yes	Yes	171	0	0	67	73	0
chr6	55330465			A	TT	A	T			Yes	Yes	0	186	76	0	0	53
chr6	55333304			G	TT	G	T			Yes	Yes	0	170	71	1	0	62
chr6	55333726			G	TT	G	T			Yes	Yes	0	168	67	0	0	81
chr6	55333747			T	CC	T	C			Yes	Yes	0	155	70	0	0	75
chr6	55335667			T	AA	T	A			Yes	Yes	0	169	69	0	0	71
chr6	55336670			A	GG	A	G			Yes	Yes	0	118	59	0	0	70
chr6	55336704			G	AA	G	A			Yes	Yes	0	130	76	0	0	53
chr6	55337433			T	TT	C	T			Yes	Yes	153	0	1	72	84	0
chr6	55337967			C	TT	C	T			Yes	Yes	0	142	70	0	0	74
chr6	55339412			T	TC	T	C			Yes	Yes	90	85	86	0	0	60
chr6	55339628			G	GG	A	G			Yes	Yes	191	0	0	67	66	0

chr6	55339854			T	TT	C	T			Yes	Yes	160	0	0	51	63	0
chr6	55342281			T	TT	A	T			Yes	Yes	199	0	0	61	64	0
chr6	57788180	*de novo* SNVs		C	CC	CC	CT					135	0	103	0	72	43
chr6	64953362	CNIVs		C	CT	CC	TT	Yes				67	64	57	0	0	39
chr6	65018254	CNIVs		T	TA	AA	TT	Yes				144	74	0	79	74	0
chr6	67108845	HDIVs	8348	C	T	CT	C		Yes		Yes	0	76	66	51	65	0
chr6	67108851			T	A	TA	T		Yes		Yes	0	70	71	51	70	0
chr6	67134675	CNIVs		C	CC	GG	CC					83	0	0	59	59	0
chr6	68971246	*de novo* SNVs		G	GG	GG	GT					142	1	150	0	70	69
chr6	70254931	HDIVs	8890	C	CC	T	T			Yes	Yes	96	0	0	60	0	57
chr6	74330902	CNIVs		C	CC	CA	AA					70	0	56	52	0	69
chr6	75690960	CNIVs		C	CT	CC	TT					72	88	59	0	0	58
chr6	81685824	CNIVs		T	TT	AA	TT					69	0	0	67	66	0
chr6	82019197	CNIVs		T	CC	TT	CC					0	82	66	0	0	72
chr6	82019454			A	AA	GG	AA					61	0	0	68	58	0
chr6	89405944	CNIVs		A	GG	AA	AA					0	53	66	0	51	0
chr6	105105061	CNIVs		T	TT	GG	TT					173	0	0	35	68	0
chr6	105107872	HDIVs	1111	G	CC	G	C			Yes	Yes	0	153	75	0	0	62
chr6	105107875			C	TT	C	T			Yes	Yes	0	159	76	0	0	65
chr6	105629971	CNIVs		T	CC	TT	CC					0	88	51	1	0	53
chr6	130931815	*de novo* SNVs		C	CC	CC	CG					145	0	134	0	73	65
chr6	148351437	CNIVs		C	AA	CC	CC	Yes				0	81	159	0	55	0
chr6	148352357			A	GG	AG	AA	Yes				0	45	37	37	66	0
chr6	172080259	CNIVs		A	GG	AA	AA					0	38	114	1	97	1
chr7	4597292	CNIVs		C	CC	GG	CC					125	0	0	44	48	0
chr7	4620395	CNIVs		G	GT	GG	TT					48	42	32	0	1	41
chr7	5481343	CNIVs		C	AA	CA	CC	Yes				0	71	47	59	56	0
chr7	13827603	CNIVs		G	GG	AA	GG					80	0	2	63	83	1
chr7	78800059	HDIVs	3561	A	GG	A	G			Yes	Yes	0	149	57	0	0	58
chr7	78800163			G	GT	G	T			Yes	Yes	66	82	73	0	0	64
chr7	78801537			C	CT	T	C			Yes	Yes	63	76	0	54	69	0
chr7	81852800	CNIVs		A	AA	AG	GG					100	0	84	65	9	87
chr7	99786867	CNIVs		T	AA	TA	TT					0	61	73	79	66	0
chr7	117449629	CNIVs		T	TT	CC	TT					148	0	0	61	75	0
chr7	117449832			T	TA	TT	AA					57	63	55	0	0	52
chr7	121780992	CNIVs		C	CC	TT	TT					78	0	0	63	1	65
chr7	137572274	CNIVs		T	CC	TC	TT					0	60	56	36	58	0
chr7	144426331	CNIVs		G	GG	AA	GG					114	0	0	55	52	0
chr7	155240921	CNIVs		G	GG	AA	AA	Yes				51	0	0	36	0	41
chr7	156994262	CNIVs		C	CC	TT	CC					111	0	0	54	48	0
chr8	2404605	CNIVs		A	GG	AA	AA					0	47	44	0	43	0
chr8	3220745	HDIVs	1580	C	TT	C	T			Yes	Yes	0	192	53	0	0	47
chr8	3220767			C	CC	G	C			Yes	Yes	189	0	0	57	56	0
chr8	3625891	CNIVs		T	TT	AA	TT					67	0	1	53	64	0
chr8	5222266	CNIVs		C	TT	CC	CC					3	54	60	0	53	0
chr8	5889647	CNIVs		T	TT	AA	TT					88	0	0	67	76	0
chr8	5918321	CNIVs		C	CC	TT	CC					96	1	1	71	60	0
chr8	5927514			C	CC	TT	CC					73	0	0	75	64	0
chr8	5929189			C	CC	TT	CC					72	0	0	58	66	0
chr8	5935367			T	TT	CC	TT					90	0	0	61	51	0
chr8	5935522			T	CC	TT	CC					0	55	52	0	0	62
chr8	5936822			T	TT	CC	TT					56	0	0	68	67	0
chr8	8289369	CNIVs		T	TC	TT	CC	Yes				62	51	32	0	0	44
chr8	8536286	CNIVs		T	CC	TT	TT	Yes				0	44	74	0	37	1
chr8	10160328	HDIVs	2507	C	C	CG	G		Yes		Yes	100	0	72	52	0	61
chr8	10160369			C	C	CT	T		Yes		Yes	95	0	77	58	0	62
chr8	10160667			T	T	TC	C		Yes		Yes	101	0	74	59	0	73
chr8	10160679			T	T	TC	C		Yes		Yes	101	0	71	60	0	77
chr8	10160700			C	C	CT	T		Yes		Yes	98	0	78	59	0	73
chr8	10160745			G	G	GT	T		Yes		Yes	86	0	65	63	0	65
chr8	10160792			G	G	GA	A		Yes		Yes	67	0	63	62	0	51
chr8	10160818			G	G	GC	C		Yes		Yes	59	0	56	58	0	43
chr8	10325007	CNIVs		A	AG	AA	GG					95	89	86	0	0	75
chr8	10325205			C	CT	CC	TT					96	91	75	0	0	75
chr8	11766474	CNIVs		T	TT	CC	TT					156	0	0	65	57	0
chr8	13660925	CNIVs		G	GA	AA	GG					90	77	0	64	50	0
chr8	13759037	CNIVs		G	GG	AA	GG					137	0	0	55	46	0
chr8	16568611	*de novo* SNVs		G	GG	GG	GA					166	0	131	0	69	67
chr8	30772868	CNIVs		C	CC	TT	TT					43	0	0	99	0	35
chr8	30772876			C	CC	TT	TT					31	0	0	87	0	34
chr8	37041261	*de novo* SNVs		G	GG	GG	GA					162	0	127	0	63	56
chr8	46980386	CNIVs		G	AA	GG	AA					0	97	61	0	0	49
chr8	46989890	CNIVs		A	AG	AA	GG					70	62	63	0	0	56
chr8	48044972	HDIVs	4826	C	TT	C	T			Yes	Yes	1	190	71	0	0	63
chr8	48721078	CNIVs		A	AA	TT	AA					71	0	0	55	50	0
chr8	48730530			A	AA	GG	AA					46	0	0	51	58	0
chr8	52639308	CNIVs		C	CC	TT	TT					42	0	0	35	0	41
chr8	55035452	CNIVs		T	TT	AA	AA					64	0	0	66	0	34
chr8	55893201	CNIVs		A	GG	AA	GG					0	56	36	0	0	43
chr8	60948142	HDIVs	3086	A	AA	G	A			Yes	Yes	108	0	0	57	72	0
chr8	65944369	*de novo* SNVs		C	CC	CC	CT					143	0	128	0	75	73
chr8	73385797	CNIVs		C	TT	CC	CC					0	35	36	0	40	0
chr8	80158806	CNIVs		C	CC	CT	TT					64	0	64	50	0	37
chr8	87282895	CNIVs		G	GG	AA	AA					39	0	0	34	0	44
chr8	87519862	CNIVs		C	CC	TT	TT					89	1	0	158	2	90
chr8	101236547	CNIVs		G	TT	GG	GG					0	84	112	0	68	0
chr8	117887395	CNIVs		A	GG	AA	GG					0	44	50	0	0	49
chr8	142414336	*de novo* SNVs		G	GG	GG	GA					172	0	118	0	60	63
chr9	756031	CNIVs		G	AA	GG	GG	Yes				0	35	76	0	47	0
chr9	1816010	CNIVs		G	GG	TT	GG					169	0	5	60	66	0
chr9	1816012			G	GG	TT	GG					166	0	5	62	67	0
chr9	10276672	CNIVs		C	CG	CC	GG					58	30	70	0	0	39
chr9	13163526	HDIVs	2126	T	T	TA	A		Yes		Yes	75	0	72	57	0	68
chr9	23656425			T	AA	TT	AA					0	50	78	0	0	49
chr9	23656579			C	AA	CC	AA					0	36	65	0	0	35
chr9	23656649			G	AA	GG	AA					0	54	81	0	0	63
chr9	23656656			T	CC	TT	CC					0	56	77	0	0	60
chr9	23657601			T	AA	TT	AA					0	70	61	0	0	67
chr9	23657625			C	TT	CC	TT					0	62	68	0	0	55
chr9	23657849			G	TT	GG	TT					0	81	83	0	0	47
chr9	23657870			G	CC	GG	CC					0	83	85	0	0	48
chr9	23657924			T	CC	TT	CC					0	54	60	0	0	54
chr9	23657932			C	TT	CC	TT					0	57	57	0	0	54
chr9	23657945			T	CC	TT	CC					0	59	54	0	0	57
chr9	23659352			T	CC	TT	CC					0	54	70	0	0	71
chr9	23659399			T	CC	TT	CC					0	66	67	0	0	75
chr9	23659948			T	CC	TT	CC					0	70	56	0	0	76
chr9	23659982			C	TT	CC	TT					0	59	45	0	0	63
chr9	23659993			A	GG	AA	GG					0	57	49	0	1	60
chr9	23660194			A	GG	AA	GG					0	75	65	0	0	81
chr9	23660334			C	TT	CC	TT					0	79	56	0	0	87
chr9	23660379			T	CC	TT	CC					0	80	61	0	1	78
chr9	23660413			T	CC	TT	CC					1	91	65	0	0	76

chr9	23660432	CNIVs		A	CC	AA	CC					0	84	65	0	0	85
chr9	23660506			T	GG	TT	GG					0	61	59	0	0	75
chr9	23660530			C	TT	CC	TT					0	72	47	0	0	74
chr9	23660790			C	AA	CC	AA					0	107	66	0	0	62
chr9	23661269			C	CC	TT	CC					97	0	0	62	71	0
chr9	23661394			T	GG	TT	GG					0	79	70	1	0	70
chr9	23661996			C	TT	CC	TT					0	69	64	0	0	63
chr9	23662055			C	TT	CC	TT					0	70	65	0	0	64
chr9	23662088			C	TT	CC	TT					0	66	77	0	0	64
chr9	23662111			C	TT	CC	TT					0	54	79	0	0	68
chr9	23662277			A	GG	AA	GG					0	45	85	0	0	52
chr9	23662293			C	TT	CC	TT					0	48	92	0	0	51
chr9	23662305			G	TT	GG	TT					0	49	95	0	0	52
chr9	23662363			A	TT	AA	TT					0	48	76	0	0	40
chr9	23662434			C	TT	CC	TT					0	71	80	0	0	67
chr9	23662463			C	TT	CC	TT					0	75	86	0	0	63
chr9	23663239			G	AA	GG	AA					0	71	62	0	0	70
chr9	23664727			G	AA	GG	AA					0	84	65	0	0	80
chr9	23664812			G	TT	GG	TT					0	60	52	0	0	67
chr9	23664946			T	AA	TT	AA					0	75	62	0	0	48
chr9	23665130			C	TT	CC	TT					0	88	71	0	0	73
chr9	23665227			C	TT	CC	TT					0	57	81	0	0	73
chr9	23665482			A	GG	AA	GG					0	75	77	0	0	68
chr9	23665540			C	AA	CC	AA					0	74	65	0	0	63
chr9	23665560			A	TT	AA	TT					0	79	72	0	0	68
chr9	23665663			G	AA	GG	AA					0	54	63	0	0	50
chr9	23665669			G	AA	GG	AA					0	57	62	0	0	47
chr9	23665682			G	CC	GG	CC					0	65	65	0	0	54
chr9	23665715			C	TT	CC	TT					0	59	58	0	0	36
chr9	23665717			G	AA	GG	AA					0	62	61	0	0	34
chr9	23665774			C	TT	CC	TT					0	54	78	0	0	34
chr9	23665793			C	GG	CC	GG					0	41	74	0	0	35
chr9	23665800			G	AA	GG	AA					0	41	74	0	0	40
chr9	23665825			A	CC	AA	CC					0	55	78	0	0	58
chr9	23665852			A	GG	AA	GG					0	65	85	0	0	61
chr9	23665855			G	AA	GG	AA					1	65	83	0	0	62
chr9	23665935			G	AA	GG	AA					0	89	67	1	0	57
chr9	23665952			T	CC	TT	CC					0	80	68	0	0	61
chr9	23665976			G	AA	GG	AA					0	81	75	0	0	73
chr9	23666035			G	TT	GG	TT					0	73	83	0	0	85
chr9	23666060			A	GG	AA	GG					0	75	74	0	0	83
chr9	23666124			G	AA	GG	AA					0	76	61	0	0	72
chr9	23666159			G	CC	GG	CC					0	81	50	0	0	74
chr9	23666190			A	CC	AA	CC					0	76	68	0	0	71
chr9	23666448			C	AA	CC	AA					0	81	70	0	0	69
chr9	23666487			G	TT	GG	TT					0	67	65	0	0	62
chr9	23666530			A	GG	AA	GG					0	72	59	0	0	61
chr9	23666643			A	CC	AA	CC					0	61	60	0	0	66
chr9	23677039			T	CC	TT	CC					0	75	59	0	0	68
chr9	23677343			A	GG	AA	GG					0	65	52	0	0	61
chr9	23677403			T	CC	TT	CC					0	72	61	0	0	71
chr9	23677409			C	TT	CC	TT					0	74	62	0	0	76
chr9	23677789			G	AA	GG	AA					0	79	74	0	0	63
chr9	23677802			T	AA	TT	AA					0	84	75	0	0	70
chr9	23678066			A	TT	AA	TT					0	57	63	0	0	63
chr9	23679038			A	GG	AA	GG					0	76	79	0	0	65
chr9	23679091			C	AA	CC	AA					0	68	70	0	0	67
chr9	23679140			C	TT	CC	TT					0	64	66	0	0	62
chr9	23679155			T	GG	TT	GG					0	70	65	0	0	66
chr9	23679174			T	CC	TT	CC					0	69	73	0	0	62
chr9	24236492	HDIVs	23763	C	TT	C	T			Yes	Yes	0	138	74	0	0	61
chr9	24237293			C	CC	T	C			Yes	Yes	195	0	0	69	78	0
chr9	24243216			A	GG	A	G			Yes	Yes	0	194	76	0	0	90
chr9	24243247			A	GG	A	G			Yes	Yes	0	188	79	0	0	89
chr9	24243335			T	TT	C	T			Yes	Yes	166	0	0	75	70	0
chr9	26004438	CNIVs		A	TT	AA	TT					0	120	48	0	0	49
chr9	31705923	CNIVs		C	GG	CG	CC					0	56	80	47	60	0
chr9	31714647			C	TT	CT	CC					0	56	87	43	71	0
chr9	65803269	CNIVs		T	TA	TT	AA	Yes				50	57	45	0	0	45
chr9	85587227	HDIVs	7932	A	G	AG	A		Yes		Yes	0	61	74	64	43	0
chr9	85588637			G	A	GA	G		Yes		Yes	0	36	46	49	46	0
chr9	85590709			G	C	GC	G		Yes		Yes	0	84	49	62	79	1
chr9	86445487	CNIVs		G	AA	GG	GG					0	38	36	0	39	0
chr9	86559771	HDIVs	20133	A	AG	A	G			Yes	Yes	68	89	77	0	0	67
chr9	86560726			T	TG	T	G			Yes	Yes	67	64	43	0	0	53
chr9	135538916	HDIVs	4278	G	GA	A	G			Yes	Yes	73	70	0	47	57	0
chr10	712587	CNIVs		G	GG	AA	GG					101	0	0	35	36	0
chr10	3817069	HDIVs	1417	G	GA	A	G			Yes	Yes	76	84	0	59	66	0
chr10	3817222			C	CC	T	C			Yes	Yes	167	0	0	77	59	0
chr10	3817286			G	GT	T	G			Yes	Yes	79	105	3	73	71	1
chr10	6997507	CNIVs		T	TA	TT	AA					48	49	43	0	2	45
chr10	9190058	CNIVs		C	GG	CG	CC					11	88	53	54	51	0
chr10	15164973	CNIVs		T	CC	TT	TT					0	38	52	0	36	0
chr10	19000982	HDIVs	1916	T	G	TG	T		Yes		Yes	0	84	71	58	65	0
chr10	19001231			T	A	TA	T		Yes		Yes	0	94	61	59	73	0
chr10	25469655	CNIVs		T	TT	AA	AA					54	0	0	101	0	52
chr10	27668494	CNIVs		T	AA	TT	TT					0	88	136	0	57	0
chr10	27669109			C	AA	CC	CC					0	80	106	0	63	0
chr10	27669258			A	TT	AA	AA					0	55	93	0	46	0
chr10	37462500	CNIVs		T	CC	TT	CC					0	48	40	0	0	35
chr10	44075655	CNIVs		C	GG	CG	CC					0	38	29	31	45	0
chr10	50350985	CNIVs		T	GG	TG	TT					0	79	63	66	68	0
chr10	53285299	CNIVs		C	AA	CC	AA					0	67	36	0	0	47
chr10	63110816	CNIVs		T	GG	TT	TT					0	69	52	0	62	0
chr10	64844481	HDIVs	3581	C	CC	A	C			Yes	Yes	164	0	0	55	51	0
chr10	64844936			A	AA	G	A			Yes	Yes	162	0	0	56	55	0
chr10	64845184			G	GG	A	G			Yes	Yes	160	0	0	68	64	0
chr10	89610068	CNIVs		G	GG	GA	AA					79	0	62	68	1	59
chr10	89678861	CNIVs		T	TT	CC	TT					75	0	0	60	76	0
chr10	120522988	CNIVs		C	TT	CC	TT					0	112	47	0	0	43
chr10	131319279	CNIVs		C	AA	CC	CC					0	61	70	0	42	0
chr10	131319297			A	CC	AA	AA					0	47	57	0	55	0
chr10	131322175			T	TT	CC	TT					98	0	0	65	56	0
chr11	7239590	*de novo* SNVs		A	AA	AA	AG					136	0	99	0	58	59
chr11	25116686	HDIVs	19316	T	T	TA	A		Yes		Yes	68	0	68	58	0	50
chr11	25118375			T	T	TG	G		Yes		Yes	74	1	72	76	0	77
chr11	25118829			T	T	TC	C		Yes		Yes	67	0	70	72	0	72
chr11	25120141			T	C	TT	T		Yes		Yes	0	88	141	0	58	0
chr11	37283989	CNIVs		T	TT	GG	GG					74	0	0	126	0	60
chr11	40081576	HDIVs	29825	G	G	GA	A		Yes		Yes	84	0	71	70	0	78
chr11	40095443			A	A	AG	G		Yes		Yes	87	0	53	68	0	68
chr11	40095656			A	A	GG	G		Yes		Yes	79	0	0	140	1	83
chr11	40096398			T	T	TC	C		Yes		Yes	78	0	54	44	0	45

chr11	40100618			A	A	AC	C		Yes		Yes	80	0	69	45	0	51
chr11	40103557			A	A	TT	T		Yes		Yes	93	0	0	142	0	58
chr11	40107391			C	C	GG	G		Yes		Yes	110	0	0	143	0	75
chr11	71754590	CNIVs		A	AG	GG	AA					63	55	0	52	58	0
chr11	71755040			C	CT	CC	TT					57	74	47	0	0	64
chr11	78130728	CNIVs		T	TT	CC	CC					30	0	0	53	0	34
chr11	88682917	CNIVs		A	AA	GG	GG					70	0	0	136	0	65
chr11	89982249	CNIVs		T	CC	TT	TT					0	42	64	0	37	0
chr11	114577466	HDIVs	1265	G	GG	A	G			Yes	Yes	119	0	5	72	71	1
chr11	114577467			A	AA	T	A			Yes	Yes	120	1	6	71	73	0
chr11	125599990	CNIVs		C	CT	CC	TT					48	34	45	0	0	36
chr11	125899273	CNIVs		G	AA	GG	AA					0	42	37	0	0	34
chr11	125992260	CNIVs		C	TT	CC	CC					6	71	156	0	75	0
chr12	1449089	CNIVs		G	GG	AA	AA					0	0	0	41	0	49
chr12	1449090			A	AA	TT	TT					0	0	0	41	0	52
chr12	1786249	*de novo* SNVs		G	GG	GG	GT			Yes	Yes	0	0	53	5	38	33
chr12	5084877	CNIVs		C	CC	TT	TT					37	0	0	44	0	44
chr12	5084878			A	AA	GG	GG					39	0	0	44	0	44
chr12	5759775	HDIVs	1932	G	GT	T	G			Yes	Yes	36	43	0	46	53	0
chr12	6637380	HDIVs	10584	T	TT	A	T			Yes	Yes	105	1	1	45	54	0
chr12	10605427	CNIVs		A	AC	CC	AA					82	70	0	66	68	0
chr12	12525997	CNIVs		G	GG	CC	CC					84	0	0	51	0	64
chr12	14055837	*de novo* SNVs		T	TT	TT	TA					32	0	49	0	35	21
chr12	15586748	CNIVs		C	TT	CC	TT					0	84	57	0	0	56
chr12	15681907	CNIVs		A	GG	AG	AA					0	46	73	49	58	0
chr12	16874642	*de novo* SNVs		C	CC	CC	CA					139	0	133	0	70	68
chr12	28800658	*de novo* SNVs		C	CC	CC	CT					170	0	131	0	71	63
chr12	48999489	*de novo* SNVs		A	AA	AA	AG					55	0	67	0	24	29
chr12	74028360	*de novo* SNVs		T	TT	TT	TA					174	0	154	0	87	54
chr12	84178923	CNIVs		G	GG	AA	GG					86	0	0	67	78	0
chr12	86655694	CNIVs		A	AA	GG	GG					57	0	0	34	0	44
chr12	86691784	CNIVs		A	AA	CC	AA					62	0	0	58	37	0
chr12	86703428			A	AA	GG	GG					47	0	0	39	0	35
chr12	87522336	*de novo* SNVs		T	TT	TT	TC					161	0	144	0	84	65
chr12	100682880	*de novo* SNVs		C	CC	CC	CT					87	0	85	0	46	42
chr12	102392564	*de novo* SNVs		G	GG	GG	GA					142	0	95	0	46	48
chr12	126806821	HDIVs	6240	G	G	AA	A		Yes		Yes	68	0	0	139	0	53
chr12	126808324			A	A	GG	G		Yes		Yes	72	0	0	114	0	50
chr12	128944118	*de novo* SNVs		C	CC	CC	CT					37	0	46	1	35	14
chr12	129573166	CNIVs		C	CC	TT	TT					70	0	0	51	0	59
chr12	130443255	CNIVs		C	AA	CC	CC					0	67	44	0	54	0
chr12	131260660	CNIVs		A	AA	CC	AA					127	0	0	75	86	0
chr12	131836440	CNIVs		A	AA	CC	AA					80	0	0	56	69	0
chr12	132069100	CNIVs		G	AA	GG	GG					0	51	40	0	59	0
chr13	53854762	CNIVs		G	GG	AA	GG					134	0	0	41	54	0
chr13	63859539	HDIVs	2130	T	TC	T	C			Yes	Yes	53	58	49	0	0	57
chr13	69895015	CNIVs		A	AA	AG	GG					65	0	71	57	0	61
chr13	80366727	HDIVs	35600	C	AA	C	C			Yes	Yes	0	111	62	0	70	0
chr13	82926273	CNIVs		T	TT	TG	GG					76	1	75	80	3	65
chr13	88604641	CNIVs		C	CC	CT	TT					98	0	71	69	0	70
chr13	88604720			T	TT	GG	GG					94	0	0	130	0	75
chr13	102880530	CNIVs		G	GG	AA	GG					171	0	0	53	80	0
chr13	114091867	CNIVs		G	AA	GG	GG					0	35	70	0	39	0
chr13	114091987			G	AA	GG	GG					0	45	59	0	36	0
chr14	23843618	CNIVs		T	AA	TT	AA					0	46	53	0	0	41
chr14	32877125	CNIVs		T	AA	TA	TT					0	71	39	42	45	1
chr14	41409503	HDIVs	11467	G	GA	A	G			Yes	Yes	85	90	0	76	78	0
chr14	41410391			C	CT	T	C			Yes	Yes	108	86	0	80	89	0
chr14	41419730			A	AC	A	C			Yes	Yes	63	56	62	0	0	62
chr14	44410299	HDIVs	64410	A	G	AG	A		Yes		Yes	0	84	55	48	59	1
chr14	44465977			A	G	AG	A		Yes		Yes	0	86	45	83	76	0
chr14	45419576	CNIVs		T	TT	CC	CC					79	0	0	83	0	69
chr14	45419720			T	GG	TT	TT					0	84	80	0	73	0
chr14	45419866			C	TT	CC	CC					0	88	68	0	63	0
chr14	45419979			A	GG	AA	AA					0	87	75	0	83	0
chr14	53480761	CNIVs		G	GG	CC	CC					69	0	0	140	0	59
chr14	55251627	CNIVs		A	AA	AG	GG					40	0	33	35	0	39
chr14	55255684	HDIVs	6534	T	A	TA	T		Yes		Yes	0	82	69	65	78	0
chr14	65693652	HDIVs	2583	T	T	TC	C		Yes		Yes	44	0	58	42	1	45
chr14	65694266			G	G	GA	A		Yes		Yes	83	0	67	67	0	65
chr14	65700747	HDIVs	9634	G	G	GA	A		Yes		Yes	77	0	81	63	0	57
chr14	65700790			A	A	AT	T		Yes		Yes	86	0	66	62	0	60
chr14	65702896			A	A	AG	G		Yes		Yes	72	0	78	80	0	63
chr14	65703128			G	G	GA	A		Yes		Yes	72	0	62	71	0	70
chr14	65708315			G	G	GA	A		Yes		Yes	76	0	69	61	0	68
chr14	84774531	CNIVs		G	GG	TT	TT	Yes				78	0	0	51	0	59
chr14	89130137	CNIVs		T	TT	AA	AA					60	0	0	99	0	49
chr14	90226506	CNIVs		A	GG	AA	GG					3	43	40	0	2	52
chr14	102515070	*de novo* SNVs		G	GG	GG	GC					152	0	121	0	75	63
chr14	102541817	*de novo* SNVs		G	GG	GG	GT					129	0	118	0	58	49
chr14	106055151	HDIVs	3913	C	CG	C	G	Yes		Yes	Yes	71	59	57	0	0	61
chr14	106057223			C	CG	C	G	Yes		Yes	Yes	65	48	51	0	0	39
chr14	106057764			G	GT	G	T	Yes		Yes	Yes	55	34	39	1	0	34
chr14	106059196			C	CT	C	T	Yes		Yes	Yes	84	79	65	0	0	66
chr14	106072856	CNIVs		A	AG	AA	GG					87	54	48	0	0	44
chr15	18346594	CNIVs		A	GG	AA	AA	Yes				0	38	85	0	66	0
chr15	20875377	HDIVs	2841	G	GC	G	C			Yes	Yes	83	83	63	0	0	56
chr15	20875539			A	GG	A	G			Yes	Yes	0	118	49	0	0	41
chr15	21765369	HDIVs	2788	C	C	CT	T	Yes	Yes		Yes	100	0	54	61	0	70
chr15	22646409	HDIVs	4899	G	GA	G	A			Yes	Yes	54	50	48	0	0	47
chr15	22646651			C	CC	T	C			Yes	Yes	81	1	0	46	56	0
chr15	22649231			A	AC	A	C			Yes	Yes	48	41	42	0	0	51
chr15	22649730			A	AG	A	G			Yes	Yes	59	53	52	0	0	54
chr15	22650182			G	GA	G	A			Yes	Yes	47	49	36	0	0	58
chr15	34648861	HDIVs	7951	T	TC	C	T			Yes	Yes	58	73	0	48	66	0
chr15	34649954			A	AG	A	G			Yes	Yes	86	64	75	0	0	67
chr15	34650735			C	CT	T	C			Yes	Yes	86	93	0	70	68	0
chr15	34651947			T	TC	T	C			Yes	Yes	82	71	100	0	0	70
chr15	34652326			G	GA	A	G			Yes	Yes	84	89	0	71	53	0
chr15	34652452			T	TC	T	C			Yes	Yes	91	66	67	0	0	43
chr15	34652612			C	CT	C	T			Yes	Yes	109	83	83	0	0	73
chr15	34652763			T	TC	T	C			Yes	Yes	77	98	57	0	0	72
chr15	34653829			G	GA	G	A			Yes	Yes	65	87	66	0	0	77
chr15	34654330			C	CT	C	T			Yes	Yes	66	79	58	0	0	56
chr15	34654333			A	AC	A	C			Yes	Yes	68	74	63	0	0	57
chr15	34654481			C	CT	C	T			Yes	Yes	56	77	43	0	0	41
chr15	34655100			A	AT	T	A			Yes	Yes	86	96	0	66	69	0
chr15	34655591			T	TC	T	C			Yes	Yes	70	76	59	0	0	56
chr15	34818070	*de novo* SNVs		C	CC	CC	CT					110	0	113	0	60	59
chr15	62786977	*de novo* SNVs		G	GG	GG	GA					124	0	111	0	59	46
chr15	83078379	*de novo* SNVs		C	CC	CC	CT					111	0	83	0	61	53
chr15	84223966	HDIVs	2094	C	T	CT	C		Yes		Yes	0	68	58	58	60	0
chr15	92432698	CNIVs		C	CT	CC	TT					51	40	42	0	0	42

chr15	92433934	CNIVs		T	TC	CC	TT					70	45	0	44	41	0
chr16	10762856	CNIVs		T	CC	TT	CC					0	108	61	0	0	54
chr16	13692303	CNIVs		T	GG	TT	TT					0	39	101	0	42	0
chr16	13692322			A	TT	AT	AA					0	38	50	42	49	0
chr16	17587869	CNIVs		T	TT	AA	TT					178	0	0	75	85	0
chr16	18039327	CNIVs		C	CA	CC	AA					58	39	46	1	0	50
chr16	19845277	CNIVs		G	GG	AA	GG					81	0	0	38	46	0
chr16	23122064	CNIVs		A	GG	AA	GG					0	116	59	0	0	55
chr16	23122682	HDIVs	2068	G	CC	G	C			Yes	Yes	0	125	58	0	0	73
chr16	26212118	CNIVs		G	GA	GG	AA					63	47	61	1	2	53
chr16	30413977	CNIVs		G	GG	AA	AA	Yes				60	0	0	136	0	71
chr16	30418159			T	GG	TT	TT	Yes				0	50	151	0	67	0
chr16	32029175	CNIVs		T	TA	TT	AA					59	73	54	0	0	47
chr16	32029421	HDIVs	5747	G	GC	C	G			Yes	Yes	55	71	1	37	46	0
chr16	33728069	CNIVs		A	AA	GG	GG	Yes				114	1	0	49	0	38
chr16	44045526	HDIVs	7020	C	CT	C	T			Yes	Yes	52	71	61	0	0	66
chr16	44466231	CNIVs		A	AA	GG	GG					29	0	0	75	0	44
chr16	52801650	CNIVs		C	CT	TT	CC					69	56	0	57	56	0
chr16	55712091	CNIVs		C	TT	CC	CC					0	76	94	0	51	0
chr16	59880106	CNIVs		C	CC	GG	GG					71	0	6	67	6	64
chr16	60977793	CNIVs		A	GG	AA	GG					0	89	41	0	0	39
chr16	63709397	CNIVs		T	TA	AA	TT					59	46	0	67	62	0
chr16	64140128	CNIVs		A	GG	AG	AA					0	92	72	73	65	0
chr16	69362469	CNIVs		C	CC	TT	CC	Yes				110	0	0	49	56	0
chr16	69369390			G	GG	TT	GG					102	0	0	58	49	0
chr16	69370927	HDIVs	1014	A	AA	G	A			Yes	Yes	180	0	0	61	58	0
chr16	69379600	CNIVs		G	GA	GG	AA					52	34	42	0	0	40
chr16	69384747			C	CC	AA	CC	Yes				140	0	0	66	49	0
chr16	69386527	HDIVs	1780	A	AG	A	G	Yes		Yes	Yes	55	69	59	0	0	62
chr16	69393022	CNIVs		T	TT	CC	TT	Yes				99	0	0	40	57	0
chr16	73035744	CNIVs		T	GG	TT	TT					5	85	142	0	62	0
chr16	73052572	HDIVs	3434	C	CT	C	T			Yes	Yes	52	56	69	0	0	58
chr16	73054170			C	CC	T	C			Yes	Yes	164	0	0	62	58	0
chr16	81618590	CNIVs		G	GG	AA	AA					33	0	0	39	0	43
chr16	89440226	CNIVs		C	GG	CC	GG					0	66	43	0	0	58
chr17	8321019	HDIVs	1678	T	TC	T	C			Yes	Yes	87	66	63	0	0	78
chr17	16254523	CNIVs		A	TT	AA	AA					0	144	88	0	40	0
chr17	16254742	*de novo* CNVs	1087	T	TT	CC	C				Yes	229	0	0	160	0	79
chr17	16255473			C	TT	CC	C				Yes	1	217	105	0	69	0
chr17	16256982	CNIVs		G	CC	GG	GG					0	132	88	0	41	0
chr17	16257052			C	GG	CC	CC					0	89	66	0	43	0
chr17	16258215	*de novo* CNVs	5842	T	TT	CC	C				Yes	198	0	0	110	0	61
chr17	16258434			A	AA	GG	G				Yes	192	0	1	124	0	90
chr17	16258901			T	CC	TT	T				Yes	0	223	156	0	90	0
chr17	16259017			C	AA	CC	C				Yes	0	222	148	0	62	0
chr17	16260038			A	AA	GG	G				Yes	168	0	0	119	0	78
chr17	16260699			G	AA	GG	G				Yes	0	144	104	0	59	0
chr17	16261832			T	TT	CC	C				Yes	122	1	0	114	0	47
chr17	16261837			T	CC	TT	T				Yes	0	129	113	0	45	0
chr17	16261840			A	AA	GG	G				Yes	128	0	0	116	0	43
chr17	16261987			T	CC	TT	T				Yes	0	191	109	0	53	0
chr17	16262037			T	TT	AA	A				Yes	132	0	0	95	0	45
chr17	16262129			G	GG	AA	A				Yes	142	0	0	101	0	61
chr17	16262153			C	CC	TT	T				Yes	170	0	0	122	0	68
chr17	16262402			T	AA	TT	T				Yes	0	134	95	0	49	0
chr17	16262524			A	CC	AA	A				Yes	0	196	149	0	71	1
chr17	16262548			T	TT	CC	C				Yes	186	0	0	138	0	69
chr17	16263385	HDIVs	8982	A	G	AA	A		Yes		Yes	0	70	101	0	57	0
chr17	16271928			C	G	CC	C		Yes		Yes	0	77	93	0	41	0
chr17	16273504	CNIVs		T	CC	TT	TT					0	94	145	0	84	0
chr17	16273711			C	TT	CC	CC					0	88	121	0	67	0
chr17	16293755	CNIVs		A	TT	AA	AA					1	107	144	0	55	0
chr17	52058123	CNIVs		A	AA	AC	CC					85	0	58	72	0	57
chr17	52666591	*de novo* SNVs		G	GG	GG	GT					165	0	111	0	51	49
chr17	64905713	CNIVs		A	GG	AA	AA					0	82	149	0	73	0
chr17	70607918	CNIVs		T	TT	TC	CC					59	0	54	56	0	56
chr17	72059473	*de novo* CNVs	1869	T	CC	TC	T				Yes	0	98	55	52	40	0
chr17	72059498			G	AA	GA	G				Yes	0	99	61	65	39	0
chr17	72059549			C	GG	CG	C				Yes	0	118	65	75	52	0
chr17	72059665			A	CC	AC	A				Yes	0	95	54	55	44	0
chr17	72059811			C	AA	CA	C				Yes	0	70	57	37	58	0
chr18	16467	CNIVs		C	CC	CT	TT	Yes				47	0	49	37	0	44
chr18	5689878	CNIVs		T	TT	CC	TT					120	0	0	55	52	0
chr18	7836474	*de novo* SNVs		C	CC	CC	CT					113	0	106	0	59	63
chr18	11156493	CNIVs		A	GG	AA	AA					0	52	67	1	43	0
chr18	19782731	CNIVs		A	AA	TT	TT					86	1	0	38	0	43
chr18	27325738	CNIVs		G	GC	GG	CC					71	72	54	0	0	48
chr18	38077525	CNIVs		A	CC	AA	AA					0	71	59	0	71	0
chr18	38077537			A	TT	AA	AA					0	71	56	0	74	0
chr18	41177699	CNIVs		A	GG	AA	GG					0	56	34	0	0	41
chr18	46129982	CNIVs		A	GG	AA	AA					0	36	36	0	43	0
chr18	60316217	CNIVs		G	GT	GG	TT					76	77	71	0	4	67
chr18	60669765	CNIVs		A	AA	TT	AA					175	0	0	62	59	1
chr18	60669766			A	AA	TT	AA					175	0	1	61	60	0
chr18	62012908	CNIVs		T	TT	TC	CC					96	0	62	61	5	80
chr18	68811160	*de novo* CNVs	2851	T	TT	TG	G				Yes	56	0	60	45	0	42
chr18	68811162			G	GG	GA	A				Yes	57	0	59	46	0	42
chr18	68811232			C	CC	CT	T				Yes	77	0	73	59	0	85
chr18	68811588			T	TT	TC	C				Yes	86	0	63	55	0	62
chr18	68811835			C	CC	CT	T				Yes	105	0	63	66	0	64
chr19	1242579	CNIVs		G	GG	GA	AA					78	0	30	45	1	39
chr19	1474463	*de novo* SNVs		C	CC	CC	CT					90	0	83	0	55	42
chr19	1748679	*de novo* SNVs		G	GG	GG	GA					111	0	85	0	43	64
chr19	22488011	CNIVs		A	AG	AA	GG					67	57	69	0	0	54
chr19	22488761			A	AC	AA	CC					78	67	90	0	0	50
chr19	22488766			T	TC	TT	CC					77	65	89	0	0	47
chr19	35760551	CNIVs		G	GT	GG	TT					42	55	41	0	0	37
chr19	37879905	*de novo* SNVs		A	AA	AA	AT					110	0	99	0	48	47
chr19	48250486	CNIVs		A	GG	AA	GG					0	67	31	0	0	34
chr19	53716681	HDIVs	9568	C	C	CT	T		Yes		Yes	94	0	70	65	0	47
chr19	57737681	CNIVs		A	AA	CC	AA					109	0	0	37	38	0
chr19	59659920	CNIVs		A	GG	AA	AA					0	56	91	0	50	0
chr19	59660087			T	AA	TA	TT					0	66	49	44	39	0
chr19	59660702			C	TT	CC	CC					0	68	95	0	53	0
chr19	59660723			T	CC	TT	TT					0	63	86	0	47	0
chr19	59700449	CNIVs		A	AA	GG	AA					91	0	0	40	46	0
chr19	59702746	HDIVs	2067	A	CC	A	C			Yes	Yes	0	91	47	0	0	51
chr19	59704117			T	TC	T	C			Yes	Yes	65	64	48	0	0	47
chr19	59704310			C	CA	C	A			Yes	Yes	46	50	55	0	0	50
chr19	59713538	CNIVs		C	AA	CC	AA					0	152	54	0	0	74
chr20	2013710	CNIVs		C	CC	AA	CC					69	0	0	48	58	0
chr20	4563857	*de novo* SNVs		C	CC	CC	CT					194	0	141	0	62	67
chr20	11128300	CNIVs		A	AA	AG	GG					75	0	52	70	6	75

chr20	12503091	HDIVs	8104	T	T	TC	C		Yes		Yes	64	1	52	63	0	56
chr20	12508872			T	C	TC	T		Yes		Yes	0	67	59	39	73	0
chr20	13465369	*de novo* CNVs	3218	C	CC	CT	T				Yes	120	0	61	72	0	75
chr20	13465680			T	TT	TC	C				Yes	120	0	66	59	0	61
chr20	15631596	*de novo* SNVs		C	CC	CC	CT					157	0	104	0	75	83
chr20	20375387	CNIVs		A	GG	AG	AA					0	59	50	58	61	0
chr20	20734897	CNIVs		G	AA	GA	GG					3	61	57	68	76	0
chr20	20734898			A	GG	AG	AA					0	64	57	66	71	0
chr20	21272566	CNIVs		A	AG	AA	GG					55	46	58	0	0	43
chr20	24985089	HDIVs	2430	T	TC	C	T			Yes	Yes	75	55	0	44	77	1
chr20	24985091			T	TG	G	T			Yes	Yes	75	54	0	45	79	0
chr20	24985092			C	CG	G	C			Yes	Yes	74	55	0	44	77	0
chr20	27834367	*de novo* CNVs	1522	C	CC	AA	A				Yes	37	0	0	66	0	38
chr20	52716788	CNIVs		A	CC	AA	AA					9	100	125	0	82	0
chr20	52716811			G	TT	GG	GG					10	91	134	0	78	0
chr20	55511398	CNIVs		A	GG	AG	AA					0	61	50	38	61	0
chr20	57267629	CNIVs		C	CC	CT	TT					69	0	54	49	4	72
chr20	59786779	CNIVs		C	GG	CC	CC					0	71	75	0	57	0
chr20	61591188	CNIVs		A	AG	GG	AA					55	40	2	63	56	0
chr22	14439443	HDIVs	1029	G	GG	A	G	Yes		Yes	Yes	127	0	0	40	44	0
chr22	14441553	CNIVs		G	GG	CC	GG	Yes				116	0	0	46	65	0
chr22	14441580			A	AA	GG	AA	Yes				136	0	0	58	81	0
chr22	14453010	HDIVs	5291	A	AA	C	A	Yes		Yes	Yes	82	0	0	39	49	0
chr22	14453673			T	TT	C	T	Yes		Yes	Yes	99	0	0	35	54	0
chr22	14454435			G	GG	C	G	Yes		Yes	Yes	135	0	0	52	61	0
chr22	14454438			A	AA	T	A	Yes		Yes	Yes	138	0	0	52	61	0
chr22	14454488			C	CC	T	C	Yes		Yes	Yes	150	0	0	63	68	1
chr22	14454491			C	CC	T	C	Yes		Yes	Yes	151	0	0	61	70	0
chr22	14454519			T	TT	A	T	Yes		Yes	Yes	174	0	0	43	67	0
chr22	14454652			T	TT	A	T	Yes		Yes	Yes	195	0	0	44	72	0
chr22	14454724			G	GG	A	G	Yes		Yes	Yes	121	0	0	38	50	0
chr22	14455200			G	GG	C	G	Yes		Yes	Yes	207	0	0	55	69	0
chr22	14455208			C	CC	A	C	Yes		Yes	Yes	210	0	0	57	65	0
chr22	14455249			T	TT	C	T	Yes		Yes	Yes	223	0	1	71	67	0
chr22	14455292			G	GG	C	G	Yes		Yes	Yes	200	0	0	78	68	0
chr22	14455302			G	GG	A	G	Yes		Yes	Yes	186	0	0	72	63	0
chr22	14455370			C	CC	T	C	Yes		Yes	Yes	172	0	0	41	52	0
chr22	14455422			T	TT	C	T	Yes		Yes	Yes	194	0	0	47	52	0
chr22	14458456	CNIVs		C	CC	TT	CC	Yes				133	0	0	44	60	0
chr22	18799394	CNIVs		G	AA	GG	GG	Yes				0	97	96	0	38	0
chr22	20391616	CNIVs		A	GG	AG	AA					0	112	50	89	57	0
chr22	21235687	HDIVs	39736	T	TC	C	T			Yes	Yes	49	51	0	39	58	0
chr22	21238015			T	TT	G	T			Yes	Yes	103	1	0	46	69	0
chr22	21242855			A	AG	G	A			Yes	Yes	49	53	0	53	60	0
chr22	21244567			C	CT	T	C			Yes	Yes	61	43	0	52	56	0
chr22	21246381			G	GG	A	G			Yes	Yes	105	0	0	58	74	0
chr22	21246491			T	TT	G	T			Yes	Yes	122	0	0	68	65	0
chr22	21247673			G	GG	A	G			Yes	Yes	132	0	0	61	58	0
chr22	21247865			A	AG	G	A			Yes	Yes	50	50	0	47	56	0
chr22	21249882			G	GT	T	G			Yes	Yes	71	59	0	57	61	0
chr22	21251604			G	GA	A	G			Yes	Yes	70	63	0	69	61	0
chr22	21252583			C	CC	T	C			Yes	Yes	154	0	0	67	70	0
chr22	21252819			G	GA	A	G			Yes	Yes	87	59	0	56	66	0
chr22	21260280			A	AA	T	A			Yes	Yes	175	0	0	66	69	0
chr22	21263564			C	CC	T	C			Yes	Yes	158	0	0	68	59	0
chr22	21268825			C	CC	G	C			Yes	Yes	102	0	0	40	41	0
chr22	21269780			C	CC	T	C			Yes	Yes	155	0	0	64	58	0
chr22	21269913			T	TT	G	T			Yes	Yes	122	0	0	69	54	0
chr22	21270157			A	AG	G	A			Yes	Yes	44	42	0	50	51	0
chr22	21270544			C	CC	T	C			Yes	Yes	86	0	0	44	41	0
chr22	21270767			T	TC	C	T			Yes	Yes	47	45	0	42	45	0
chr22	21272760	*de novo* CNVs	11284	T	TT	CC	T				Yes	187	0	0	127	70	1
chr22	21272798			G	GG	AA	G				Yes	168	0	0	117	68	0
chr22	21272863			A	AA	GG	A				Yes	136	0	0	119	57	0
chr22	21273117			A	AA	CC	A				Yes	183	0	0	155	63	1
chr22	21273625			T	TT	CC	T				Yes	121	0	0	99	64	0
chr22	21274386			C	CC	TT	C				Yes	108	0	0	96	47	0
chr22	21275003			C	CC	AA	C				Yes	103	0	0	92	48	0
chr22	21275390			C	CC	TT	C				Yes	115	0	0	120	49	0
chr22	21276006			C	CC	GG	C				Yes	131	0	0	47	75	0
chr22	21280694			G	GA	AA	G				Yes	67	57	0	117	56	0
chr22	21280695			A	AA	GG	A				Yes	125	0	0	118	56	0
chr22	21280916			A	AA	GG	A				Yes	104	0	0	79	53	0
chr22	21281821			C	CC	AA	C				Yes	112	0	0	122	64	0
chr22	21282113			A	AA	GG	A				Yes	108	0	0	106	59	0
chr22	21282310			A	AA	GG	A				Yes	124	0	0	84	51	0
chr22	21282776			G	GG	AA	G				Yes	169	0	1	111	62	0
chr22	21283125	HDIVs	3478	G	GG	A	G			Yes	Yes	110	0	0	66	36	0
chr22	21283187			G	GG	A	G			Yes	Yes	114	0	0	86	42	0
chr22	21283276			T	TT	C	T			Yes	Yes	122	0	0	60	62	0
chr22	21283367			G	GG	A	G			Yes	Yes	100	0	0	39	73	0
chr22	21283417			T	TT	C	T			Yes	Yes	119	0	0	77	68	0
chr22	21283489			C	CC	T	C			Yes	Yes	91	0	0	46	40	0
chr22	21283491			C	CC	A	C			Yes	Yes	91	0	0	49	39	0
chr22	21283508			T	TT	G	T			Yes	Yes	72	0	0	57	42	0
chr22	21283699			A	AA	G	A			Yes	Yes	96	0	0	37	58	0
chr22	21283713			T	TT	C	T			Yes	Yes	97	0	0	40	54	0
chr22	21283749			C	CC	A	C			Yes	Yes	91	0	0	68	54	0
chr22	21283762			A	AA	T	A			Yes	Yes	91	0	0	73	51	0
chr22	21284175			G	GG	A	G			Yes	Yes	105	1	1	47	36	0
chr22	21284244			T	TT	G	T			Yes	Yes	119	0	0	41	52	1
chr22	21932435	HDIVs	2439	C	CC	T	C	Yes		Yes	Yes	99	0	2	50	34	0
chr22	22079865	*de novo* SNVs		T	TT	TT	TC					159	0	127	0	70	90
chr22	22163245	*de novo* SNVs		G	GC	GG	GA	Yes				153	76	128	0	145	50
chr22	39911445	CNIVs		C	TT	CC	CC					0	64	108	0	48	0
chr22	41592669	*de novo* SNVs		G	GG	GG	GA					95	0	86	1	43	55
chr22	45780810	*de novo* CNVs	2545	G	GG	AA	G				Yes	107	0	0	61	43	0
chr22	45781377			A	AT	AA	T				Yes	58	73	43	0	0	45
chr22	45781705	CNIVs		C	CT	CC	TT					76	90	64	0	0	45
chr22	45782916	HDIVs	1738	C	CG	C	G			Yes	Yes	67	82	57	0	0	59
chr22	48160862	CNIVs		C	CC	CT	TT					60	0	53	36	0	51
chr22	48373922	CNIVs		G	AA	GG	GG					0	66	123	0	59	0

[a] The size of deletion in the hemizygouse deletion inherited variant sites (HDIVs) and *de novo* CNVs is estimated by the program for detecting copy number changes (VarScan ver2.3.5).

[b] Dataset of segmental duplication in chimmpanzees are taken after reference 32 and http://humanparalogy.gs.washington.edu/pantro2wgac/panTro2-chimpWSSD_2007Oct23.tab

CNIVs; copy number neutral inherited variants, HDIVs; hemizygous deletion inherited variants

Supplementary Table S5. Blood and hair follicle derived DNA genotypes determined by NGS and Sanger sequencing in a chimpanzee trio.

chromosome	position	reference	Father Blood NGS	Mother Blood NGS	Offspring Blood NGS	Father Blood Sanger	Mother Blood Sanger	Offspring Blood Sanger	Offspring Hair Sanger	*de novo* SNVs	germline or somatic SNVs
chr1	13552700	C	CC	CC	**CT**	CC	CC	**CC**	**CC**	**False Positive**	-
chr2A	102577476[#]	C	**CT**	CC	**CG**	**CGT**	CC	**CGT**	**CGT**	**False Positive**	-
chr2B	140770230	G	GG	GG	GA	GG	GG	GA	GA	True Positive	Germline
chr2B	201447131	G	GG	GG	GC	GG	GG	GC	GC	True Positive	Germline
chr3	89547303	G	GG	GG	GA	GG	GG	GA	**GG**	True Positive	**Somatic**
chr4	128850953	C	CC	CC	CT	CC	CC	CT	CT	True Positive	Germline
chr5	93480078	A	AA	AA	AG	AA	AA	AG	AG	True Positive	Germline
chr5	144330507	G	GG	GG	GT	GG	GG	GT	GT	True Positive	Germline
chr6	7711997	A	AG	AA	**AT**	AG	AA	**AG**	**AG**	**False Positive**	-
chr6	12022852	G	GG	GG	**GT**	GG	GG	**GG**	**GG**	**False Positive**	-
chr6	33261071	T	**TT**	**TT**	TA	**TA**	**TA**	TA	TA	**False Positive**	-
chr6	57788180	C	CC	CC	CT	CC	CC	CT	CT	True Positive	Germline
chr6	68971246	G	GG	GG	GT	GG	GG	GT	GT	True Positive	Germline
chr6	130931815	C	CC	CC	CG	CC	CC	CG	CG	True Positive	Germline
chr8	16568611	G	GG	GG	GA	GG	GG	GA	GA	True Positive	Germline
chr8	37041261	G	GG	GG	GA	GG	GG	GA	GA	True Positive	Germline
chr8	65944369	C	CC	CC	CT	CC	CC	CT	CT	True Positive	Germline
chr8	142414336	G	GG	GG	GA	GG	GG	GA	GA	True Positive	Germline
chr11	7239590	A	AA	AA	AG	AA	AA	AG	AG	True Positive	Germline
chr12	14055837	T	**TT**	TT	TA	**TA**	TT	TA	TA	**False Positive**	-
chr12	16874642	C	CC	CC	CA	CC	CC	CA	CA	True Positive	Germline
chr12	28800658	C	CC	CC	**CT**	CC	CC	**CC**	**CC**	**False Positive**	-
chr12	74028360	T	TT	TT	TA	TT	TT	TA	TA	True Positive	Germline
chr12	87522336	T	TT	TT	TC	TT	TT	TC	TC	True Positive	Germline
chr12	100682880	C	CC	CC	CT	CC	CC	CT	CT	True Positive	Germline
chr12	102392564	G	GG	GG	GA	GG	GG	GA	GA	True Positive	Germline
chr14	102515070	G	GG	GG	GC	GG	GG	GC	GC	True Positive	Germline
chr14	102541817	G	GG	GG	GT	GG	GG	GT	GT	True Positive	Germline
chr15	34818070	C	CC	CC	CT	CC	CC	CT	CT	True Positive	Germline
chr15	83078379	C	CC	CC	CT	CC	CC	CT	CT	True Positive	Germline
chr17	52666591	G	GG	GG	GT	GG	GG	GT	GT	True Positive	Germline
chr18	7836474	C	CC	CC	CT	CC	CC	CT	CT	True Positive	Germline
chr19	1474463	C	CC	CC	CT	GG	CC	CT	CT	True Positive	Germline
chr19	1748679	G	GG	GG	GA	GG	GG	GA	GA	True Positive	Germline
chr19	37879905	A	AA	AA	AT	AA	AA	AT	AT	True Positive	Germline
chr20	4563857	C	CC	CC	CT	CC	CC	CT	CT	True Positive	Germline
chr20	15631596	C	CC	CC	CT	CC	CC	CT	CT	True Positive	Germline
chr22	22079865	T	TT	TT	TC	TT	TT	TC	TC	True Positive	Germline
chr22	22163245[#]	G	**GC**	GG	GA	**GA**	GG	GA	GA	**False Positive**	-
chr22	41592669	G	GG	GG	GA	GG	GG	GA	GA	True Positive	Germline

[#]Know segmental duplication regions in chimpanzees[32].

Supplementary Table S6. Sensitivity and specificity of two *de novo* mutation detection programs

Chr	Position	Our Methods (45)	DeNovoGear (61)	Sanger Sequencing	TP or FP or TN
chr1	2694332	×	○	YES	True Negative
chr1	13552700	○	×	YES	False Positive
chr1	98183137	×	○	NO	
chr2A	837910	×	○	NO	
chr2A	102577476	○	×	YES	False Positive
chr2B	121088150	×	○	NO	
chr2B	131984509	×	○	NO	
chr2B	140770230	○	○	YES	True Positive
chr2B	201447131	○	○	YES	True Positive
chr2B	241607083	○	×	NO	
chr3	89547303	○	○	YES	True Positive (Somatic)
chr3	105499624	×	○	NO	
chr3	106736425	×	○	NO	
chr3	201706151	×	○	YES	True Negative
chr4	128850953	○	○	YES	True Positive
chr5	16265033	×	○	NO	
chr5	93480078	○	○	YES	True Positive
chr5	144330507	○	○	YES	True Positive
chr6	7711997	○	×	YES	False Positive
chr6	12022852	○	×	YES	False Positive
chr6	33261071	○	×	YES	False Positive
chr6	57788180	○	○	YES	True Positive
chr6	64983276	×	○	NO	
chr6	64983278	×	○	NO	
chr6	68971246	○	○	YES	True Positive
chr6	73652409	×	○	YES	True Negative
chr6	130931815	○	○	YES	True Positive
chr8	8419961	×	○	NO	
chr8	16568611	○	○	YES	True Positive
chr8	29532927	×	○	YES	True Negative
chr8	37041261	○	○	YES	True Positive
chr8	65944369	○	○	YES	True Positive
chr8	142414336	○	○	YES	True Positive
chr9	72515860	×	○	NO	
chr10	126788001	×	○	NO	
chr11	7239590	○	○	YES	True Positive
chr12	1786249	○	×	NO	
chr12	14055837	○	×	YES	False Positive
chr12	16874642	○	○	YES	True Positive
chr12	28800658	○	○	YES	False Positive
chr12	48999489	○	×	NO	
chr12	74028360	○	○	YES	True Positive
chr12	87522336	○	○	YES	True Positive
chr12	100682880	○	○	YES	True Positive
chr12	102392564	○	○	YES	True Positive
chr12	128944118	○	○	NO	
chr13	98883396	×	○	NO	
chr14	102515070	○	○	YES	True Positive
chr14	102541817	○	○	YES	True Positive
chr15	21679458	×	○	YES	True Negative
chr15	34818070	○	○	YES	True Positive
chr15	62786977	○	○	NO	
chr15	83078379	○	○	YES	True Positive
chr15	98854686	×	○	NO	
chr15	98854693	×	○	NO	
chr16	55714938	×	○	NO	
chr16	55714952	×	○	NO	
chr17	34345520	×	○	YES	True Negative
chr17	52666591	○	○	YES	True Positive
chr17	79441611	×	○	NO	
chr18	18055	×	○	NO	
chr18	7836474	○	○	YES	True Positive
chr19	1474463	○	○	YES	True Positive
chr19	1748679	○	○	YES	True Positive
chr19	37879905	○	○	YES	True Positive
chr19	56060294	×	○	YES	True Negative
chr20	4563857	○	○	YES	True Positive
chr20	15631596	○	○	YES	True Positive
chr22	22079865	○	○	YES	True Positive
chr22	22163245	○	×	YES	False Positive
chr22	41592669	○	○	YES	True Positive

Supplementary Table S7. Candidate *de novo* SNV sites using four different sequence coverage data (30x, 60x, 90x, 120x).

chromosome	position	Father 30x	Mother 30x	Offspring 30x	Father 60x	Mother 60x	Offspring 60x	Father 90x	Mother 90x	Offspring 90x	Father 120x	Mother 120x	Offspring 120x	Sanger Validation	Segmental Duplication
chr1	2844695	TT	TT	TC	TT	TC	TC	TT	TC	TC	TT	TC	TC		
chr1	3261227	TC	TT	TC	TT	TT	TC	TC	TT	TC	TC	TT	TC		
chr1	3261228	GA	GG	GA	GG	GG	GA	GA	GG	GA	GA	GG	GA		
chr1	3671082	TT	TT	TC	TT	TC	TC	TT	TC	TC	TT	TC	TC		
chr1	6877649	AG	AA	AG	AA	AA	AG	AG	AA	AG	AG	AA	AG		
chr1	8547647	TT	TT	TG	TT	TG	TG	TT	TG	TG	TT	TG	TG		
chr1	13552700	CC	CC	CC	CC	CC	CT	CC	CC	CT	CC	CC	CT	False Positive	
chr1	17907110	TT	TT	TC	TC	TT	TC	TC	TT	TC	TC	TT	TC		
chr1	20151276	CC	CC	CT	CC	CC	CT	CT	CC	CT	TT	CC	CT		
chr1	21499008	GG	GG	GA	GG	GA	GA	GG	GA	GA	GG	GA	GA		
chr1	30344618	GG	GG	GA	GG	GA	GA	GG	GA	GA	GG	GA	GA		
chr1	54299355	TT	TT	TA	TA	TT	TA	TA	TT	TA	TA	TT	TA		
chr1	102866784	TT	TT	TA	TT	TA	TA	TT	TA	TA	TT	TA	TA		
chr1	117136508	TT	TT	TC	TT	TC	TC	TT	TC	TC	TT	TC	TC		
chr1	124917294	CT	CC	CT	CC	CC	CT	CT	CC	CT	CT	CC	CT		
chr1	140000750	GG	GG	GA	GG	GA	GA	GG	GA	GA	GG	GA	GA		
chr1	141734455	AA	AA	AT	AT	AA	AT	AT	AA	AT	AT	AA	AT		
chr1	148082648	TT	TT	TC	TC	TT	TC	TC	TT	TC	TC	TT	TC		
chr1	153445275	GG	GG	GA	GA	GG	GA	GA	GG	GA	GA	GG	GA		
chr1	167810831	AA	AA	AT	AA	AT	AT	AA	AT	AT	AA	AT	AT		
chr1	183478824	GG	GG	GA	GG	GA	GA	GG	GA	GA	GG	GA	GA		
chr1	185145308	CC	CC	CA	CA	CA	CA	CA	CA	CA	CA	CA	CA		
chr1	185600930	GG	GG	GA	GG	GA	GA	GG	GA	GA	GG	GA	GA		
chr1	185932229	CC	CC	CT	CT	CC	CT	CT	CC	CT	CT	CC	CT		
chr1	188929595	TT	TT	TC	TT	TC	TC	TT	TC	TC	TT	TC	TC		
chr1	217014639	AA	AA	AG	AA	AG	AG	AA	AG	AG	AA	AG	AG		
chr1	221065231	TC	TT	TC	TT	TT	TC	TC	TT	TC	TC	TT	TC		
chr1	224889949	GG	GC	GG	GT	GC	GT	GT	GT	GC	GT	GT	GT	ND	
chr1	225118808	TC	TT	TC	TT	TT	TC	TC	TT	TC	TC	TT	TC		
chr1	225126354	CC	CC	CT	CT	CC	CT	CT	CC	CT	CT	CC	CT		
chr1	226989183	AA	AA	AG	AG	AA	AG	AG	AA	AG	AG	AA	AG		
chr1	227935882	GG	GG	GA	GG	GA	GA	GG	GA	GA	GG	GA	GA		
chr2A	826550	TT	TC	TC	TT	TT	TC	TT	TC	TC	TT	TC	TC		
chr2A	2850670	GG	GG	GA	GG	GA	GA	GG	GA	GA	GG	GA	GA		
chr2A	2850671	CC	CC	CT	CC	CT	CT	CC	CT	CT	CC	CT	CT		
chr2A	5977951	CC	CC	CT	CT	CC	CT	CT	CC	CT	TT	CC	CT		
chr2A	12239108	CC	CC	CT	CC	CT	CT	CC	CT	CT	CC	CT	CT		
chr2A	16626629	GG	GG	GA	GG	GA	GA	GG	GA	GA	GG	GA	GA		
chr2A	23991151	CC	CC	CT	CT	CC	CT	CT	CC	CT	CT	CC	CT		
chr2A	25222112	GG	GG	GA	GA	GG	GA	GA	GG	GA	GA	GG	GA		
chr2A	30380099	CC	CC	CA	CC	CA	CA	CC	CA	CA	CC	CA	CA		
chr2A	52563649	CC	CC	CT	CT	CC	CT	CT	CC	CT	CT	CC	CT		
chr2A	52796386	AA	AA	AC	AC	AA	AC	AC	AA	AC	AC	AA	AC		
chr2A	72983184	AA	AA	AG	AA	AG	AG	AA	AG	AG	AA	AG	AG		
chr2A	77507092	GG	GG	GA	GA	GG	GA	GA	GG	GA	GA	GG	GA		
chr2A	86513584	CC	CC	CA	CC	CA	CA	CC	CA	CA	CC	CA	CA		
chr2A	87155241	GG	GG	GA	GG	GA	GA	GG	GA	GA	GG	GA	GA		
chr2A	89116561	TT	TT	TC	TC	TT	TC	TC	TT	TC	TC	TT	TC		Seg Dup
chr2A	95784594	TT	TT	TG	TT	TG	TG	TT	TG	TG	TT	TG	TG		Seg Dup
chr2A	95970035	CA	CA	CA	CA	CA	CA	CA	CA	CG	CA	CG	CG	False Positive	Seg Dup
chr2A	96291465	GG	GG	GA	GA	GG	GA	GA	GG	GA	GA	GG	GA		Seg Dup
chr2A	102577476	CG	CC	CG	CT	CC	CG	CT	CC	CG	CT	CC	CG	False Positive	Seg Dup
chr2B	127450787	GG	GG	GC	GG	GC	GC	GG	GC	GC	GG	GC	GC		
chr2B	127456344	AA	AA	AG	AA	AG	AG	AA	AG	AG	AA	AG	AG		
chr2B	131226654	CC	CC	CT	CC	CT	CT	CC	CT	CT	CC	CT	CT		Seg Dup
chr2B	131959313	GG	GG	GA	GG	GA	GA	GG	GA	GA	GG	GA	GA		Seg Dup
chr2B	132093093	TT	TT	TC	TT	TC	TC	TT	TC	TC	TT	TC	TC		Seg Dup
chr2B	136881020	AA	AA	AC	AC	AA	AC	AC	AA	AC	AC	AA	AC		
chr2B	140770230	GG	GG	GA	GG	GG	GA	GG	GG	GA	GG	GG	GA	True Positive	
chr2B	156783998	CC	CC	CT	CT	CC	CT	CT	CC	CT	CT	CC	CT		
chr2B	173126062	AA	AA	AG	AA	AG	AG	AA	AG	AG	AA	AG	AG		
chr2B	195446022	AA	AA	AG	AA	AA	AG	AA	AG	AG	AA	AG	AG		
chr2B	201447131	GG	GG	GC	GG	GG	GC	GG	GG	GC	GG	GG	GC	True Positive	
chr2B	213086230	GG	GG	GA	GG	GA	GA	GG	GA	GA	GG	GA	GA		
chr2B	223911225	CC	CC	CG	CC	CG	CG	CC	CG	CG	CC	CG	CG		
chr2B	230414045	GG	GG	GA	GA	GG	GA	GA	GG	GA	GA	GG	GA		
chr2B	237182977	TT	TT	TC	TT	TC	TC	TT	TC	TC	TT	TC	TC		
chr2B	239553644	AA	AA	AT	AT	AA	AT	AT	AA	AT	TT	AA	AT		
chr2B	241607083	TC	TC	TG	TG	TC	TC	TG	TG	TC	TG	TG	TC	ND	
chr2B	244385541	TT	TT	TC	TT	TC	TC	TT	TC	TC	TT	TC	TC		
chr2B	244407415	CC	CC	CA	CC	CA	CA	CC	CA	CA	CC	CA	CA		
chr2B	245039845	AA	AA	AG	AA	AG	AG	AA	AG	AG	AA	AG	AG		
chr2B	246872778	TT	TT	TG	TT	TG	TG	TT	TG	TG	TT	TG	TG		
chr3	371731	AA	AA	AG	AG	AA	AG	AG	AA	AG	AG	AA	AG		
chr3	5653078	GG	GG	GA	GA	GG	GA	GA	GG	GA	GA	GG	GA		
chr3	5878713	AA	AA	AG	AA	AG	AG	AA	AG	AG	AA	AG	AG		
chr3	9219482	GG	GG	GC	GC	GG	GC	GC	GG	GC	GC	GG	GC		
chr3	9275428	GG	GG	GA	GG	GA	GA	GG	GA	GA	GG	GA	GA		
chr3	11666133	CC	CC	CA	CC	CC	CA	CC	CA	CA	CC	CA	CA		
chr3	12747178	AA	AA	AT	AA	AT	AT	AA	AT	AT	AA	AT	AT		
chr3	18868070	GG	GG	GA	GA	GG	GA	GA	GG	GA	GA	GG	GA		
chr3	25730294	CC	CC	CA	CA	CC	CA	CA	CC	CA	CA	CC	CA		
chr3	25730314	GG	GG	GA	GA	GG	GA	GA	GG	GA	GA	GG	GA		
chr3	34405021	GG	GG	GA	GA	GG	GA	GA	GG	GA	GA	GG	GA		Seg Dup
chr3	39881027	AA	AA	AG	AA	AG	AG	AA	AG	AG	AA	AG	AG		
chr3	41517459	CC	CC	CA	CC	CA	CA	CC	CA	CA	CC	CA	CA		
chr3	62612231	AA	AA	AG	AA	AG	AG	AA	AG	AG	AA	AG	AG		
chr3	63058656	AA	AA	AG	AG	AA	AG	AG	AA	AG	AG	AA	AG		
chr3	89547303	GG	GG	GA	GG	GG	GA	GG	GG	GA	GG	GG	GA	True Positive	
chr3	98397862	CC	CT	CC	CC	CC	CT	CC	CC	CT	CT	TT	CT	False Positive	
chr3	114884727	TT	TT	TC	TT	TC	TC	TT	TC	TC	TT	TC	TC		
chr3	114884733	GG	GG	GA	GG	GA	GA	GG	GA	GA	GG	GA	GA		
chr3	126259395	TT	TT	TC	TT	TC	TC	TT	TC	TC	TT	TC	TC		
chr3	132720780	GG	GG	GA	GG	GA	GA	GG	GA	GA	GG	GA	GA		
chr3	133550932	TT	TT	TA	TT	TA	TA	TT	TA	TA	TT	TA	TA		Seg Dup
chr3	138066271	AA	AA	AT	AA	AT	AT	AA	AT	AT	AA	TT	AT		

chr3	144280237	CC	CC	CT	CC	CT	CT	CC	CT	CT	CC	CT	CT		
chr3	144426915	AA	AA	AG	AA	AG	AG	AA	AG	AG	AA	AG	AG		
chr3	146534603	TT	TT	TC	TT	TC	TC	TT	TC	TC	TT	TC	TC		
chr3	153957262	TT	TT	TC	TC	TT	TC	TC	TT	TC	TC	TT	TC		
chr3	158378574	CC	CC	CT	CT	CC	CT	CT	CC	CT	CT	CC	CT		
chr3	164545327	AA	AA	AG	AA	AG	AG	AA	AG	AG	AA	AG	AG		
chr3	178439096	CC	CC	CA	CC	CA	CA	CC	CA	CA	CC	CA	CA		
chr3	180636675	GG	GG	GT	GT	GG	GT	GT	GG	GT	GT	GG	GT		
chr3	190889794	CC	CC	CT	CT	CC	CT	CT	CC	CT	TT	CC	CT		
chr3	198421719	GG	GG	GT	GG	GT	GT	GG	GT	GT	GG	GT	GT		
chr3	199323981	TT	TT	TG	TT	TG	TG	TT	TG	TG	TT	TG	TG		
chr4	1093943	AA	AA	AG	AG	AA	AG	AG	AA	AG	AG	AA	AG		
chr4	3569557	CC	CC	CG	CC	CG	CG	CC	CG	CG	CC	CG	CG		
chr4	4183423	TT	TT	TC	TT	TC	TC	TT	TC	TC	TT	TC	TC		
chr4	4183436	GG	GG	GC	GG	GC	GC	GG	GC	GC	GG	GC	GC		
chr4	6188930	TT	TT	TC	TT	TC	TC	TT	TC	TC	TT	TC	TC		
chr4	13636044	AA	AA	AG	AG	AG	AG	AG	AG	AG	AG	AG	AG		
chr4	22833173	GG	GG	GT	GT	GG	GT	GT	GG	GT	GT	GG	GT		
chr4	39334116	TT	TT	TA	TT	TA	TA	TT	TA	TA	TT	TA	TA		
chr4	40047430	CC	CC	CG	CG	CC	CG	CG	CC	CG	GG	CC	CG		
chr4	49018319	GG	GG	GA	GG	GA	GA	GG	GA	GA	GG	GA	GA		
chr4	52940964	AA	AA	AT	AA	AT	AT	AA	AT	AT	AA	AT	AT		
chr4	76222977	CC	CC	CT	CC	CT	CT	CC	CT	CT	CC	CT	CT		
chr4	118258971	AA	AA	AT	AA	AT	AT	AA	AT	AT	AA	AT	AT		
chr4	119469285	CC	CC	CT	CC	CT	CT	CC	CT	CT	CC	TT	CT		
chr4	123513165	CC	CC	CT	CT	CC	CT	CT	CC	CT	CT	CC	CT		
chr4	126954649	GG	GG	GA	GG	GA	GA	GG	GA	GA	GG	GA	GA		
chr4	128850953	CC	CC	CT	CC	CC	CT	CC	CC	CT	CC	CC	CT	True Positive	
chr4	137522218	GG	GG	GA	GG	GA	GA	GG	GA	GA	GG	GA	GA		
chr4	138761505	GG	GG	GT	GG	GT	GT	GG	GT	GT	GG	GT	GT		
chr4	143547351	GG	GG	GA	GA	GG	GA	GG	GG	GA	AA	GG	GA	False Positive	
chr4	149571503	AA	AA	AG	AA	AG	AG	AA	AG	AG	AA	AG	AG		
chr4	149571519	TT	TT	TG	TT	TG	TG	TT	TG	TG	TT	TG	TG		
chr4	166607350	GG	GG	GC	GG	GC	GC	GG	GC	GC	GG	GC	GC		
chr4	170594164	AA	AA	AG	AG	AA	AG	AG	AA	AG	AG	AA	AG		
chr4	171662331	CC	CC	CT	CT	CC	CT	CT	CC	CT	CT	CC	CT		
chr4	188366288	GG	GG	GC	GC	GG	GC	GC	GG	GC	GC	GG	GC		
chr4	192811178	CC	CC	CG	CG	CC	CG	CG	CC	CG	CG	CC	CG		
chr5	267405	CC	CC	CG	CG	CC	CG	CG	CC	CG	CG	CC	CG		
chr5	267410	TT	TT	TC	TC	TT	TC	TC	TT	TC	TC	TT	TC		
chr5	1111825	TT	TT	TC	TC	TT	TC	TC	TT	TC	TC	TT	TC		
chr5	5390179	GG	GG	GA	GG	GA	GA	GG	GA	GA	GG	GA	GA		
chr5	10622170	AA	AA	AG	AG	AA	AG	AG	AA	AG	AG	AA	AG		
chr5	16416847	GG	GG	GA	GG	GA	GA	GG	GA	GA	GG	GA	GA		
chr5	17087572	TT	TT	TC	TC	TT	TC	TC	TT	TC	TC	TT	TC		
chr5	17087576	CC	CC	CA	CA	CC	CA	CA	CC	CA	CA	CC	CA		
chr5	17328968	AA	AA	AG	AA	AG	AG	AA	AG	AG	AA	AG	AG		
chr5	39134220	GG	GG	GA	GG	GA	GA	GG	GA	GA	GG	GA	GA		
chr5	52435779	AA	AA	AT	AA	AT	AT	AA	AT	AT	AA	AT	AT		
chr5	63218837	GG	GG	GA	GG	GA	GA	GG	GA	GA	GG	GA	GA		
chr5	71561935	AA	AA	AG	AA	AG	AG	AA	AG	AG	AA	AG	AG		
chr5	76657514	GG	GG	GC	GG	GC	GC	GG	GC	GC	GG	GC	GC		
chr5	78518321	AA	AA	AG	AG	AA	AG	AG	AA	AG	AG	AA	AG		
chr5	93224710	AA	AA	AG	AG	AA	AG	AG	AA	AG	AG	AA	AG		
chr5	93480078	AA	AA	AG	AA	AA	AG	AA	AA	AG	AA	AA	AG	True Positive	
chr5	100685047	CC	CC	CA	CA	CC	CA	CA	CC	CA	CA	CC	CA		Seg Dup
chr5	104098061	CC	CC	CT	CC	CT	CT	CC	CT	CT	CC	CT	CT		
chr5	111357192	TT	TT	TC	TT	TC	TC	TT	TC	TC	TT	TC	TC		
chr5	117081488	AA	AA	AG	AA	AG	AG	AA	AG	AG	AA	AG	AG		
chr5	126993986	GG	GG	GA	GA	GG	GA	GG	GG	GA	AA	GG	GA	False Positive	
chr5	127584523	GG	GT	GT	GG	GG	GT	GG	GT	GT	GG	TT	GT		
chr5	142385748	AA	AA	AT	AA	AT	AT	AA	AT	AT	AA	AT	AT		
chr5	144330507	GG	GG	GT	GG	GG	GT	GG	GG	GT	GG	GG	GT	True Positive	
chr5	151380638	AA	AA	AC	AA	AC	AC	AA	AC	AC	AA	AC	AC		
chr5	160880210	CC	CC	CG	CC	CG	CG	CC	CG	CG	CC	CG	CG		
chr5	169958328	TT	TT	TC	TC	TT	TC	TC	TT	TC	TC	TT	TC		
chr5	175620435	GG	GG	GA	GA	GG	GA	GA	GG	GA	GA	GG	GA		
chr5	177198947	AA	AA	AG	AG	AA	AG	AG	AA	AG	AG	AA	AG		
chr5	179997523	GG	GG	GC	GG	GC	GC	GG	GC	GC	GG	GC	GC		
chr5	180131919	CC	CC	CT	CC	CC	CT	CC	CT	CT	CC	TT	CT		
chr5	180249324	CC	CC	CT	CC	CT	CT	CC	CT	CT	CC	CT	CT		
chr5	181062037	CC	CC	CT	CC	CT	CT	CC	CT	CT	CC	CT	CT		
chr6	3196156	TT	TT	TC	TC	TT	TC	TC	TT	TC	TC	TT	TC		
chr6	7711997	AG	AA	AT	AG	AA	AT	AG	AA	AT	AG	AA	AT	False Positive	
chr6	8968275	AA	AA	AC	AA	AC	AC	AA	AC	AC	AA	AC	AC		
chr6	10791867	TT	TT	TA	TA	TT	TA	TA	TT	TA	AA	TT	TA		
chr6	12022852	GG	GG	GT	GG	GG	GT	GG	GG	GT	GG	GG	GT	False Positive	
chr6	15099024	GA	GG	GG	GG	GG	GA	GA	GG	GA	GA	GG	GA		
chr6	15493940	CC	CC	CT	CT	CC	CT	CT	CC	CT	CT	CC	CT		
chr6	21435430	TT	TT	TC	TT	TC	TC	TT	TC	TC	TT	TC	TC		
chr6	21947057	TG	TT	TG	TT	TT	TG	TG	TT	TG	TG	TT	TG		
chr6	29851858	AA	AA	AC	AC	AA	AC	AC	AA	AC	AC	AA	AC		
chr6	30298972	TT	TT	TA	TT	TA	TA	TT	TA	TA	TT	TA	TA		
chr6	30321699	CC	CC	CA	CC	CA	CA	CC	CA	CA	CC	CA	CA		Seg Dup
chr6	31466061	CC	CC	CG	CC	CG	CG	CC	CG	CG	CC	CG	CG		
chr6	31641699	TT	TT	TG	TT	TG	TG	TT	TG	TG	TT	TG	TG		
chr6	31642840	GG	GG	GA	GG	GA	GA	GG	GA	GA	GG	GA	GA		
chr6	31645133	AA	AA	AG	AA	AG	AG	AA	AG	AG	AA	AG	AG		
chr6	31645135	TT	TT	TC	TT	TC	TC	TT	TC	TC	TT	TC	TC		
chr6	31654495	CC	CC	CT	CC	CT	CT	CC	CT	CT	CC	CT	CT		
chr6	32793314	CC	CC	CG	CG	CG	CG	CG	CG	CG	CG	CG	CG		
chr6	32793320	CC	CC	CT	CT	CT	CT	CT	CT	CT	CT	CT	CT		
chr6	32861238	CC	CC	CT	CT	CT	CT	CT	CT	CT	CT	CT	CT		
chr6	33247577	GG	GG	GC	GG	GC	GC	GG	GC	GC	GG	GC	GC		
chr6	33261071	TT	TT	TA	TT	TT	TA	TT	TT	TA	TT	TT	TA	False Positive	
chr6	38244821	GG	GG	GA	GG	GA	GA	GG	GA	GA	GG	GA	GA		
chr6	38315403	TT	TT	TC	TT	TC	TC	TT	TC	TC	TT	TC	TC		
chr6	39115484	AA	AA	AG	AG	AA	AG	AG	AA	AG	AG	AA	AG		
chr6	41134258	AA	AA	AC	AA	AC	AC	AA	AC	AC	AA	AC	AC		
chr6	57655685	AA	AA	AG	AG	AA	AG	AG	AA	AG	AG	AA	AG		

chr6	57655688	TT	TT	TC	TC	TT	TC	TC	TT	TC	TC	TT	TC		
chr6	57655696	AA	AA	AG	AG	AA	AG	AG	AA	AG	AG	AA	AG		
chr6	57788180	CC	CC	CT	CC	CC	CT	CC	CC	CT	CC	CC	CT	True Positive	
chr6	59270500	CC	CC	CT	CC	CT	CT	CT	CT	CT	CT	CT	CT		
chr6	65811488	GG	GG	GA	GG	GA	GA	GG	GA	GA	GG	GA	GA		Seg Dup
chr6	67376673	AA	AA	AT	AA	AT	AT	AA	AT	AT	AA	AT	AT		
chr6	68971246	GG	GG	GT	GG	GG	GT	GG	GG	GT	GG	GG	GT	True Positive	
chr6	70841670	CC	CC	CG	CG	CC	CG	CG	CC	CG	GG	CC	CG		
chr6	75551933	GG	GG	GA	GG	GA	GA	GG	GA	GA	GG	GA	GA		
chr6	76734113	TT	TT	TA	TT	TA	TA	TT	TA	TA	TT	TA	TA		
chr6	77411315	CC	CC	CT	CC	CT	CT	CC	CT	CT	CC	TT	CT		
chr6	91977192	GG	GG	GA	GG	GA	GA	GG	GA	GA	GG	GA	GA		
chr6	108049262	TG	TT	TG	TT	TT	TG	TG	TT	TG	TG	TT	TG		
chr6	115976437	AA	AA	AT	AA	AT	AT	AA	AT	AT	AA	AT	AT		
chr6	115976438	TT	TT	TG	TT	TG	TG	TT	TG	TG	TT	TG	TG		
chr6	130931815	CC	CC	CG	CC	CC	CG	CC	CC	CG	CC	CC	CG	True Positive	
chr6	159392087	GG	GG	GA	GA	GG	GA	GA	GG	GA	GA	GG	GA		
chr6	168519929	AA	AA	AG	AA	AG	AG	AA	AG	AG	AA	AG	AG		
chr6	169143913	CC	CC	CT	CC	CT	CT	CC	CT	CT	CC	CT	CT		
chr6	169252980	TT	TT	TA	TT	TA	TA	TT	TA	TA	TT	TA	TA		
chr6	169821482	GG	GG	GA	GA	GG	GA	GA	GG	GA	GA	GG	GA		
chr6	171467720	GG	GG	GA	GG	GA	GA	GG	GA	GA	GG	GA	GA		
chr6	171543928	AA	AA	AG	AA	AG	AG	AA	AG	AG	AA	AG	AG		
chr6	171731612	GG	GG	GC	GG	GC	GC	GG	GC	GC	GG	GC	GC		
chr7	590369	AA	AA	AG	AA	AG	AG	AA	AG	AG	AA	AG	AG		
chr7	1058638	GG	GG	GT	GT	GG	GT	GT	GG	GT	GT	GG	GT		
chr7	4826702	GG	GG	GC	GG	GC	GC	GG	GC	GC	GG	GC	GC		
chr7	5010087	TT	TT	TC	TT	TC	TC	TT	TC	TC	TT	TC	TC		
chr7	6736694	AG	AA	AG	AA	AA	AG	AG	AA	AG	GG	AA	AG		
chr7	6933999	CC	CC	CT	CT	CC	CT	CT	CC	CT	CT	CC	CT		
chr7	16571116	CC	CC	CG	CG	CC	CG	CG	CC	CG	CG	CC	CG		
chr7	28256479	CC	CC	CT	CC	CT	CT	CC	CT	CT	CC	CT	CT		
chr7	31369913	CC	CC	CG	CG	CC	CG	CG	CC	CG	CG	CC	CG		
chr7	36832008	GG	GG	GA	GA	GG	GA	GA	GG	GA	GA	GG	GA		
chr7	36921550	AA	AA	AG	AG	AA	AG	AG	AA	AG	AG	AA	AG		
chr7	37739352	AA	AA	AC	AA	AC	AC	AA	AC	AC	AA	AC	AC		
chr7	47507768	AA	AA	AG	AA	AG	AG	AA	AG	AG	AA	AG	AG		Seg Dup
chr7	61752013	GG	GG	GA	GG	GA	GA	GG	GA	GA	GG	GA	GA		
chr7	76137758	GG	GG	GA	GG	GA	GA	GG	GA	GA	GG	GA	GA		
chr7	76213998	GG	GG	GA	GG	GA	GA	GG	GA	GA	GG	GA	GA		Seg Dup
chr7	98509650	GG	GG	GA	GG	GA	GA	GG	GA	GA	GG	GA	GA		Seg Dup
chr7	98543541	TT	TT	TC	TC	TT	TC	TC	TT	TC	TC	TT	TC		
chr7	98708171	CC	CC	CA	CC	CA	CA	CC	CA	CA	CC	CA	CA		
chr7	108874137	GG	GG	GC	GG	GC	GC	GG	GC	GC	GG	GC	GC		
chr7	132198688	CC	CC	CT	CC	CT	CT	CC	CT	CT	CC	CT	CT		
chr7	133640551	GG	GG	GA	GA	GG	GA	GA	GG	GA	GA	GG	GA		
chr7	139923213	AA	AA	AC	AC	AA	AC	AC	AA	AC	AC	AA	AC		
chr7	140963085	CC	CC	CA	CC	CA	CA	CC	CA	CA	CC	CA	CA		
chr7	143874712	CC	CC	CT	CT	CC	CT	CT	CC	CT	CT	CC	CT		
chr7	144263579	AA	AA	AG	AG	AA	AG	AG	AA	AG	AG	AA	AG		
chr7	150920370	CC	CC	CA	CA	CC	CA	CA	CC	CA	CA	CC	CA		
chr7	151017850	AA	AA	AG	AA	AG	AG	AA	AG	AG	AA	AG	AG		
chr7	151122118	TT	TT	TC	TT	TC	TC	TT	TC	TC	TT	TC	TC		
chr7	151194008	AC	AA	AC	AA	AA	AC	AC	AA	AC	AC	AA	AC		
chr7	151426860	GG	GG	GA	GG	GA	GA	GG	GA	GA	GG	GA	GA		Seg Dup
chr7	155226938	CC	CC	CA	CC	CA	CA	CC	CA	CA	CC	CA	CA		
chr7	155721580	GG	GG	GT	GG	GT	GT	GG	GT	GT	GG	GT	GT		
chr7	157157064	GG	GG	GA	GG	GA	GA	GG	GA	GA	GG	GA	GA		
chr8	790329	CC	CC	CT	CT	CC	CT	CT	CC	CT	CT	CC	CT		
chr8	1322664	GC	GC	GC	GC	GA	GC	GA	GA	GC	GC	GA	GC	ND	
chr8	5346021	CT	CC	CG	CT	CC	CT	CT	CC	CT	CT	CC	CT		
chr8	8013315	CC	CC	CT	CC	CT	CT	CC	CT	CT	CC	CT	CT		Seg Dup
chr8	8166853	CC	CC	CT	CT	CT	CT	CT	CT	CT	CT	CT	CT		Seg Dup
chr8	8562245	CA	CC	CA	CC	CC	CA	CA	CC	CA	CA	CC	CA		Seg Dup
chr8	11773894	TT	TT	TA	TT	TA	TA	TT	TA	TA	TT	TA	TA		
chr8	12900631	AA	AA	AC	AC	AA	AC	AC	AA	AC	AC	AA	AC		
chr8	16568611	GG	GG	GA	GG	GG	GA	GG	GG	GA	GG	GG	GA	True Positive	
chr8	18844158	AA	AA	AG	AA	AG	AG	AA	AG	AG	AA	AG	AG		
chr8	19324576	AA	AA	AC	AA	AC	AC	AA	AC	AC	AA	AC	AC		
chr8	20026040	CC	CC	CT	CC	CT	CT	CC	CT	CT	CC	CT	CT		
chr8	37041261	GG	GG	GA	GG	GG	GA	GG	GG	GA	GG	GG	GA	True Positive	
chr8	45540246	GG	GG	GA	GG	GA	GA	GG	GA	GA	GG	AA	GA		
chr8	45716824	TT	TT	TA	TT	TA	TA	TT	TA	TA	TT	AA	TA		
chr8	49567692	AA	AA	AG	AG	AA	AG	AG	AA	AG	AG	AA	AG		
chr8	65944369	CC	CC	CT	CC	CC	CT	CC	CC	CT	CC	CC	CT	True Positive	
chr8	76204547	CC	CC	CA	CC	CA	CA	CC	CA	CA	CC	AA	CA		
chr8	79811674	GG	GG	GA	GG	GA	GA	GG	GA	GA	GG	GA	GA		
chr8	96111893	CC	CC	CT	CC	CT	CT	CC	CT	CT	CC	CT	CT		
chr8	126475877	TT	TT	TG	TT	TG	TG	TT	TG	TG	TT	TG	TG		
chr8	139065332	CC	CC	CA	CA	CC	CA	CA	CC	CA	CA	CC	CA		
chr8	141272994	TT	TT	TC	TT	TC	TC	TT	TC	TC	TT	TC	TC		
chr8	142414336	GG	GG	GA	GG	GG	GA	GG	GG	GA	GG	GG	GA	True Positive	
chr9	585340	CC	CC	CT	CC	CT	CT	CC	CT	CT	CC	CT	CT		
chr9	973934	GG	GG	GA	GG	GA	GA	GG	GA	GA	GG	GA	GA		
chr9	979556	CC	CC	CA	CC	CA	CA	CC	CA	CA	CC	CA	CA		
chr9	4831855	CC	CC	CT	CC	CT	CT	CC	CT	CT	CC	CT	CT		
chr9	5266982	AA	AA	AT	AA	AT	AT	AA	AT	AT	AA	TT	AT		
chr9	6270186	CC	CC	CT	CC	CT	CT	CC	CT	CT	CC	CT	CT		
chr9	6488614	AA	AA	AC	AA	AC	AC	AA	AC	AC	AA	AC	AC		
chr9	11127144	AA	AA	AC	AA	AC	AC	AA	AC	AC	AA	AC	AC		
chr9	73515869	CC	CC	CT	CC	CT	CT	CC	CT	CT	CC	CT	CT		
chr9	74463235	CC	CC	CT	CC	CC	CT	CC	CT	CT	CC	TT	CT		
chr9	78274091	TT	TT	TC	TC	TT	TC	TC	TT	TC	TC	TT	TC		
chr9	79669706	GG	GG	GT	GT	GG	GT	GT	GG	GT	GT	GG	GT		
chr9	79669708	GG	GG	GT	GT	GG	GT	GT	GG	GT	GT	GG	GT		
chr9	79669710	GG	GG	GT	GT	GG	GT	GT	GG	GT	GT	GG	GT		
chr9	82515767	TT	TT	TA	TT	TA	TA	TT	TA	TA	TT	TA	TA		Seg Dup
chr9	86736917	CC	CC	CT	CC	CC	CT	CC	CT	CT	CC	CT	CT		
chr9	86742315	TT	TT	TC	TT	TC	TC	TT	TC	TC	TT	TC	TC		
chr9	92771098	GG	GG	GA	GG	GA	GA	GG	GA	GA	GG	GA	GA		

chr9	92850386	AA	AA	AG	AA	AG	AG	AA	AG	AG	AA	AG	AG		
chr9	92850396	TT	TT	TC	TT	TC	TC	TT	TC	TC	TT	TC	TC		
chr9	93156888	TT	TT	TG	TG	TT	TG	TG	TT	TG	TG	TT	TG		Seg Dup
chr9	97525596	GG	GG	GA	GG	GA	GA	GG	GA	GA	GG	GA	GA		
chr9	101472059	TT	TT	TC	TT	TC	TC	TT	TC	TC	TT	TC	TC		
chr9	106316348	TT	TT	TC	TT	TC	TC	TT	TC	TC	TT	TC	TC		
chr9	107853343	GG	GG	GC	GG	GC	GC	GG	GC	GC	GG	GC	GC		
chr9	127393689	AA	AA	AG	AA	AG	AG	AA	AG	AG	AA	AG	AG		
chr9	128120964	AA	AA	AG	AA	AG	AG	AA	AG	AG	AA	AG	AG		
chr9	133326636	GG	GG	GA	GG	GA	GA	GG	GA	GA	GG	GA	GA		
chr9	133566450	AA	AA	AC	AA	AC	AC	AA	AC	AC	AA	AC	AC		
chr9	133652965	AA	AA	AG	AA	AG	AG	AA	AG	AG	AA	AG	AG		
chr9	134447215	AA	AA	AT	AA	AT	AT	AA	AT	AT	AA	AT	AT		
chr9	135725086	TT	TT	TC	TT	TC	TC	TT	TC	TC	TT	TC	TC		
chr9	136652212	GG	GG	GA	GG	GA	GA	GG	GA	GA	GG	GA	GA		
chr10	610446	CC	CC	CT	CT	CC	CT	CT	CC	CT	CT	CC	CT		
chr10	1650139	AA	AA	AG	AA	AG	AG	AA	AG	AG	AA	AG	AG		
chr10	8146481	CC	CC	CT	CC	CT	CT	CC	CT	CT	CC	CT	CT		
chr10	12003296	GG	GG	GA	GG	GA	GA	GG	GA	GA	GG	GA	GA		
chr10	13331290	CC	CC	CT	CC	CT	CT	CC	CT	CT	CC	CT	CT		
chr10	26872486	AA	AA	AT	AT	AA	AT	AT	AA	AT	AT	AA	AT		
chr10	30014112	AA	AA	AC	AC	AA	AC	AC	AA	AC	AC	AA	AC		
chr10	43041057	AA	AA	AC	AA	AC	AC	AA	AC	AC	AA	AC	AC		
chr10	46725782	AA	AA	AT	AA	AT	AT	AA	AT	AT	AA	AT	AT		Seg Dup
chr10	55406501	CC	CC	CA	CC	CA	CA	CC	CA	CA	CC	CA	CA		
chr10	70030903	GG	GG	GA	GG	GA	GA	GG	GA	GA	GG	GA	GA		
chr10	70760666	AA	AA	AC	AC	AA	AC	AC	AA	AC	AC	AA	AC		
chr10	77347545	AA	AA	AG	AA	AG	AG	AA	AG	AG	AA	AG	AG		
chr10	77695190	GG	GG	GA	GG	GA	GA	GG	GA	GA	GG	GA	GA		
chr10	95772758	GG	GG	GA	GA	GG	GA	GA	GG	GA	GA	GG	GA		
chr10	99928565	GG	GG	GA	GG	GA	GA	GG	GA	GA	GG	GA	GA		
chr10	107414901	CC	CC	CT	CT	CC	CT	CT	CC	CT	CT	CC	CT		
chr10	116597093	CC	CC	CT	CC	CT	CT	CC	CT	CT	CC	CT	CT		
chr10	117201920	TT	TT	TC	TT	TC	TC	TT	TC	TC	TT	TC	TC		
chr10	124461136	TT	TT	TG	TT	TG	TG	TT	TG	TG	TT	TG	TG		
chr10	127030503	GG	GG	GA	GA	GG	GA	GA	GG	GA	GA	GG	GA		
chr10	132253895	GG	GG	GC	GG	GC	GC	GG	GC	GC	GG	GC	GC		
chr10	133145513	AA	AA	AG	AA	AG	AG	AA	AG	AG	AA	AG	AG		
chr11	419706	AA	AA	AG	AA	AG	AG	AA	AG	AG	AA	AG	AG		
chr11	582527	CC	CC	CT	CC	CT	CT	CC	CT	CT	CC	CT	CT		
chr11	594473	GG	GG	GA	GG	GG	GA	GG	GA	GA	GG	GA	GA		
chr11	1150206	AA	AA	AG	AA	AG	AG	AA	AG	AG	AA	AG	AG		
chr11	6242494	AA	AA	AG	AG	AA	AG	AG	AA	AG	AG	AA	AG		
chr11	7239590	AA	AA	AG	AA	AA	AG	AA	AA	AG	AA	AA	AG	True Positive	
chr11	11351761	AA	AA	AG	AG	AA	AG	AG	AA	AG	AG	AA	AG		
chr11	15403418	GG	GG	GA	GG	GA	GA	GG	GA	GA	GG	GA	GA		
chr11	32363184	CC	CC	CA	CC	CA	CA	CC	CA	CA	CC	CA	CA		
chr11	36199420	TT	TT	TC	TT	TC	TC	TT	TC	TC	TT	TC	TC		
chr11	37561026	CC	CC	CT	CC	CT	CT	CC	CT	CT	CC	CT	CT		
chr11	38783344	AA	AA	AG	AA	AG	AG	AA	AG	AG	AA	AG	AG		
chr11	47460990	CC	CC	CT	CC	CT	CT	CC	CT	CT	CC	CT	CT		
chr11	47646608	TT	TT	TA	TT	TA	TA	TT	TA	TA	TT	TA	TA		
chr11	48644923	GG	GG	GT	GT	GG	GT	GT	GG	GT	GT	GG	GT		Seg Dup
chr11	60460356	CC	CC	CT	CT	CC	CT	CT	CC	CT	CT	CC	CT		
chr11	61015583	TT	TT	TC	TC	TC	TC	TC	TC	TC	TC	TC	TC		Seg Dup
chr11	68805182	GG	GG	GA	GG	GA	GA	GG	GA	GA	GG	GA	GA		
chr11	69915491	CC	CC	CG	CC	CG	CG	CC	CG	CG	CC	CG	CG		
chr11	70886410	GG	GG	GA	GG	GA	GA	GG	GA	GA	GG	GA	GA		
chr11	74490766	GT	GG	GT	GG	GG	GT	GT	GG	GT	GT	GG	GT		
chr11	78314817	AA	AA	AG	AG	AA	AG	AG	AA	AG	AG	AA	AG		
chr11	78314819	TT	TT	TC	TC	TT	TC	TC	TT	TC	TC	TT	TC		
chr11	78904336	CC	CC	CT	CT	CC	CT	CC	CC	CT	TT	CC	CT	ND	
chr11	87738201	GG	GG	GA	GA	GG	GA	GA	GG	GA	GA	GG	GA		Seg Dup
chr11	87851659	AA	AA	AT	AT	AA	AT	AT	AA	AT	AT	AA	AT		Seg Dup
chr11	87964125	AA	AA	AC	AC	AA	AC	AC	AA	AC	AC	AA	AC		
chr11	113195958	GG	GG	GT	GT	GG	GT	GT	GG	GT	GT	GG	GT		
chr11	113196015	CC	CC	CT	CT	CC	CT	CT	CC	CT	CT	CC	CT		
chr11	116939417	AA	AA	AT	AT	AA	AT	AT	AA	AT	AT	AA	AT		
chr11	120939906	TT	TT	TG	TG	TT	TG	TG	TT	TG	TG	TT	TG		
chr11	121072436	CC	CC	CT	CC	CT	CT	CC	CT	CT	CC	TT	CT		
chr11	121072437	AA	AA	AG	AA	AG	AG	AA	AG	AG	AA	GG	AG		
chr11	124137239	CC	CC	CG	CC	CG	CG	CC	CG	CG	CC	CG	CG		
chr11	129317577	CC	CC	CT	CT	CC	CT	CT	CC	CT	CT	CC	CT		
chr11	132557936	CC	CC	CA	CC	CA	CA	CC	CA	CA	CC	CA	CA		
chr12	1606481	GG	GG	GA	GG	GG	GA	GG	GA	GA	GG	AA	GA		
chr12	1786249	GG	GG	GT	GG	GG	GT	GG	GG	GT	GG	GG	GT	ND	
chr12	3120535	CC	CC	CG	CC	CG	CG	CC	CG	CG	CC	CG	CG		
chr12	6331509	GG	GG	GA	GG	GA	GA	GG	GA	GA	GG	GA	GA		Seg Dup
chr12	6637216	GG	GG	GT	GG	GT	GT	GG	GT	GT	GG	GT	GT		
chr12	6727719	CC	CC	CT	CC	CT	CT	CC	CT	CT	CC	CT	CT		
chr12	8148258	CC	CC	CT	CC	CT	CT	CC	CT	CT	CC	CT	CT		
chr12	9549332	TT	TT	TG	TG	TG	TG	TG	TG	TG	TG	TG	TG		
chr12	9714865	GG	GG	GA	GG	GA	GA	GG	GA	GA	GG	GA	GA		Seg Dup
chr12	14055837	TT	TT	TA	TT	TT	TA	TT	TT	TA	TT	TT	TA	False Positive	
chr12	15380511	AA	AA	AT	AA	AT	AT	AA	AT	AT	AA	AT	AT		
chr12	16874642	CC	CC	CA	CC	CC	CA	CC	CC	CA	CC	CC	CA	True Positive	
chr12	28800658	CC	CC	CT	CC	CC	CT	CC	CC	CT	CC	CC	CT	False Positive	
chr12	34025746	AA	AA	AG	AA	AG	AG	AA	AG	AG	AA	AG	AG		
chr12	38219656	CC	CC	CT	CC	CT	CT	CC	CT	CT	CC	TT	CT		
chr12	38587178	AA	AA	AT	AA	AT	AT	AA	AT	AT	AA	TT	AT		
chr12	40018151	CC	CC	CA	CC	CA	CA	CC	CA	CA	CC	CA	CA		
chr12	47872130	TT	TT	TC	TT	TC	TC	TT	TC	TC	TT	TC	TC		
chr12	47872131	CC	CC	CT	CC	CT	CT	CC	CT	CT	CC	CT	CT		
chr12	48465487	AA	AA	AT	AA	AT	AT	AA	AT	AT	AA	AT	AT		
chr12	48995740	AA	AA	AC	AC	AA	AC	AC	AA	AC	AC	AA	AC		
chr12	48999489	AA	AA	AG	AA	AA	AG	AA	AA	AG	AA	AA	AG	ND	
chr12	54928551	AA	AA	AG	AA	AG	AG	AA	AG	AG	AA	AG	AG		
chr12	56012842	CC	CC	CG	CC	CG	CG	CC	CG	CG	CC	CG	CG		
chr12	56144043	AA	AA	AG	AA	AG	AG	AA	AG	AG	AA	AG	AG		
chr12	57402389	AA	AA	AG	AA	AG	AG	AA	AG	AG	AA	AG	AG		

chr12	59518267	AA	AA	AC	AA	AC	AC	AA	AC	AC	AA	AC	AC		
chr12	69672324	TT	TT	TA	TT	TA	TA	TT	TA	TA	TT	AA	TA		
chr12	74028360	TT	TT	TA	TT	TT	TA	TT	TT	TA	TT	TT	TA	True Positive	
chr12	77338821	AG	AA	AG	AA	AA	AG	AG	AA	AG	AG	AA	AG		
chr12	77392034	AA	AA	AG	AA	AG	AG	AA	AG	AG	AA	AG	AG		
chr12	87522336	TT	TT	TC	TT	TT	TC	TT	TT	TC	TT	TT	TC	True Positive	
chr12	93785227	TT	TT	TC	TT	TC	TC	TT	TC	TC	TT	TC	TC		
chr12	96475722	AA	AA	AG	AA	AG	AG	AA	AG	AG	AA	AG	AG		
chr12	100682880	CC	CC	CT	CC	CC	CT	CC	CC	CT	CC	CC	CT	True Positive	
chr12	102392564	GG	GG	GA	GG	GG	GA	GG	GG	GA	GG	GG	GA	True Positive	
chr12	107827085	TT	TT	TC	TT	TC	TC	TT	TC	TC	TT	TC	TC		
chr12	109508443	AA	AA	AG	AA	AG	AG	AA	AG	AG	AA	AG	AG		
chr12	118993125	TT	TT	TA	TA	TT	TA	TA	TT	TA	TA	TT	TA		
chr12	118993128	TT	TT	TA	TA	TT	TA	TA	TT	TA	TA	TT	TA		
chr12	128944118	CC	CC	CT	CC	CC	CT	CC	CC	CT	CC	CC	CT	ND	
chr12	128950382	GG	GG	GA	GG	GA	GA	GG	GA	GA	GG	GA	GA		
chr12	129242316	TT	TT	TC	TT	TC	TC	TT	TC	TC	TT	TC	TC		
chr12	130866602	AA	AA	AG	AA	AG	AG	AA	AG	AG	AA	AG	AG		
chr13	18728470	AA	AA	AG	AA	AG	AG	AA	AG	AG	AA	AG	AG		Seg Dup
chr13	20281162	GG	GG	GA	GG	GA	GA	GG	GA	GA	GG	GA	GA		
chr13	20858175	CC	CC	CT	CC	CT	CT	CC	CT	CT	CC	CT	CT		
chr13	23657352	GG	GG	GT	GG	GT	GT	GG	GT	GT	GG	GT	GT		
chr13	31766505	CC	CC	CA	CC	CA	CA	CC	CA	CA	CC	CA	CA		
chr13	37406099	AA	AA	AG	AG	AA	AG	AG	AA	AG	AG	AA	AG		
chr13	54827368	CC	CC	CA	CA	CC	CA	CA	CC	CA	CA	CC	CA		
chr13	60662417	TT	TT	TA	TA	TT	TA	TA	TT	TA	TA	TT	TA		
chr13	60662424	CC	CC	CT	CT	CC	CT	CT	CC	CT	CT	CC	CT		
chr13	98085305	AA	AA	TA	TA	TA	TA	TA	TA	TA	AA	AA	AA		
chr13	99006558	AA	AA	AG	AG	AA	AG	AG	AA	AG	AG	AA	AG		
chr13	110619775	AA	AA	AC	AC	AA	AC	AC	AA	AC	AC	AA	AC		
chr13	110619809	TT	TT	TC	TC	TT	TC	TC	TT	TC	TC	TT	TC		
chr13	110619847	AA	AA	AT	AT	AA	AT	AT	AA	AT	AT	AA	AT		
chr13	112907657	AT	AT	AG	AT	AT	AT	AT	AT	AT	AT	AT	AT		
chr13	113497649	CA	CC	CA	CC	CC	CA	CA	CC	CA	CA	CC	CA		
chr13	114968294	CC	CC	CG	CC	CG	CG	CC	CG	CG	CC	CG	CG		
chr14	20537600	TT	TT	TC	TC	TT	TC	TC	TT	TC	TC	TT	TC		
chr14	26827567	AA	AA	AT	AA	AA	AT	AA	AT	AT	AA	AT	AT		
chr14	29841267	CC	CC	CT	CT	CC	CT	CT	CC	CT	CT	CC	CT		
chr14	33207972	CC	CC	CT	CC	CT	CT	CC	CT	CT	CC	CT	CT		
chr14	40873347	GG	GG	GA	GA	GG	GA	GA	GG	GA	GA	GG	GA		
chr14	50102243	CC	CC	CT	CT	CC	CT	CT	CC	CT	CT	CC	CT		
chr14	54202067	AA	AA	AG	AA	AG	AG	AA	AG	AG	AA	AG	AG		
chr14	54607286	AA	AA	AG	AG	AA	AG	AG	AA	AG	AG	AA	AG		
chr14	57256678	TT	TT	TG	TT	TG	TG	TT	TG	TG	TT	TG	TG		
chr14	68448334	GG	GG	GC	GG	GC	GC	GG	GC	GC	GG	GC	GC		
chr14	68476430	AA	AA	AG	AG	AA	AG	AG	AA	AG	AG	AA	AG		
chr14	69270970	CC	CC	CT	CT	CC	CT	CT	CC	CT	CT	CC	CT		
chr14	69545496	CC	CC	CT	CT	CC	CT	CT	CC	CT	CT	CC	CT		
chr14	69545497	TT	TT	TA	TA	TT	TA	TA	TT	TA	TA	TT	TA		
chr14	73173751	GG	GG	GA	GG	GA	GA	GG	GA	GA	GG	GA	GA		
chr14	95367827	CC	CC	CG	CG	CC	CG	CG	CC	CG	CG	CC	CG		
chr14	98384649	AA	AA	AG	AA	AG	AG	AA	AG	AG	AA	AG	AG		
chr14	102515070	GG	GG	GC	GG	GG	GC	GG	GG	GC	GG	GG	GC	True Positive	
chr14	102541817	GG	GG	GT	GG	GG	GT	GG	GG	GT	GG	GG	GT	True Positive	
chr15	18429706	AA	AA	AG	AA	AG	AG	AA	AG	AG	AA	AG	AG		Seg Dup
chr15	18646000	TT	TT	TG	TT	TG	TG	TT	TG	TG	TT	TG	TG		Seg Dup
chr15	18646023	GG	GG	GA	GG	GG	GA	GG	GA	GA	GG	GA	GA		Seg Dup
chr15	20853117	GA	GG	GA	GG	GG	GA	GA	GG	GA	GA	GG	GA		Seg Dup
chr15	21679508	GG	GG	GC	GC	GG	GC	GC	GG	GC	GC	GG	GC		
chr15	21679711	AA	AA	AG	AG	AA	AG	AG	AA	AG	AG	AA	AG		
chr15	21679719	CC	CC	CG	CG	CC	CG	CG	CC	CG	CG	CC	CG		
chr15	21923529	GG	GG	GA	GA	GG	GA	GA	GG	GA	GA	GG	GA		Seg Dup
chr15	25041842	GG	GG	GA	GG	GA	GA	GG	GA	GA	GG	GA	GA		
chr15	25144315	AA	AA	AC	AC	AA	AC	AC	AA	AC	AC	AA	AC		
chr15	26213756	GG	GG	GT	GG	GT	GT	GG	GT	GT	GG	GT	GT		Seg Dup
chr15	30562289	CT	CC	CT	CC	CC	CT	CT	CC	CT	CT	CC	CT		
chr15	34818070	CC	CC	CT	CC	CC	CT	CC	CC	CT	CC	CC	CT	True Positive	
chr15	43021601	CC	CC	CA	CC	CA	CA	CC	CA	CA	CC	CA	CA		
chr15	47378530	GG	GG	GC	GC	GG	GC	GC	GG	GC	GC	GG	GC		
chr15	55635017	CC	CC	CT	CT	CC	CT	CT	CC	CT	CT	CC	CT		
chr15	56516232	GC	GG	GC	GG	GG	GC	GC	GG	GC	GC	GG	GC		
chr15	62786977	GG	GG	GA	GG	GG	GA	GG	GG	GA	GG	GG	GA	ND	
chr15	63251260	AA	AA	AC	AC	AA	AC	AC	AA	AC	AC	AA	AC		
chr15	70870371	CC	CC	CT	CC	CT	CT	CC	CT	CT	CC	CT	CT		
chr15	72388653	CC	CC	CA	CC	CA	CA	CC	CA	CA	CC	CA	CA		
chr15	73733158	CC	CC	CT	CC	CT	CT	CC	CT	CT	CC	CT	CT		
chr15	74261127	GA	GG	GA	GA	GG	GA	GG	GG	GA	AA	GG	GA	ND	
chr15	75475944	CC	CC	CG	CC	CG	CG	CC	CG	CG	CC	CG	CG		
chr15	75490996	CC	CC	CT	CC	CT	CT	CC	CT	CT	CC	CT	CT		
chr15	77065921	TT	TT	TC	TT	TC	TC	TT	TC	TC	TT	TC	TC		
chr15	79911449	AA	AA	AC	AA	AC	AC	AA	AC	AC	AA	AC	AC		
chr15	83078379	CC	CC	CT	CC	CC	CT	CC	CC	CT	CC	CC	CT	True Positive	
chr15	85601294	GG	GG	GA	GG	GA	GA	GG	GA	GA	GG	GA	GA		
chr15	87161787	AA	AA	AG	AA	AG	AG	AA	AG	AG	AA	AG	AG		
chr15	87604378	GG	GG	GC	GC	GG	GC	GC	GG	GC	GC	GG	GC		
chr15	93651117	CC	CC	CT	CC	CT	CT	CC	CT	CT	CC	CT	CT		
chr15	99212751	AA	AA	AG	AG	AA	AG	AG	AA	AG	AG	AA	AG		
chr16	114818	TT	TT	TC	TC	TT	TC	TC	TT	TC	TC	TT	TC		
chr16	311791	GG	GG	GA	GG	GA	GA	GG	GA	GA	GG	GA	GA		
chr16	1031024	AC	AC	AA	AC	AC	AG	AC	AC	AC	AC	AC	AC		
chr16	2679320	TT	TT	TC	TT	TC	TC	TT	TC	TC	TT	TC	TC		Seg Dup
chr16	2749033	TT	TT	TC	TT	TC	TC	TT	TC	TC	TT	TC	TC		Seg Dup
chr16	5562857	CC	CC	CT	CT	CC	CT	CT	CC	CT	CT	CC	CT		
chr16	5562858	AA	AA	AG	AG	AA	AG	AG	AA	AG	AG	AA	AG		
chr16	5562861	TT	TT	TG	TG	TT	TG	TG	TT	TG	TG	TT	TG		
chr16	21274520	TT	TT	TC	TT	TC	TC	TT	TC	TC	TT	TC	TC		Seg Dup
chr16	21897065	AA	AA	AT	AA	AT	AT	AA	AT	AT	AA	AT	AT		Seg Dup
chr16	27694055	AA	AA	AG	AA	AG	AG	AA	AG	AG	AA	AG	AG		
chr16	32567034	CC	CC	CT	CC	CT	CT	CC	CT	CT	CC	CT	CT		Seg Dup
chr16	51691257	TT	TT	TC	TC	TT	TC	TC	TT	TC	TC	TT	TC		

chr16	53974014	GG	GG	GC	GC	GG	GC	GC	GG	GC	GC	GG	GC	
chr16	55717100	CC	CC	CT	CT	CC	CT	CT	CC	CT	CT	CC	CT	
chr16	69163860	CC	CC	CG	CC	CG	CG	CC	CG	CG	CC	CG	CG	
chr16	75895991	AA	AA	AG	AA	AG	AG	AA	AG	AG	AA	AG	AG	
chr16	84731516	AA	AA	AG	AG	AA	AG	AG	AA	AG	AG	AA	AG	
chr16	84731519	AA	AA	AG	AG	AA	AG	AG	AA	AG	AG	AA	AG	
chr16	84731525	TT	TT	TC	TC	TT	TC	TC	TT	TC	TC	TT	TC	
chr16	87028571	TT	TT	TC	TT	TC	TC	TT	TC	TC	TT	TC	TC	
chr16	87317305	CC	CC	CG	CC	CG	CG	CC	CG	CG	CC	CG	CG	
chr16	87317315	AA	AA	AG	AA	AG	AG	AA	AG	AG	AA	AG	AG	
chr16	87621029	CC	CC	CT	CC	CT	CT	CC	CT	CT	CC	CT	CT	Seg Dup
chr17	992049	GG	GG	GT	GG	GT	GT	GG	GT	GT	GG	GT	GT	
chr17	1237113	CC	CC	CG	CC	CG	CG	CC	CG	CG	CC	CG	CG	
chr17	1237117	GG	GG	GT	GG	GT	GT	GG	GT	GT	GG	GT	GT	
chr17	1872432	CC	CC	CT	CC	CT	CT	CC	CT	CT	CC	CT	CT	
chr17	2017382	GG	GG	GT	GG	GT	GT	GG	GT	GT	GG	GT	GT	
chr17	10252837	CC	CC	CT	CC	CT	CT	CC	CT	CT	CC	CT	CT	
chr17	12832244	GG	GG	GA	GG	GA	GA	GG	GA	GA	GG	GA	GA	
chr17	18871320	GG	GG	GA	GA	GG	GA	GA	GG	GA	GA	GG	GA	Seg Dup
chr17	21421316	GG	GG	GT	GT	GG	GT	GT	GG	GT	GT	GG	GT	
chr17	26368078	CC	CC	CT	CC	CT	CT	CC	CT	CT	CC	CT	CT	
chr17	43609968	TT	TT	TG	TT	TG	TG	TT	TG	TG	TT	TG	TG	
chr17	45961834	AA	AA	AG	AA	AG	AG	AA	AG	AG	AA	AG	AG	
chr17	51923494	AA	AA	AG	AA	AG	AG	AA	AG	AG	AA	AG	AG	
chr17	52666591	GG	GG	GT	GG	GG	GT	GG	GG	GT	GG	GG	GT	True Positive
chr17	53592195	CC	CG	CT	CG	CG	CG	CG	CG	CG	CG	CG	CG	
chr17	62679106	AA	AA	AG	AG	AG	AG	AG	AG	AG	AG	AG	AG	Seg Dup
chr17	66446424	GG	GG	GT	GG	GG	GT	GG	GT	GT	GG	GT	GT	
chr17	72122479	CC	CC	CT	CC	CC	CT	CC	CT	CT	CC	TT	CT	
chr17	75468885	CC	CC	CT	CC	CT	CT	CC	CT	CT	CC	CT	CT	
chr17	79113031	GG	GG	GA	GA	GG	GA	GA	GG	GA	GA	GG	GA	
chr17	79817781	CC	CC	CG	CC	CG	CG	CC	CG	CG	CC	CG	CG	
chr17	80860323	GG	GG	GA	GG	GA	GA	GG	GA	GA	GG	GA	GA	
chr17	81431588	TT	TT	TG	TG	TT	TG	TG	TT	TG	TG	TT	TG	
chr17	82244360	TT	TT	TC	TT	TC	TC	TT	TC	TC	TT	TC	TC	
chr17	82614732	CC	CC	CA	CC	CA	CA	CC	CA	CA	CC	CA	CA	
chr18	7836474	CC	CC	CT	CC	CC	CT	CC	CC	CT	CC	CC	CT	True Positive
chr18	46319429	TT	TT	TC	TC	TT	TC	TC	TT	TC	TC	TT	TC	
chr18	53064484	TT	TT	TC	TT	TC	TC	TT	TC	TC	TT	TC	TC	
chr18	73836120	CC	CC	CT	CC	CT	CT	CC	CT	CT	CC	CT	CT	
chr18	75118883	TT	TT	TC	TT	TC	TC	TT	TC	TC	TT	TC	TC	
chr18	75774236	CC	CC	CT	CT	CC	CT	CT	CC	CT	CT	CC	CT	
chr19	571775	CC	CC	CT	CC	CT	CT	CC	CT	CT	CC	CT	CT	
chr19	872481	GG	GG	GT	GG	GT	GT	GG	GT	GT	GG	GT	GT	
chr19	1157370	CC	CC	CG	CC	CG	CG	CC	CG	CG	CC	CG	CG	
chr19	1474463	CC	CC	CT	CC	CC	CT	CC	CC	CT	CC	CC	CT	True Positive
chr19	1748679	GG	GG	GA	GG	GG	GA	GG	GG	GA	GG	GG	GA	True Positive
chr19	1980626	AA	AA	AG	AA	AG	AG	AA	AG	AG	AA	AG	AG	
chr19	6881835	AA	AA	AG	AG	AA	AG	AG	AA	AG	AG	AA	AG	
chr19	7330455	GG	GG	GA	GG	GA	GA	GG	GA	GA	GG	GA	GA	
chr19	10322292	CC	CC	CT	CC	CT	CT	CC	CT	CT	CC	CT	CT	
chr19	14713113	CC	CC	CT	CC	CT	CT	CC	CT	CT	CC	CT	CT	
chr19	14728685	AA	AA	AG	AA	AG	AG	AA	AG	AG	AA	AG	AG	
chr19	16289464	CC	CC	CT	CT	CC	CT	CT	CC	CT	CT	CC	CT	
chr19	20350475	GG	GG	GA	GG	GA	GA	GG	GA	GA	GG	AA	GA	
chr19	21309408	GG	GG	GT	GT	GG	GT	GT	GG	GT	GT	GG	GT	
chr19	32870884	CC	CC	CT	CC	CT	CT	CC	CT	CT	CC	CT	CT	
chr19	37588655	AA	AA	AC	AA	AC	AC	AA	AC	AC	AA	AC	AC	
chr19	37879905	AA	AA	AT	AA	AA	AT	AA	AA	AT	AA	AA	AT	True Positive
chr19	46579202	GG	GG	GC	GG	GC	GC	GG	GC	GC	GG	GC	GC	
chr19	47286006	GG	GG	GA	GG	GA	GA	GG	GA	GA	GG	GA	GA	
chr19	48415764	AA	AA	AT	AA	AT	AT	AA	AT	AT	AA	AT	AT	
chr19	48669585	CC	CC	CT	CC	CT	CT	CC	CT	CT	CC	CT	CT	
chr19	50835533	TT	TC	TC	TT	TC	TC	TT	TT	TC	TT	TC	TC	False Positive
chr19	52249179	TT	TT	TA	TT	TA	TA	TT	TA	TA	TT	TA	TA	
chr19	62777926	AA	AA	AG	AA	AG	AG	AA	AG	AG	AA	AG	AG	
chr20	1525119	AA	AA	AC	AA	AC	AC	AA	AC	AC	AA	AC	AC	
chr20	1856692	AA	AA	AG	AA	AG	AG	AA	AG	AG	AA	AG	AG	
chr20	1976411	AA	AA	AG	AG	AA	AG	AG	AA	AG	AG	AA	AG	
chr20	4563857	CC	CC	CT	CC	CC	CT	CC	CC	CT	CC	CC	CT	True Positive
chr20	12676790	TT	TT	TC	TT	TC	TC	TT	TC	TC	TT	TC	TC	
chr20	15631596	CC	CC	CT	CC	CC	CT	CC	CC	CT	CC	CC	CT	True Positive
chr20	21032505	TT	TT	TA	TT	TA	TA	TT	TA	TA	TT	TA	TA	
chr20	23925005	AA	AA	AC	AA	AC	AC	AA	AC	AC	AA	AC	AC	
chr20	24883667	CC	CC	CT	CT	CC	CT	CT	CC	CT	CT	CC	CT	
chr20	28735300	GG	GG	GA	GG	GA	GA	GG	GA	GA	GG	GA	GA	
chr20	29736370	AA	AA	AG	AG	AA	AG	AG	AA	AG	AG	AA	AG	
chr20	34399878	AA	AA	AC	AA	AC	AC	AA	AC	AC	AA	AC	AC	
chr20	42897988	TT	TT	TC	TT	TC	TC	TT	TC	TC	TT	TC	TC	
chr20	44855593	GG	GG	GA	GA	GG	GA	GA	GG	GA	GA	GG	GA	
chr20	46963971	CC	CC	CT	CC	CT	CT	CC	CT	CT	CC	CT	CT	
chr20	50562284	AA	AA	AG	AA	AG	AG	AA	AG	AG	AA	AG	AG	
chr20	50866230	GG	GG	GT	GG	GT	GT	GG	GT	GT	GG	GT	GT	
chr20	54369196	TT	TT	TA	TT	TA	TA	TT	TA	TA	TT	AA	TA	
chr20	57268503	AA	AA	AG	AG	AA	AG	AG	AA	AG	AG	AA	AG	
chr20	57268524	GG	GG	GA	GA	GG	GA	GA	GG	GA	GA	GG	GA	
chr20	57268525	GG	GG	GA	GA	GG	GA	GA	GG	GA	GA	GG	GA	
chr20	58217046	GG	GG	GA	GA	GG	GA	GA	GG	GA	GA	GG	GA	
chr20	61195081	CC	CC	CT	CC	CT	CT	CC	CT	CT	CC	CT	CT	
chr21	7080321	GG	GG	GT	GT	GG	GT	GT	GG	GT	GT	GG	GT	
chr21	7603292	AA	AA	AG	AA	AG	AG	AA	AG	AG	AA	AG	AG	
chr21	22590398	TT	TT	TC	TT	TC	TC	TT	TC	TC	TT	TC	TC	
chr21	24889038	CC	CC	CT	CC	CT	CT	CC	CT	CT	CC	CT	CT	
chr21	32092335	TT	TT	TC	TT	TT	TC	TT	TC	TC	TT	TC	TC	
chr21	32262516	TT	TT	TG	TT	TG	TG	TT	TG	TG	TT	TG	TG	
chr22	18410445	TT	TT	TC	TT	TC	TC	TT	TC	TC	TT	TC	TC	
chr22	18751518	CC	CC	CT	CC	CT	CT	CC	CT	CT	CC	CT	CT	
chr22	19368406	GG	GA	GA	GG	GG	GA	GG	GA	GA	GG	GA	GA	
chr22	19419088	AA	AA	AT	AA	AT	AT	AA	AT	AT	AA	AT	AT	Seg Dup
chr22	19779356	AA	AA	AG	AG	AG	AG	AG	AG	AG	AG	AG	AG	Seg Dup

chr22	20176576	**CC**	**CC**	**CT**	CT	CC	CT	CT	CC	CT	CT	CC	CT		
chr22	21192121	**AG**	**AG**	**AC**	**AG**	**AG**	**AC**	AG	AG	AG	AG	AG	AG		Seg Dup
chr22	21234171	**CC**	**CC**	**CT**	CC	CT	CT	CC	CT	CT	CC	CT	CT		
chr22	21639757	TC	TT	TC	**TT**	**TT**	**TC**	TC	TT	TC	TC	TT	TC		
chr22	21668255	**CC**	**CC**	**CG**	CG	CC	CG	CG	CC	CG	CG	CC	CG		
chr22	22079865	**TT**	**TT**	**TC**	**TT**	**TT**	**TC**	**TT**	**TT**	**TC**	**TT**	**TT**	**TC**	True Positive	
chr22	22163245	GA	GG	GA	**GC**	**GG**	**GA**	GA	GG	GA	**GC**	**GG**	**GA**	False Positive	Seg Dup
chr22	30021042	**AA**	**AA**	**AT**	AA	AT	AT	AA	AT	AT	AA	TT	AT		
chr22	36877764	**GG**	**GG**	**GA**	GA	GG	GA	GA	GG	GA	GA	GG	GA		
chr22	37248032	**TT**	**TT**	**TC**	TT	TC	TC	TT	TC	TC	TT	TC	TC		
chr22	41070369	**TT**	**TT**	**TC**	TT	TC	TC	TT	TC	TC	TT	TC	TC		
chr22	41592669	**GG**	**GG**	**GA**	**GG**	**GG**	**GA**	**GG**	**GG**	**GA**	**GG**	**GG**	**GA**	True Positive	
chr22	41842596	**CC**	**CC**	**CA**	CC	CA	CA	CC	CA	CA	CC	CA	CA		
chr22	42817472	**TT**	**TT**	**TC**	TT	TC	TC	TT	TC	TC	TT	TC	TC		
chr22	45494890	**TT**	**TT**	**TC**	TT	TC	TC	TT	TC	TC	TT	TC	TC		
chr22	47192375	**CC**	**CC**	**CG**	CG	CC	CG	CG	CC	CG	CG	CC	CG		
chr22	47192439	**AA**	**AA**	**AG**	AG	AA	AG	AG	AA	AG	AG	AA	AG		
chr22	48036929	**TT**	**TT**	**TC**	TT	TC	TC	TT	TC	TC	TT	TC	TC		
chr22	48036969	**CC**	**CC**	**CT**	CC	CT	CT	CC	CT	CT	CC	CT	CT		
chr22	48608294	**AA**	**AA**	**AG**	AG	AA	AG	AG	AA	AG	AG	AA	AG		
chr22	48646770	**GG**	**GG**	**GC**	GG	GC	GC	GG	GC	GC	GG	GC	GC		
chr22	49125261	**AA**	**AA**	**AG**	AA	AG	AG	AA	AG	AG	AA	AG	AG		
chr22	49209110	**CC**	**CC**	**CA**	CC	CA	CA	CC	CA	CA	CC	CA	CA		

Supplemental Method. Detailed pipeline for mapping and variant calling methods used in this study.

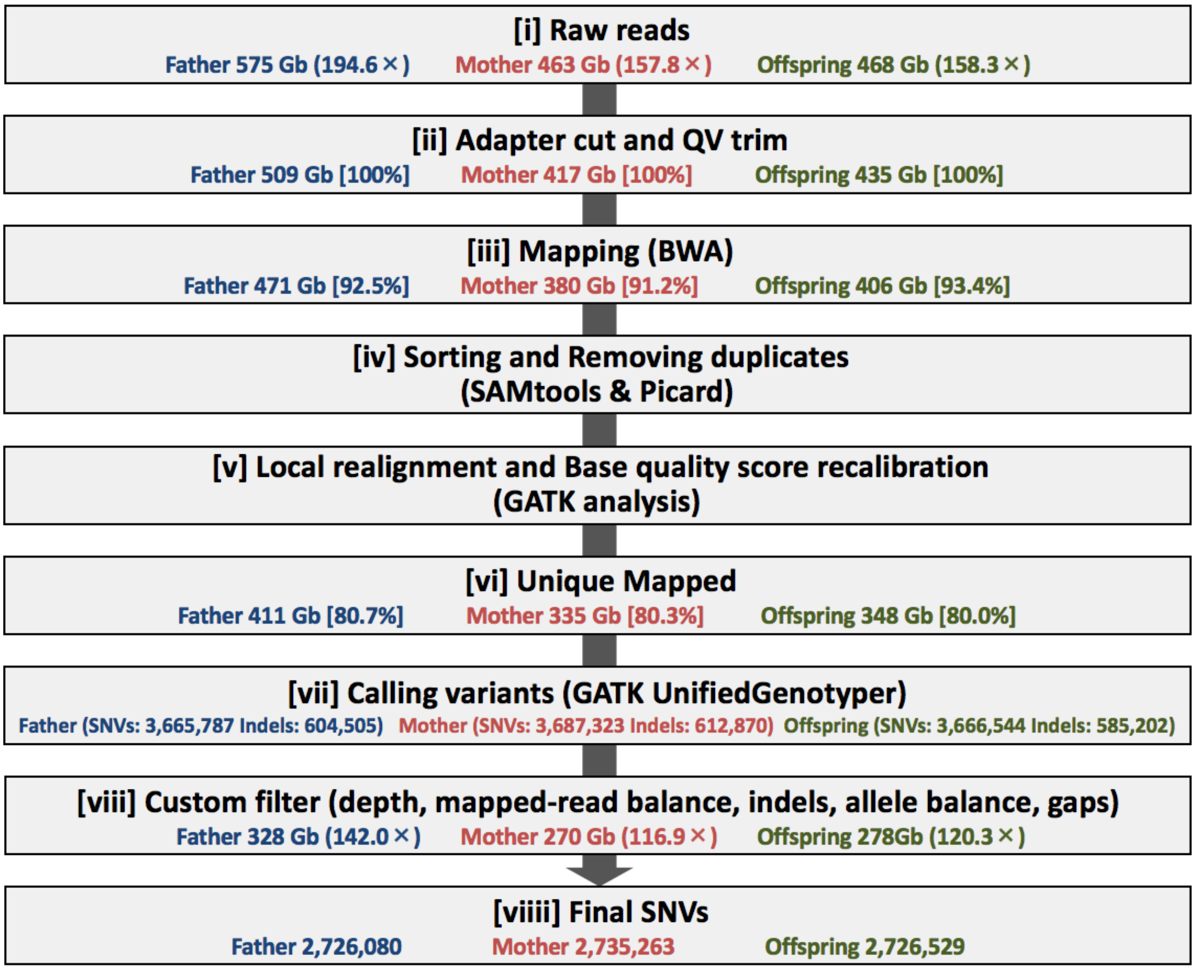

STEP [ii] Adapter cut and QV trim

Trim adapter sequences
Find adapter head sequence (default seed length is 6bp). Compare the adapter sequence and trim if they are matched.

command:
```
$ trimadapt.pl –I Mother.1.fastq -l 8 > Mother.1.trim
$ trimadapt.pl –I Mother.2.fastq –l 8 > Mother.2.trim
```

In house script option's;
-I : input sequence in fastq format
-a : adapter sequence in string [default : AGATCGGAAGAGCG]
-s : seed length (default 6)
-m : number of mismatch in overlap (default 1)
-l : minimum overlap length (default 10)
-b : minimum read length to report (default 16)
*-c : clip first base of adapter [*corresponding base in read for adapter's first base is also trimmed.]*
-n : count N/n in mismatch base (no count for default)
-d : trim 3' sequence if it matches for the sub sequence of the adapter.
-f : adapter sequence in fasta file

Trim Low-quality sequences
Low-quality sequences were defined by the averaged quality value (QV) < 20 for a given base ± 1 adjacent nucleotide and were marked. If a marked position was located at either the 5´ or 3´ end or both, these bases were trimmed.

Command:
```
$ cleanfastq –q 20 –w 3 –l 20 –f –s –o Mother.1.log Mother.1.trim >
  Mother.1.qv20.fq
$ cleanfastq –q 20 –w 3 –l 20 –f –s –o Mother.2.log Mother.2.trim >
  Mother.2.qv20.fq
```

Default Parameters are;
- Minimum Average Quality (minqual or –q) : 20
- Window Size (window or –w) : 3
- Minimum Read Length (minlen or –l) : 0
- "-s" : Sanger Quality
- "-f" : Fastq format
- "-o" : Output log file

STEP [iii] Mapping (BWA)

Reference:
Father, Offspring: PanTro2.1.4
Mother: PanTro2.1.4 (w/o chrY)

command:
```
$ bwa aln PanTro4rmY.fa Mother.1.qv20.fq > Mother.1.sai
$ bwa aln PanTro4rmY.fa Mother.2.qv20.fq > Mother.2.sai
$ bwa sampe PanTro4rmY.fa Mother.1.sai Mother.2.sai Mother.1.qv20.fq
  Mother.2.qv20.fq > Mother.sam
$ samtools view –bS Mother.sam > Mother.bam
$ samtools index Mother.bam
```

STEP [iv] Sorting and Removing duplicates (SAMtools & Picard)

command:
```
$ samtools sort Mother.bam Mother.sorted
$ java -Xmx4g -jar -XX:ParallelGCThreads=1 MarkDuplicates.jar
  REMOVE_DUPLICATES=true INPUT=Mother.sorted.bam OUTPUT=Mother.rmDUP.bam
  METRICS_FILE=Mother.rmDUP.txt VALIDATION_STRINGENCY=SILENT AS=true
$ samtools index Mother.rmDUP.bam
```

STEP [v] Local realignment and Base quality score recalibraion (GATK analysis)

command:
```
$ java -Xmx4g -jar GenomeAnalysisTK.jar -T RealignerTargetCreator -R
  PanTro4rmY.fa -o Mother.RTC.intervals
$ java -Xmx4g -jar -T IndelRealigner -I Mother.rmDUP.bam -R PanTro4rmY.fa -
  targetIntervals Mother.RTC.intervals -o Mother.realign.bam
$ samtools index Mother.realigne.bam
$ java -Xmx4g -jar GenomeAnalysisTK.jar -T BaseRecalibrator -I Mother.realign.bam
  -R PanTro4rmY.fa -knownSites Trio.common.vcf -o Mother.recal_data.grp
$ java -Xmx4g -jar GenomeAnalysisTK.jar -T PrintReads -I Mother.realign.bam -R
  PanTro4rmY.fa -BQSR Mother.recal_data.grp -o Mother.recal.bam
```

STEP [vi] Unique Mapped

Extract Unique Mapped Read

Only unique best alignments are used by extracting unique alignments with X0 (number of best hits) = 1 and X1 (number of suboptimal hits) = 0 in SAM tags. Below is a representative screenshot for extraction of unique mapped read.

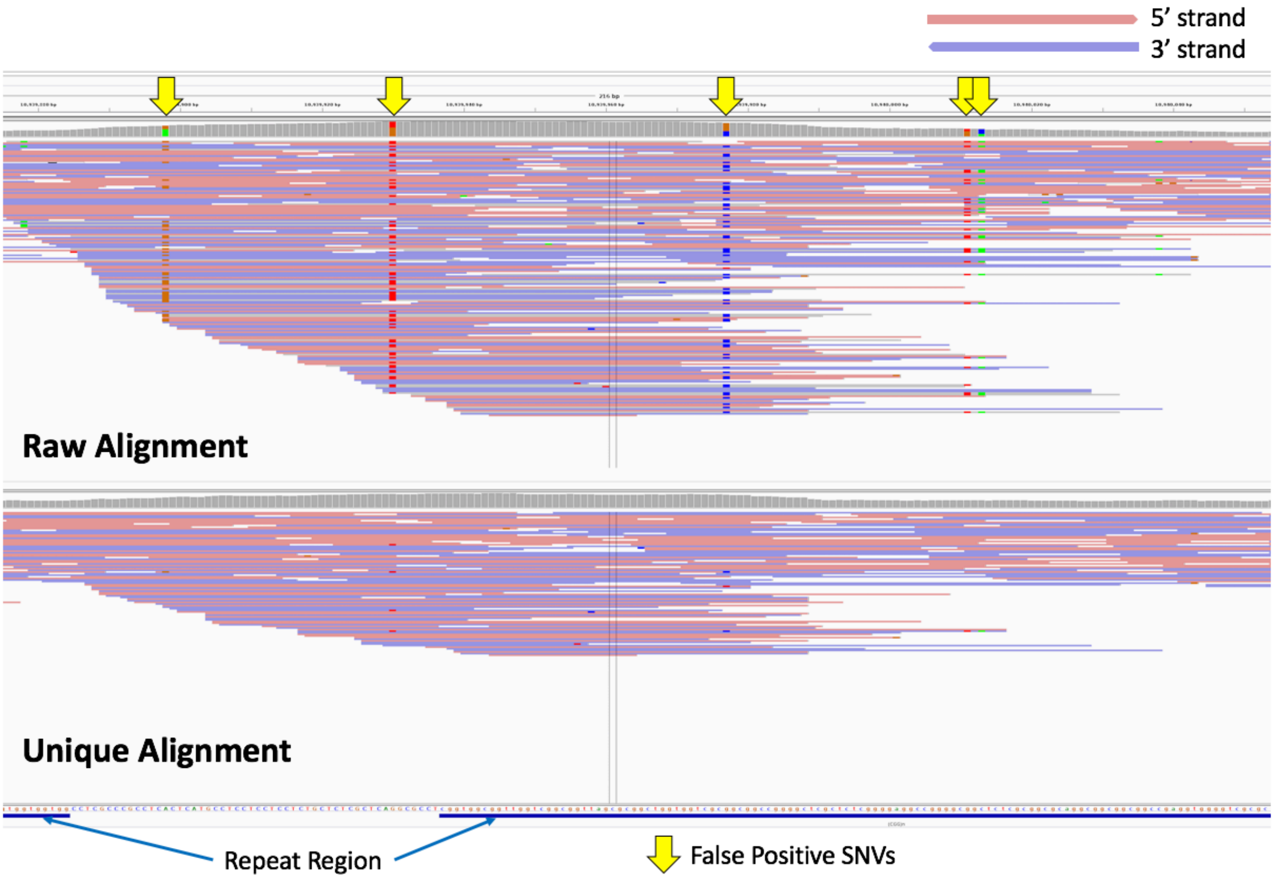

STEP [vii] Calling variants (GATK UnifiedGenotyper)

command:

```
$ java -jar -XX:ParallelGCThreads=1 GenomeAnalysisTK.jar -nct 1 -T
  UnifiedGenotyper -l INFO -R PanTro4rmY.fa -L PanTro4rmY.intervals -I
  Mother.recal.bam -o Mother.Uni_SNV.vcf -stand_call_conf 30 -stand_emit_conf 10
  -dcov maxdepth
$ java -jar -XX:ParallelGCThreads=1 GenomeAnalysisTK.jar -nct 1 -T
  UnifiedGenotyper -glm INDEL -l INFO -R PanTro4rmY.fa -L PanTro4rmY.intervals -I
  Mother.recal.bam -o Mother.Uni_Indel.vcf -stand_call_conf 30 -stand_emit_conf
  10 -dcov maxdepth
```

Discarding low-quality variants

Subsequent filtering of SNVs was performed by discarding low-quality variants according to the score calculated from UnifiedGenotyper analysis; the second most likely phred-scaled likelihoods (PL) – the most likely PL < 200 for heterozygous SNVs and < 100 for homozygous SNVs for reducing FPs

STEP [viii] Custom filter

Filter 1: Read Depth

Read-depth ranges was set to ± 3σ from mean for each individual.

Father: 27× < Read Depth < 251×, Mother: 29× < Read Depth < 199×, Offspring: 34× < Read Depth < 201×

This filter removed **163,077,725 bp** (6.28%).

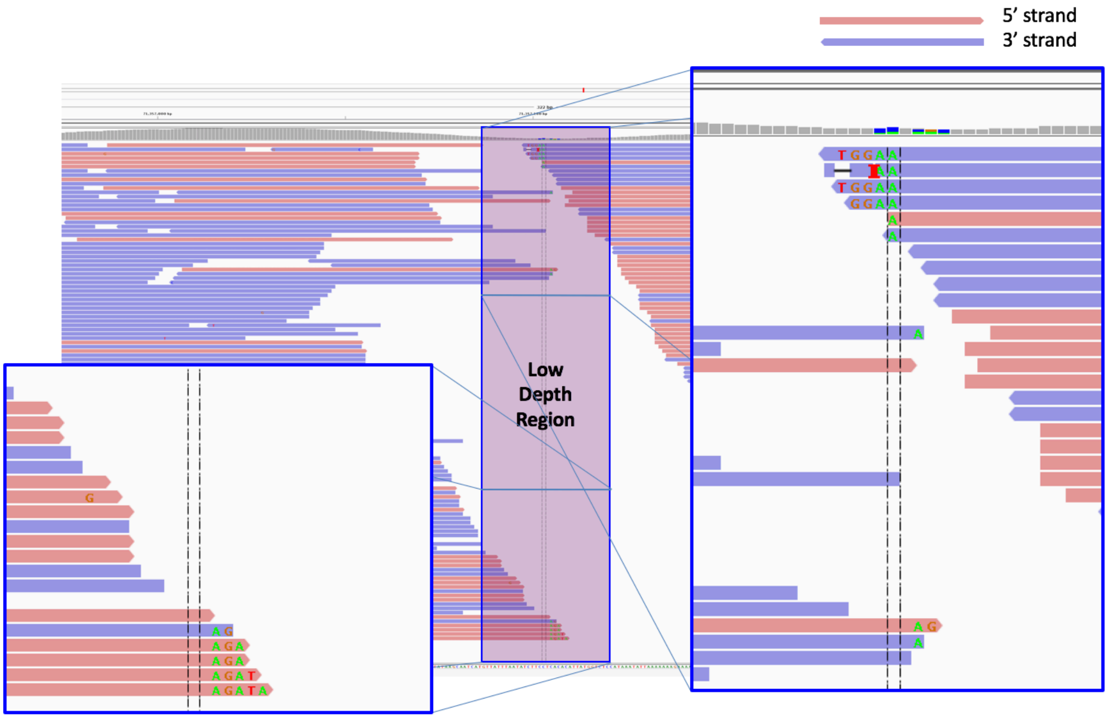

Analysis of the sequencing data by a custom MATLAB script.

Filter 2: Mapped-read balance

Allelic balance of mapped read is considered by extracting the regions with at least 10 forward and reverse reads. This filter removed **190,025,818 bp** (7.32%).

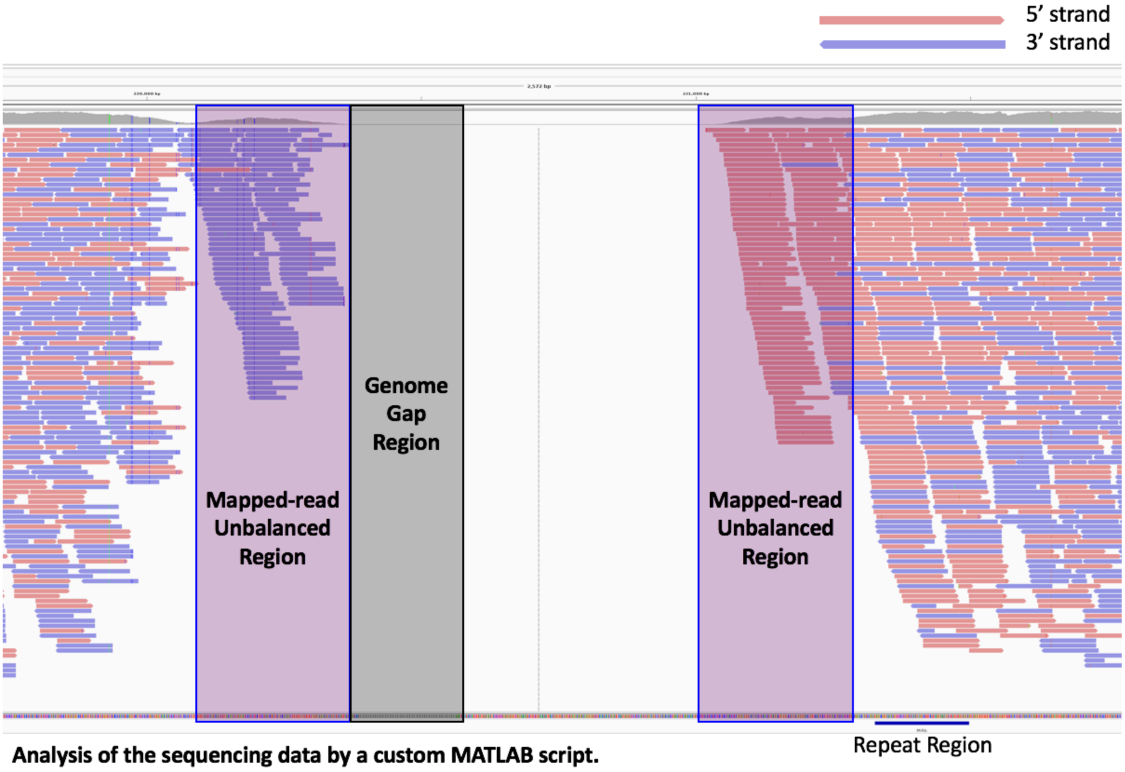

Analysis of the sequencing data by a custom MATLAB script.

Filter 3: Mapped-read balance
The indels and adjacent 50 bp were excluded from target genomic regions. This filter removed **86,308,113 bp** (3.33%).

command:
```
$ java -jar GenomeAnalysisTK.jar -R PanTro4.fa -T VariantFiltration -V
  Trio.snv.vcf -o Trio.snv.fil.vcf -mask Trio.indel.vcf --maskName InDel --
  maskExtension "50"
```

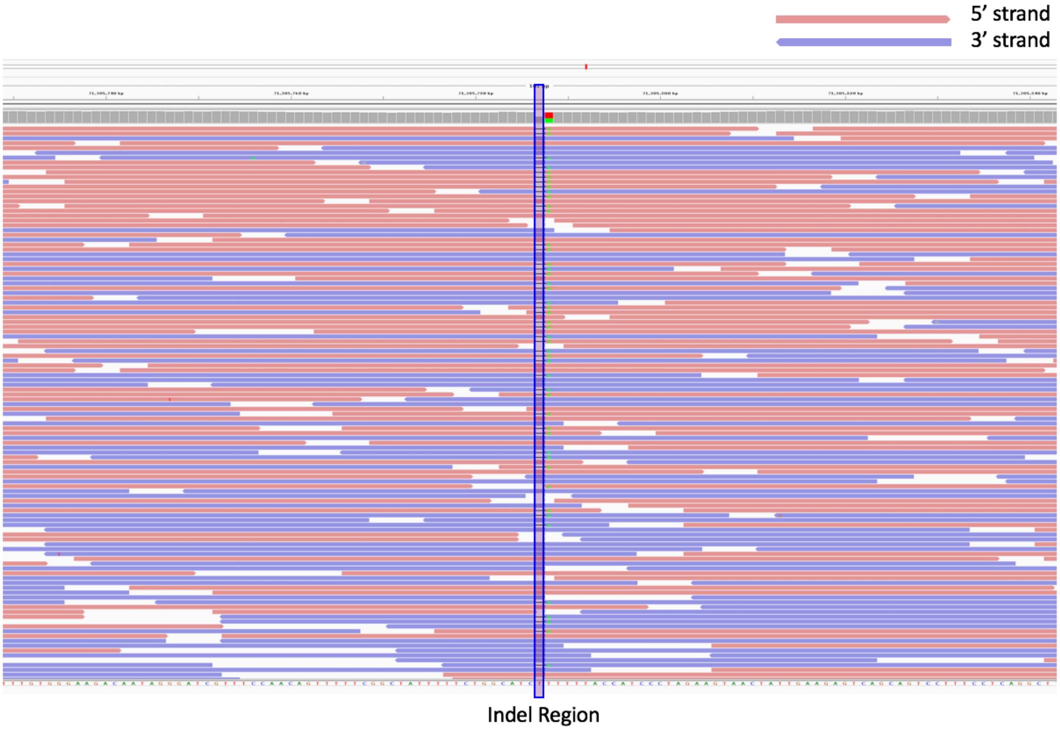

Filter 4: Allelic and strand bias
The variant sites that were covered by at least one read on the reference forward strand, reference reverse strand, alternative forward allele, and alternative reverse allele were retained. All biased SNVs and adjacent 10 bp sites were excluded from the genomic target regions. This filter removed **7,455,688 bp** (0.29%).

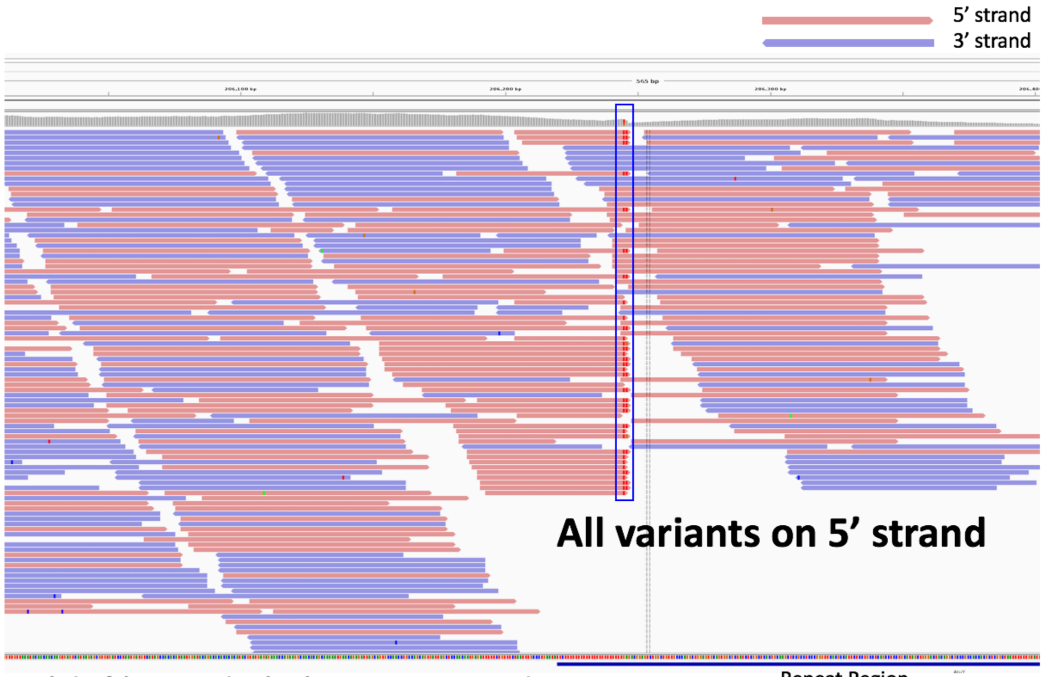

Analysis of the sequencing data by a custom MATLAB script.

Filter 5: Gaps
All variant sites located at the end of the read, with average sizes from the end of read within 10 bp, were excluded from genomic target regions and adjacent 10 bp sites were also excluded. This filter excludes low quality variants at the terminus of each read because the quality of both sides of a read tends to be lower. This filter removed **12,527,318 bp** (0.48%).

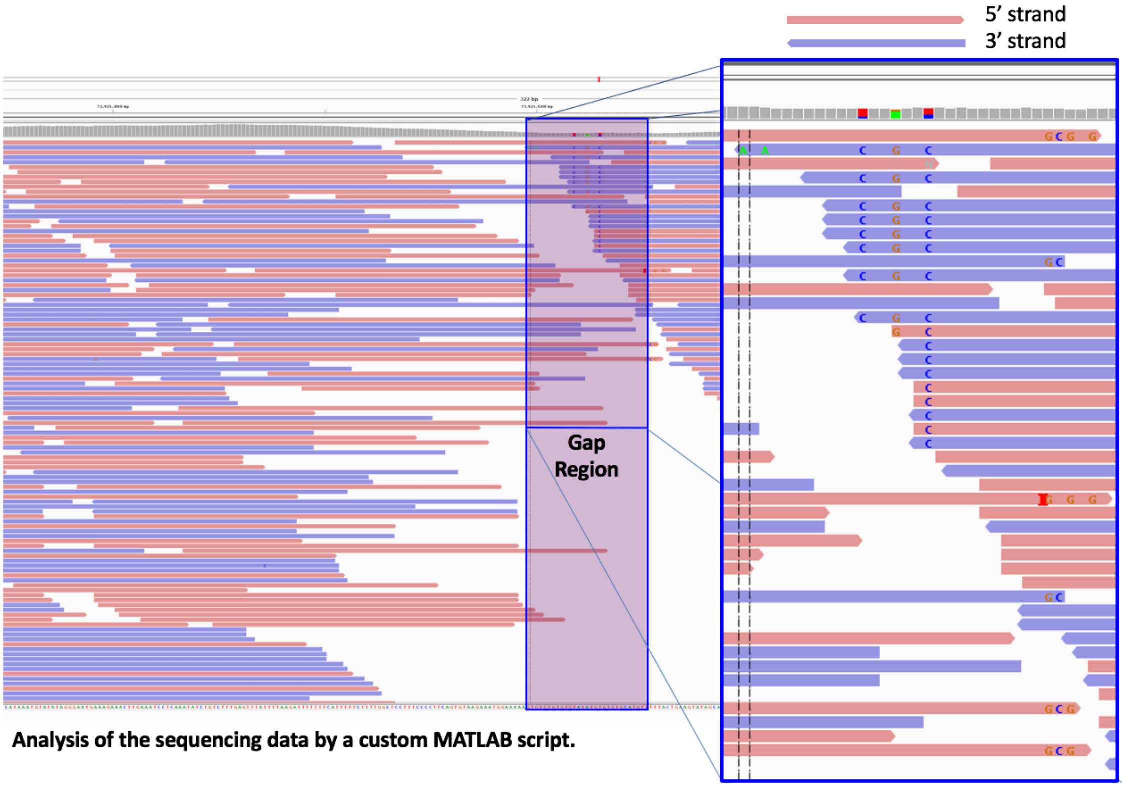

Analysis of the sequencing data by a custom MATLAB script.

We removed **281,326,851 bp** (10.84%) using these five filters and ultimately defined the target genomic regions that were shared among the trio, covering **89.16%** of the chimpanzee reference genome.
